# Widespread mono- and oligoadenylation direct small noncoding RNA maturation versus degradation fates

**DOI:** 10.1038/s44318-025-00655-2

**Published:** 2025-12-05

**Authors:** Cody Ocheltree, Blake Skrable, Anastasia Pimentel, Timothy Nicholson-Shaw, Suzanne R Lee, Jens Lykke-Andersen

**Affiliations:** 1 Department of Molecular Biology, School of Biological Sciences, University of California San Diego, La Jolla, CA 92093 USA; 2 Present Address: Biology Department, Western Washington University, Bellingham, WA 98225 USA

**Keywords:** Small Noncoding RNA, 3’-End Processing, Adenylation, 7SL RNA, Terminal Nucleotidyltransferase 2, RNA Biology

## Abstract

Small noncoding RNAs (sncRNAs) are subject to 3’-end trimming and tailing activities that impact maturation versus degradation decisions during biogenesis. To investigate the dynamics of human sncRNA 3’-end processing at a global level, we performed genome-wide 3’-end sequencing of newly transcribed and steady-state sncRNAs. This revealed widespread post-transcriptional adenylation of newly transcribed sncRNAs, which came in two distinct varieties. One is characterized by oligoadenylation, which is transient, promoted by TENT4A/4B polymerases, and most commonly observed on unstable small nucleolar RNAs that are not fully processed at their 3’-ends. The other is characterized by monoadenylation, which is broadly catalyzed by TENT2 and, in contrast to oligoadenylation, stably accumulates at the 3’-end of sncRNAs, including Polymerase-III-transcribed (Pol-III) RNAs and a subset of small nuclear RNAs. Monoadenylation inhibits Pol-III RNA post-transcriptional 3’-uridine trimming and extension and, in the case of 7SL RNAs, prevents their accumulation with nuclear La protein and promotes their biogenesis towards assembly into cytoplasmic signal recognition particles. Thus, the biogenesis of human sncRNAs involves widespread mono- or oligoadenylation with divergent impacts on sncRNA fates.

## Synopsis

Newly transcribed small non-coding (snc)RNAs undergo maturation or degradation directed by 3’ end trimming and tailing events. This study uncovers widespread mono- and oligo-adenylation during biogenesis of sncRNAs with divergent impacts on maturation versus degradation fates.

- Genome-wide 3’ end sequencing of newly transcribed and steady state human small non-coding RNAs reveals widespread mono- and oligo-adenylation associated with biogenesis.
- Oligoadenylation is promoted by TENT4A/B and associated with partially processed unstable small nucleolar RNAs.
- Monoadenylation is promoted by TENT2, occurs on RNA Polymerase-III transcripts and a subset of small nuclear RNAs, and persists to the steady state.
- Monoadenylation by TENT2 terminates 3’ end uridylation and deuridylation dynamics for RNA Polymerase-III transcripts and promotes 7SL RNA biogenesis.

## Abstract (web summary)

Global 3’ end profiling of small noncoding RNAs shows TENT2-dependent monoadenylation and TENT4A/B-dependent oligo-adenylation during sncRNA biogenesis.

## Graphical abstract

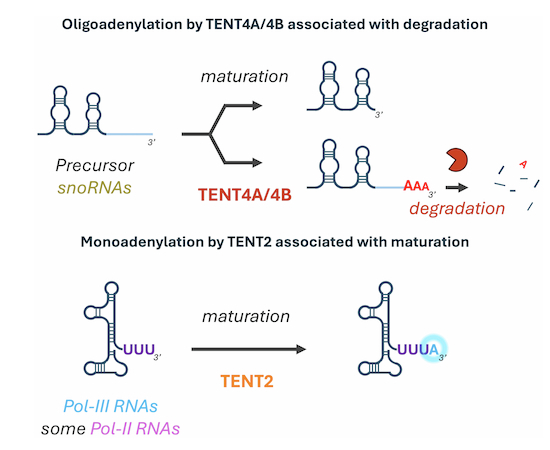

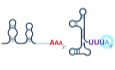

## Introduction

The vast majority of eukaryotic RNAs undergo post-transcriptional processing critical for their biogenesis into functional mature RNAs. RNA 3’-ends in particular are subject to a wide variety of processing events, including exonucleolytic trimming by 3’-to-5’ exonucleases, addition of post-transcriptional nucleotides by polymerases, or 3’ end nucleotide modifications such as 2’,3’ cyclic phosphorylation or 2’O-methylation. Some of the most abundant RNAs in eukaryotic cells are small noncoding RNAs (sncRNAs), which perform a wide variety of critical functions in gene expression. The majority of sncRNAs undergo processing at their 3’-ends after their initial transcription, which can impact sncRNA cellular localization and function. Moreover, 3’-end trimming and tailing towards sncRNA maturation have been observed to occur in competition with degradation from the 3’-end in a process thought to distinguish functional from non-functional molecules (Lardelli et al, 2017; Son et al, 2018; Lardelli and Lykke-Andersen, 2020; Ma et al, 2024; Fatica et al, 2000).

SncRNAs can be divided into distinct classes in terms of how their 3’-ends are initially formed. One consists of sncRNAs transcribed by RNA polymerase-II (Pol-II or Pol2), such as spliceosomal small nuclear (sn)RNAs, whose nascent 3’-ends form via co-transcriptional cleavage by the Integrator complex (Baillat et al, 2005; Egloff et al, 2010; O’Reilly et al, 2014; Rubtsova et al, 2019). This cleavage results in short encoded 3’-end extensions, which subsequently undergo various degrees of trimming (Lardelli et al, 2017; Lardelli and Lykke-Andersen, 2020; Ma et al, 2024). A second class consists of small nucleolar (sno)RNAs and small Cajal-body associated (sca)RNAs that are encoded within introns of protein-coding or noncoding genes (Dieci et al, 2009). The 3’-ends of these sncRNAs are formed by the process of splicing, which is followed by intron debranching and trimming to generate the mature molecules (Filipowicz and Pogačić, 2002). A third class consists of sncRNAs transcribed by RNA Polymerase-III (Pol-III or Pol3), which are terminated at oligo(T) DNA termination sequences (Nielsen et al, 2013; Braglia et al, 2005; Gao et al, 2018). The resulting 3’-uridine termini generally undergo subsequent trimming or, in the case of U6 snRNA, extension to generate the mature RNAs (Simons et al, 1996; Ciganda and Williams, 2011; Yamashita and Tomita, 2023; Chen et al, 1998).

A large number of exonucleases and polymerases participate in the processing of sncRNA 3’-ends. Some of these function in RNA maturation, while others promote degradation. For example, 3’-to-5’ exonucleases TOE1, PARN, and USB1 promote 3’-end maturation of a large variety of sncRNAs (Huynh and Parker, 2023), whereas DIS3 exonucleases, either as components of the exosome (DIS3 or DIS3L in human) (Lykke-Andersen et al, 2011; Tomecki et al, 2010), or acting on its own in the cytoplasm (DIS3L2) (Belair et al, 2018), generally promote RNA degradation. 3’-end tailing by polymerases has also been associated with either maturation or degradation, depending on the polymerase and RNA. For example, oligo-uridylation by Terminal Uridylyltransferases (TUT) 4 and 7 promotes degradation in the cytoplasm (Ustianenko et al, 2016; Pirouz et al, 2016; Łabno et al, 2016b), whereas oligoadenylation of RNAs by Terminal Nucleotidyltransferases TENT4A and TENT4B is associated with degradation by the exosome in the nucleus (Warkocki et al, 2018). However, uridylation and adenylation have also been associated with RNA maturation and stability. For example, uridylation in the nucleus by TUT1 promotes maturation of U6 snRNA (Trippe et al, 2003, 2006). Moreover, monoadenylation by the Terminal Nucleotidyltransferase TENT2 has been observed to stabilize a subset of microRNAs (D’Ambrogio et al, 2012; Katoh et al, 2009), and oligoadenylation has been proposed to promote 3’-end maturation activities of PARN (Son et al, 2018; Moon et al, 2015; Berndt et al, 2012) and TOE1 (Lardelli et al, 2017; Lardelli and Lykke-Andersen, 2020; Ma et al, 2024). However, the general principles that govern these 3’-end trimming and tailing events and how they drive the competition between sncRNA maturation and degradation remain poorly defined.

Many Pol-III RNAs undergo trimming or tailing of the initial 3’-oligouridine termini produced during transcription termination. For U6 snRNA, U-tail extension by TUT1 is terminated by a 2’,3’ cyclic phosphorylation event, which is important for maturation and stability (Yamashita and Tomita, 2023; Didychuk et al, 2017). Another subset of Pol-III RNAs (as well as some snRNAs) has been observed to undergo monoadenylation to varying degrees (Sinha et al, 1998), and this monoadenylation has been observed to oppose 3’-uridylation in human cell extracts and when injected into Xenopus oocyte nuclei (Chen et al, 2000). One example is 7SL RNA, which serves as the central scaffold of the Signal Recognition Particle (SRP) that functions in the co-translational translocation of polypeptides into the endoplasmic reticulum (ER) (Kellogg et al, 2021; Akopian et al, 2013). Biogenesis of 7SL RNA involves assembly with several SRP proteins in the nucleus prior to nuclear export and association with a final SRP protein component, SRP54, in the cytoplasm (Jacobson and Pederson, 1998; Ciufo and Brown, 2000; Grosshans et al, 2001). In mammalian cells, 7SL RNA is known to be processed at the 3’-end with the removal of terminal uridines and the addition of a monoadenosine tail (Ullu and Weiner, 1984; Chen et al, 1998; Sinha et al, 1998), but how this may impact 7SL RNA biogenesis and assembly into the SRP is unknown.

In this study, we investigated the genome-wide dynamics of 3’-end processing of human sncRNAs by sequencing 3’-ends of newly transcribed and steady-state sncRNAs, ≈90–500 nucleotides in length. This revealed widespread sncRNA post-transcriptional A-tailing, which was observed in two forms. One consisted of transient oligo(A)-tails, which were observed primarily on partially processed snoRNAs and scaRNAs, and correlated with instability rather than 3’ end maturation. Another consisted of mono(A)-tailing, which stably accumulated on a majority Pol-III RNA species and a subset of snRNAs. We identified TENT2 as a polymerase that broadly monoadenylates Pol-III RNAs and snRNAs and found that mono(A)-tailing by TENT2 inhibits both uridylation and deuridylation of Pol-III RNAs. Moreover, we present evidence that monoadenylation promotes 7SL RNA biogenesis by inhibiting 7SL RNA interaction with nuclear La protein and promoting the assembly into cytoplasmic SRP particles. These findings identify divergent roles of mono- and oligoadenylation in maturation versus degradation decisions during sncRNA biogenesis.

## Results

### Global analysis of sncRNA 3’-end dynamics reveals transient and stable post-transcriptional A- and U-tails

**Figure 1:**
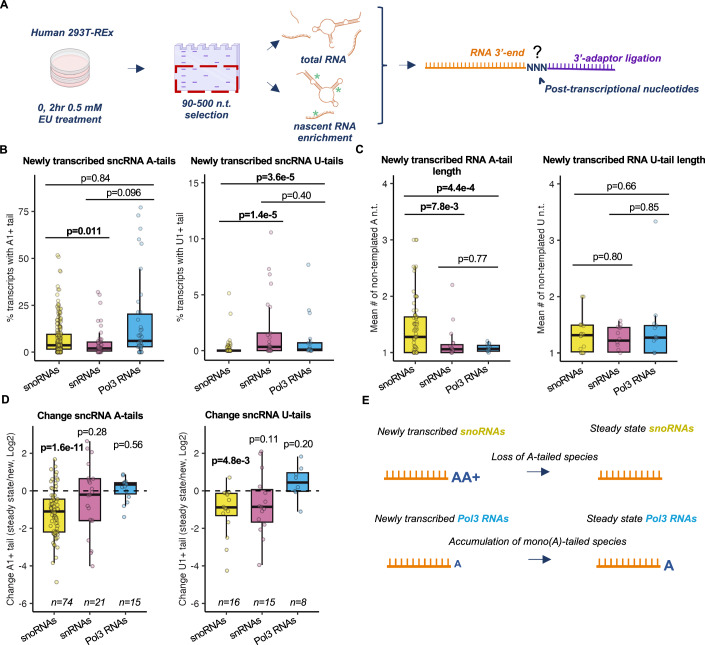
Post-transcriptional A- and U-tails are transient on a subset of sncRNAs but stable on others. (**A**) Schematic of the newly transcribed and steady-state sncRNA global 3’-end sequencing workflow. Human embryonic kidney (HEK) 293T-REx cells were metabolically labeled with 5-ethynyluridine (EU), followed by RNA size-selection and nascent RNA capture. RNA 3’-ends were determined by global RNA 3’-end sequencing of newly transcribed and steady state sncRNA samples (*n* = 3 biological replicates). All snRNAs and sno/scaRNAs annotated in GENCODE V33 were included in these analyses, whereas for Pol-III RNAs, tRNAs, and 5S RNA were excluded via size selection and rRNA depletion to prevent them from diminishing sequencing depth and were therefore not included in analyses (see Dataset EV1 for a full list of included RNAs). Depictions of cell culture, gel extraction, and individual RNAs were created in BioRender (MO27QXAFOF, https://biorender.com/j15w879). (**B**) Percentage of newly transcribed sncRNA species with A- (left) and U-tails (right). RNAs are grouped by their transcriptional origin, and dots represent individual sncRNAs. Box plots show the median (center), the interquartile range as bounds (25th to 75th percentile), and whiskers extending to the most extreme data point within 1.5× the interquartile range from the box, with outliers not shown. *P* values were determined by two-sample Kolmogorov–Smirnov (KS) tests, with *P* < 0.05 shown in bold (snoRNAs *n* = 98, snRNAs *n* = 25, Pol3 RNAs *n* = 19). (**C**) Mean lengths of newly transcribed sncRNA A- (left, snoRNAs *n* = 74, snRNAs *n* = 19, Pol3 RNAs *n* = 15) and U-tails (right, snoRNAs *n* = 18, snRNAs *n* = 14, Pol3 RNAs *n* = 13). Only sncRNA species with A- or U-tails are plotted. Box plots show the median (center), the interquartile range as bounds (25th to 75th percentile), and whiskers extending to the most extreme data point within 1.5× the interquartile range from the box, with outliers not shown. *P* values were determined by two-sample KS tests, with *P* < 0.05 shown in bold. “A n.t.” denotes adenosine nucleotides. (**D**) Log2-fold ratios of percentages of A- (left) and U-tailed (right) sncRNA species in steady state over newly transcribed conditions. In order to avoid division by 0, only RNA species with 0.1% or greater tailing in both newly transcribed and steady state conditions were plotted. Box plots show the median (center), the interquartile range as bounds (25th to 75th percentile), and whiskers extending to the most extreme data point within 1.5× the interquartile range from the box, with outliers not shown. *P* values were determined by a one-sample two-tailed *t* test against mu = 0, with *P* < 0.05 shown in bold. (**E**) Schematic summarizing the 3’-end tail status of newly transcribed snoRNAs and Pol-III RNAs. snoRNAs are modified with short oligo(A) tails which are absent in the steady state, while Pol-III RNAs retain their predominant mono(A) tails in the steady state. Depiction of RNA was created in BioRender (MO27QXAFOF, https://biorender.com/j15w879).

**Figure EV1:**
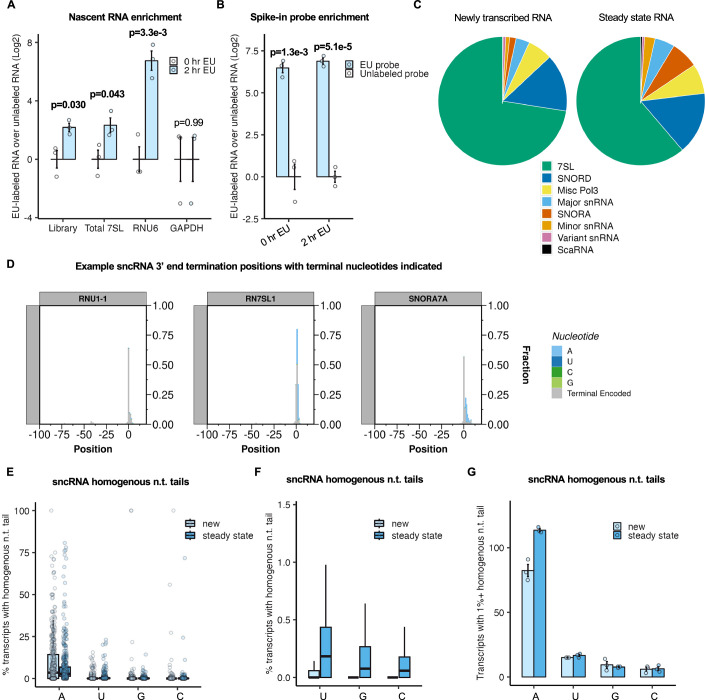
Global enrichment of newly transcribed sncRNAs. (**A**) Efficiency of nascent sncRNA capture and size selection monitored by RT-qPCR for indicated RNAs from 2 h EU- relative to DMSO (0 h) treated samples. The library sample represents the total library monitored by RT-qPCR using primers against ligation linkers. Data is represented as mean +/− SEM and significance was determined by two-sample two-tailed *t* test with *P* < 0.05 shown in bold (*n* = 3 biological replicates per condition). (**B**) Efficiency of nascent RNA capture monitored by RT-qPCR for EU-labeled versus unlabeled spike-in β-globin probes. Data is represented as mean +/− SEM with and significance was determined by two-sample two-tailed *t* test with *P* < 0.05 shown in bold (*n* = 3 biological replicates per condition). (**C**) The mean proportion of reads aligned to each representative sncRNA class in newly transcribed and steady state libraries (*n* = 3 biological replicates for each condition). SNORA and SNORD: box H/ACA and box C/D snoRNAs, respectively. (**D**) The distribution of the 3’ end position for newly transcribed reads aligning to the U1 snRNA, 7SL1, and SNORA7A from an individual biological replicate. The terminal nucleotide is plotted by color as a post-transcriptionally-added A, U, C, or G nucleotide, or plotted in gray if the terminal nucleotide is encoded, as a fraction of total reads for each gene. (**E**) The percent of transcripts with homogenous post-transcriptional A-, U-, G- or C-tails in newly transcribed and steady state conditions (*n* = 142 transcripts per condition, *n* = 3 biological replicates for each condition). Box plots show the median (center), the interquartile range as bounds (25th to 75th percentile), and whiskers extending to the most extreme data point within 1.5× the interquartile range from the box, with outliers not shown. n.t. denotes nucleotide. (**F**) Data from panel *D* plotted as ranges of percentages of transcripts with homogenous post-transcriptional U-, G- and C-tails in newly transcribed and steady state conditions. Box plots show the median (center), the interquartile range as bounds (25th to 75th percentile), and whiskers extending to the most extreme data point within 1.5× the interquartile range from the box, with outliers not shown. n.t. denotes nucleotide. (**G**) Number of unique transcripts with 1% or greater homogenous post-transcriptional nucleotide tails in newly transcribed and steady state conditions. Data is represented as mean +/− SEM (*n* = 3 biological replicates for each condition). n.t. denotes nucleotide.

To globally investigate the dynamics of human sncRNA 3’-end processing, we performed 3’-end sequencing of steady state and newly transcribed sncRNAs, ~90–500 nucleotides in length, from human embryonic kidney (HEK) 293TRex cells (Fig. 1A). Newly transcribed sncRNAs were isolated by click-it chemistry followed by biotin pulldown after two hours of metabolic labeling with 5-ethynyl-uridine. A 2-h incubation was necessary for achieving sufficient enrichment over steady-state sncRNAs and depth of sequencing (Fig. EV1A,B). A wide variety of small RNAs were represented in both steady state and newly transcribed libraries (Fig. EV1C,D). Since RNAs that undergo 3’-end processing during biogenesis are expected to show differences in 3’-end nucleotide composition in the newly transcribed versus the steady state populations, we compared 3’-ends of individual RNAs in each state. We focused the analyses on full-length sncRNAs (terminating at positions −10 to +50 relative to annotated 3’-ends) to avoid confounding effects of sncRNAs that may have been truncated during RNA isolation. We first examined the dynamics of post-transcriptional A- and U-tailing. We hypothesized that sncRNA post-transcriptional processing may be linked to their mechanism of initial 3’-end formation and therefore grouped sncRNAs according to their transcriptional origin as snoRNAs (including box H/ACA snoRNAs, box C/D snoRNAs, and scaRNAs), snRNAs (including spliceosomal snRNAs and U3 and U8 snoRNAs), and Pol-III RNAs.

Post-transcriptional A-tailing was prevalent among newly transcribed sncRNA species, particularly among Pol-III RNAs, some of which saw A-tailing of over 50% of the newly transcribed population (Fig. 1B). SnRNAs and Pol-III RNAs that saw A-tailing were typically mono(A)-tailed, whereas, consistent with previous observations (Berndt et al, 2012; Sinha et al, 1998), snoRNAs experienced significantly longer A-tailing (Fig. 1C). Post-transcriptional uridylation was also observed for a subset of sncRNAs but at a much lower frequency than A-tailing, and was significantly less frequent among snoRNAs than among snRNAs and Pol-III RNAs (Fig. 1B,C). Of note, post-transcriptional uridylation of Pol-III RNAs can be difficult to detect given their oligouridine termination sequences, which cannot be distinguished from post-transcriptional uridines unless U-tails are very long (see further analyses below). Post-transcriptional cytosine and guanosine tails were detectable at some sncRNAs but were very rare and were not further investigated here (Fig. EV1E–G).

To monitor the dynamics of sncRNA A- and U-tailing, we next compared A- and U-tails in steady state versus newly transcribed conditions. This revealed different dynamics for sncRNA species from different transcriptional origins. For snoRNAs, post-transcriptional A- and U-tailing was overwhelmingly transient, as evidenced by a significant shortening of A- and U-tails in steady state compared to newly transcribed conditions (Fig. 1D). By contrast, post-transcriptional A- and U-tails were generally stable for Pol-III RNAs, while snRNAs displayed more of a mixture of transient and stable tails dependent on the RNA species (Dataset EV3). Thus, A-tailing is widespread among sncRNAs, with Pol-III RNAs and some snRNAs showing subpopulations with short A-tails that accumulate to the steady state, whereas snoRNAs show longer A-tails that are overwhelmingly transient (Fig. 1E).

### Transient A-tailing of snoRNAs is associated with instability

**Figure 2:**
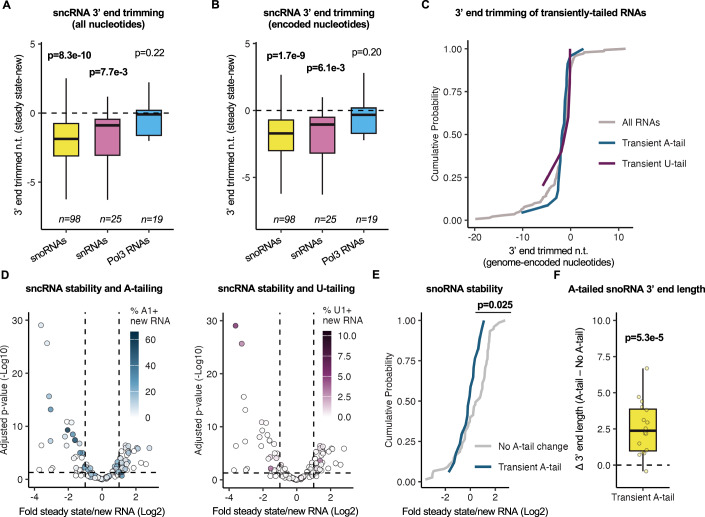
Transient A-tailing is associated with the destabilization of snoRNAs. (**A**, **B**) Box plots showing ranges of sncRNA 3’-trimming as measured by the difference in the mean 3’-end positions of transcripts in steady state versus newly transcribed RNA populations. Transcripts were binned by their transcriptional origin. Values in (**B**) exclude nucleotides from post-transcriptional tails. Box plots show the median (center), the interquartile range as bounds (25th to 75th percentile), and whiskers extending to the most extreme data point within 1.5× the interquartile range from the box, with outliers not shown. *P* values were determined by a one-sample two-tailed *t* test against mu = 0, with *P* < 0.05 shown in bold. “n.t.” denotes nucleotides. (**C**) Cumulative plot showing sncRNA 3’-trimming as measured by the difference in the mean 3’-end positions of transcripts in steady state versus newly transcribed RNA populations. SncRNAs that saw transient 3’ A-or U-tailing, as measured by a significantly (*P* < 0.05; two-sample two-tailed *t* test) higher fraction of A- or U-tailed molecules in newly transcribed over steady state populations, are compared to all RNAs (All RNAs *n* = 142, transient A-tails *n* = 23, transient U-tails *n* = 5). “n.t.” denotes nucleotides. (**D**) Volcano plot comparing sncRNA stability with the percentages of A- (left) or U-tailed (right) molecules. The log2-fold ratio of sncRNA levels in steady state over newly transcribed conditions was quantified with DESeq2 and plotted against −log10-converted Benjamini–Hochberg-adjusted *P* values from DESeq2. Individual sncRNAs (*n* = 142) are colored according to their percentage of modification with A- or U-tails of any length. The horizontal dashed line represents *P* = 0.05. (**E**) Cumulative plot showing snoRNA stability as measured by the log2 ratio of steady state over newly transcribed levels. snoRNAs with significantly higher A-tailing in the newly transcribed population relative to steady state are compared to all other snoRNAs. *P* value was determined by a two-sample KS test (no A-tail change *n* = 75, transient A-tail *n* = 17). (**F**) Box plot showing the mean position of snoRNA A-tails relative to the 3’-end position of their unadenylated counterparts for transiently A-tailed snoRNAs. Box plots show the median (center), the interquartile range as bounds (25th to 75th percentile), and whiskers extending to the most extreme data point within 1.5× the interquartile range from the box, with outliers not shown. *P* value was determined by a one-sample two-tailed *t* test against mu = 0 (*n* = 17).

**Figure EV2:**
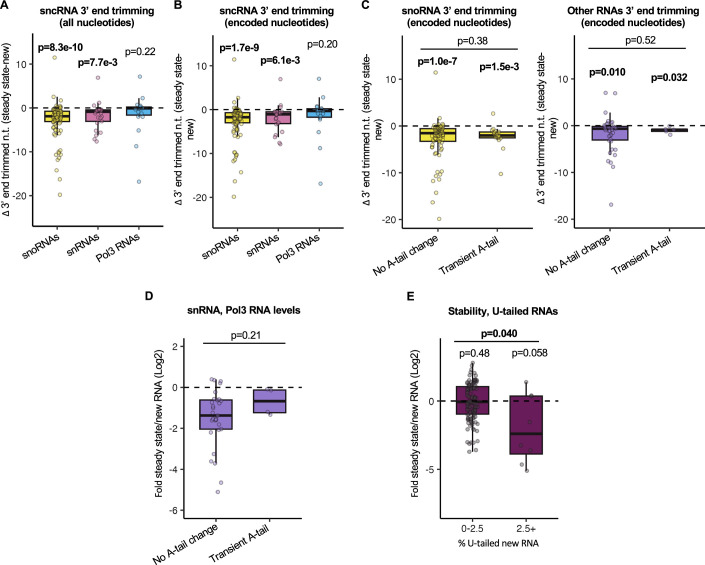
3’ end trimming and stability of tailed sncRNAs. (**A**) Plot from Fig. 2A with individual transcripts visualized (snoRNAs *n* = 98, snRNAs *n* = 25, Pol3 RNAs *n* = 19). Box plots show the median (center), the interquartile range as bounds (25th to 75th percentile), and whiskers extending to the most extreme data point within 1.5× the interquartile range from the box, with outliers not shown. *P* values were determined by a one-sample two-tailed *t* test against mu = 0, with *P* < 0.05 shown in bold. “n.t.” denotes nucleotides. (**B**) Plot from Fig. 2B with individual transcripts visualized (snoRNAs *n* = 98, snRNAs *n* = 25, Pol3 RNAs *n* = 19). Box plots show the median (center), the interquartile range as bounds (25th to 75th percentile), and whiskers extending to the most extreme data point within 1.5× the interquartile range from the box, with outliers not shown. *P* values were determined by a one-sample two-tailed *t* test against mu = 0, with *P* < 0.05 shown in bold. “n.t.” denotes nucleotides. (**C**) Box plots showing 3’ end trimming of snoRNAs (left: no A-tail change *n* = 80 RNA species, transient A-tail *n* = 18 RNA species) and snRNAs/Pol-III RNAs (right: no A-tail change *n* = 39 RNA species, transient A-tail *n* = 5 RNA species) as measured by the difference in the mean 3’-end positions of transcripts in steady state versus newly transcribed RNA populations. RNAs that saw transient 3’ A-tailing, as measured by a significantly (*P* < 0.05; two-sample two-tailed *t* test) higher fraction of A-tailed molecules in newly transcribed over steady state populations, are compared to other RNAs. Box plots show the median (center), the interquartile range as bounds (25th to 75th percentile), and whiskers extending to the most extreme data point within 1.5× the interquartile range from the box, with outliers not shown. Significance for each group were determined by a one-sample two-tailed *t* test against mu = 0 with *P *< 0.05 in bold. Significance between groups was determined by a two-sample KS test with *P* < 0.05 in bold. “n.t.” denotes nucleotides. (**D**) Box plots showing relative stabilities of snRNA/Pol-III RNAs as measured by log2 ratios of levels in steady state over newly transcribed conditions quantified using DESeq2. RNAs that saw transient 3’ A-tailing, as measured by a significantly (*P* < 0.05; two-sample two-tailed *t* test) higher fraction of A-tailed molecules in newly transcribed over steady state populations, are compared to other RNAs. Box plots show the median (center), the interquartile range as bounds (25th to 75th percentile), and whiskers extending to the most extreme data point within 1.5× the interquartile range from the box, with outliers not shown. Significance between groups was determined by a two-sample KS test with *P* < 0.05 in bold (no A-tail change *n* = 29 RNA species, transient A-tail *n* = 4 RNA species). (**E**) Same as panel *D* but binning sncRNAs by % U-tailing. Box plots show the median (center), the interquartile range as bounds (25th to 75th percentile), and whiskers extending to the most extreme data point within 1.5× the interquartile range from the box, with outliers not shown. Significance for each group were determined by a one-sample two-tailed *t* test against mu = 0 with *P* < 0.05 in bold. Significance between groups was determined by a two-sample KS test with *P* < 0.05 in bold (0-2.5% *n* = 121 RNA species, 2.5%+ *n* = 8 RNA species).

Post-transcriptional A- and U-tailing has previously been associated with promotion of RNA 3’-end trimming (Lardelli et al, 2017; Son et al, 2018; Lardelli and Lykke-Andersen, 2020; Moon et al, 2015; Berndt et al, 2012; Shukla and Parker, 2017) or RNA degradation (Lardelli and Lykke-Andersen, 2020; Tseng et al, 2015; Shukla and Parker, 2017; Sudo et al, 2016), but the principles that dictate one fate over another remain unclear. To first analyze whether A- or U-tailing correlates with 3’-end trimming for each class of sncRNAs, we assessed sncRNA 3’-end trimming by monitoring changes in 3’-ends of sncRNAs in the steady state compared to the newly transcribed population. As expected, we observed significant 3’-end shortening of a majority of snRNAs and snoRNAs, while a majority of Pol-III sncRNAs saw little 3’-end trimming overall (Figs. 2A and  EV2A). This trimming was not restricted to the removal of post-transcriptional tails as it generally extended into the genome-encoded sequence (Figs. 2B and  EV2B). If 3’-end trimming is promoted by A- or U-tailing it is predicted that RNAs that show 3’-trimming also show A- or U-tailing that is transient. To test this prediction, we classified RNAs with transient A- or U-tailing as those that displayed a significantly (*P* < 0.05 by *t* test) higher fraction of A- or U-tailed molecules in the newly transcribed population as compared to the steady state. This analysis revealed no significant difference between the 3’-end trimming of transiently A- or U-tailed sncRNAs and the sncRNA population as a whole (Figs. 2C and  EV2C).

We next assessed whether a correlation exists between 3’-end tailing and sncRNA stability. RNAs that are unstable are expected to be enriched in the newly transcribed population over the steady state. We therefore used the ratio of RNA abundance in the steady state versus newly transcribed populations as a proxy for RNA stability. Multiple sncRNAs that are unstable by this measure showed high levels of A- or U-tailing in the newly transcribed population (Fig. 2D). Evaluating whether a correlation exists between transient tailing and stability for individual groups of sncRNAs, in the case of snoRNAs, those that underwent transient A-tailing showed significantly lower steady state to newly transcribed RNA ratios than the remainder of the snoRNA population suggesting that the transiently A-tailed population is unstable (Fig. 2E). Consistent with previous observations by others (Allmang et al, 1999; van Hoof et al, 2000; Berndt et al, 2012), the transient snoRNA A-tails were found on snoRNAs not fully processed at their 3’-ends (Fig. 2F), suggesting that oligo(A)-tailing and degradation targets snoRNAs that experience stalling in 3’-end processing. In contrast to a previous study suggesting that oligoadenylation is specific to H/ACA box snoRNAs (Berndt et al, 2012), we observed transient oligoadenylation of snoRNAs of all types (Table EV2). Other classes of sncRNAs showed very few transiently A-tailed species and no evidence of associated instability (Fig. EV2D). While there were too few transiently U-tailed sncRNAs to be analyzed in a similar manner, the few sncRNAs that were uridylated at 2.5% or greater ratios in the newly transcribed population were, as a group, significantly less stable than other sncRNAs (Fig. EV2E). Thus, transient A-tailing is observed on partially processed snoRNAs and is correlated with instability, whereas we observe no evidence for a general correlation between A- or U-tailing and sncRNA 3’-end trimming.

### A majority of Pol-III RNA species see subpopulations with mono(A)-tails that accumulate to the steady state

**Figure 3:**
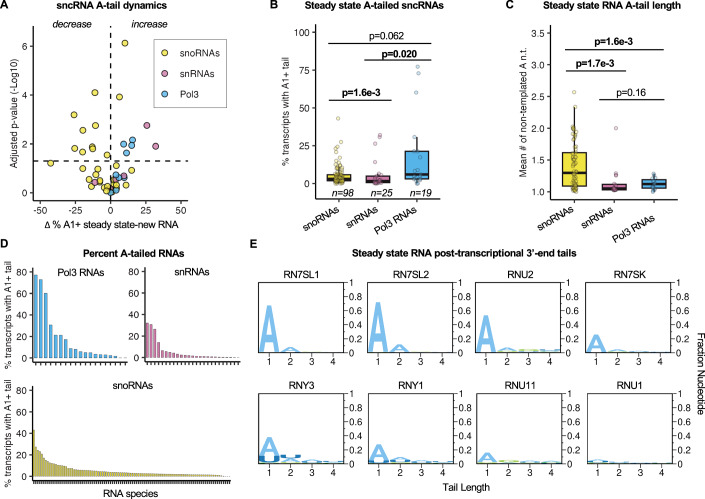
A subset of Pol-III RNAs and snRNAs stably accumulates with mono-A tails. (**A**) Volcano plot showing differences in percentages of A-tailed sncRNAs in the steady state versus newly transcribed conditions versus the −Log10 *P* value of the difference. In order to evaluate sncRNAs with appreciable levels of accumulating A-tailing, only sncRNA species with ≥5% A-tailing in the steady state are plotted (snoRNAs *n* = 31, snRNAs *n* = 6, Pol3 RNAs *n* = 10). Horizontal line indicates *P* = 0.05 by a two-sample two-tailed *t* test. Transcripts are colored by their transcriptional origin. (**B**) Box plots showing ranges in percentages of sncRNAs with A-tails at steady state. Transcripts were binned by their transcriptional origin. Box plots show the median (center), the interquartile range as bounds (25th to 75th percentile), and whiskers extending to the most extreme data point within 1.5× the interquartile range from the box, with outliers not shown. *P* values determined by two-sample KS tests, with *P* < 0.05 shown in bold. (**C**) The mean length of steady-state A-tails of transcripts. Only sncRNA species with A-tails are plotted. Transcripts are binned by their transcriptional origin. Box plots show the median (center), the interquartile range as bounds (25th to 75th percentile), and whiskers extending to the most extreme data point within 1.5× the interquartile range from the box, with outliers not shown. *P* values were determined by the two-sample KS test, with *P* < 0.05 shown in bold (snoRNAs *n* = 74, snRNAs *n* = 19, Pol3 RNAs *n* = 15). (**D**) Rank order plot of sncRNAs by the mean percentage of the population with A-tails at steady state from *n* = 3 biological replicates. Transcripts were plotted separately by their transcriptional origin. (**E**) Logo plots showing mean post-transcriptional tails at steady state for gene-specific sequencing of Pol-III RNAs and snRNAs from *n* = 3 biological replicates. Post-transcriptional nucleotides are plotted as fractions of the total population.

We next turned our attention to the short A-tails observed on Pol-III RNAs and snRNAs. In contrast to snoRNAs, which typically see transient A-tails, a large majority of observed Pol-III RNA species, as well as a subset of snRNA species, accumulate post-transcriptional A-tails at equal or higher levels at steady state than in the newly transcribed population, suggesting that these tails are stable (Figs. 1D and 3A). Pol-III RNAs as a group accumulated with A-tails at steady state at a higher level than other sncRNAs (Fig. 3B), and these A-tails were generally mono(A)-tails (Fig. 3C). While a majority of Pol-III RNA species can be observed with post-transcriptional A-tails at steady state, only three (7SL1, 7SL2 and 7SL3 RNAs) accumulate with A-tails on more than half of their population (Fig. 3D; Dataset EV4). Performing the same analysis for U-tailing revealed a subset of snRNAs that accumulate U tails in the steady state, as well as a select few snRNAs that see U-tails that are transient (Appendix Fig. S1).

To confirm the mono(A)-tailing observed in our global 3’-end sequencing data, we monitored 3’-ends of a subset of Pol-III RNAs and snRNAs by gene-specific 3’-end sequencing, with U1 snRNA serving as a mostly unadenylated control. Plotting the composition of post-transcriptional tails for these RNAs support the conclusion that these RNAs see 3’ monoadenylation at the steady state, ranging from ≈15% of the population for U11 snRNA to ≈70% for 7SL1 and 7SL2 RNAs (Fig. 3E). These levels of monoadenylation are consistent with previous observations for individually tested Pol-III RNAs and snRNAs using 3’-end radioactive labeling methods (Sinha et al, 1998). As expected, the U1 snRNA negative control showed little post-transcriptional 3’-end tailing at steady state. These observations, taken together, demonstrate that mono(A) tails stably accumulate to the steady state on a subset of the population of a majority of Pol-III RNA species, as well as a subset of snRNAs.

### Terminal nucleotidyltransferase 2 (TENT2) promotes Pol-III and snRNA 3’-end mono(A)-tailing

**Figure 4:**
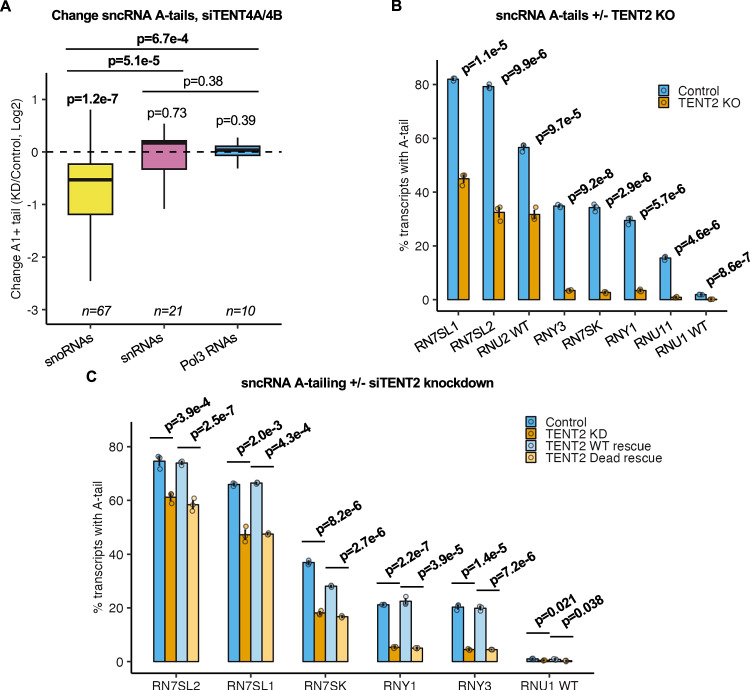
TENT2 contributes to Pol3 and snRNA 3’-end A-tailing. (**A**) Box plots showing Log2 ratios in percentages of A-tailed sncRNAs in TENT4A/4B knockdown conditions over control conditions from a publicly available dataset (*n* = 2 biological replicates for each condition, data from Lim et al, 2018) enriched for RNAs >200 nucleotides. Transcripts are grouped by their transcriptional origin. Box plots show the median (center), the interquartile range as bounds (25th to 75th percentile), and whiskers extending to the most extreme data point within 1.5× the interquartile range from the box, with outliers not shown. *P* values for individual groups were determined by one-sample two-tailed *t* tests against mu = 0. *P* values between groups were determined by two-sample KS tests. *P *< 0.05 is shown in bold. (**B**) Percentage of adenylated sncRNAs in TENT2 KO or control conditions in HEK 293T-Rex cells as measured for select Pol-III RNAs and snRNAs by gene-specific 3’-end sequencing. Data are represented as mean +/− standard error of the mean (SEM), and *P* values were determined by two-sample two-tailed *t* tests, with *P *< 0.05 shown in bold (*n* = 3 biological replicates for each condition). (**C**) Percentage of adenylated sncRNAs in TENT2 siRNA depletion or control conditions in HEK 293T-Rex cells or in TENT2-depleted HEK 293T-Rex cells complemented with tetracycline-induced TENT2 wild-type or TENT2 catalytic dead proteins. Data are represented as mean +/− SEM, and *P* values were determined by two-sample two-tailed *t* tests, with *P* < 0.05 shown in bold (*n* = 3 biological replicates for each condition).

**Figure EV3:**
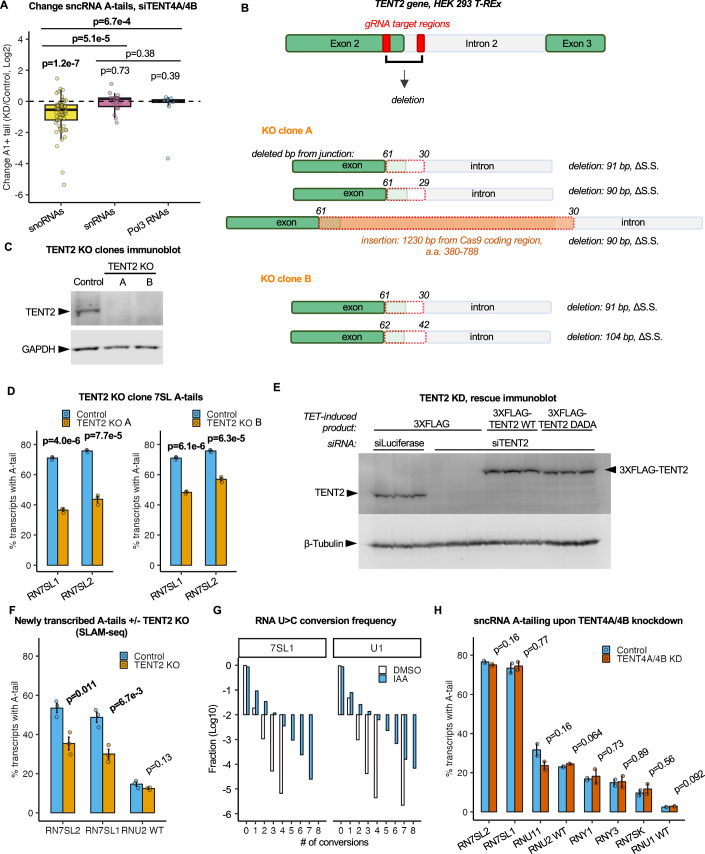
The impact of TENT2 and TENT4A/4B depletion on sncRNA A-tailing. (**A**) Plot from Fig. 4A with individual transcripts visualized (snoRNAs *n* = 67, snRNAs *n* = 21, Pol3 RNAs *n* = 10). Box plots show the median (center), the interquartile range as bounds (25th to 75th percentile), and whiskers extending to the most extreme data point within 1.5× the interquartile range from the box, with outliers not shown. *P* values for individual groups were determined by one-sample two-tailed *t* tests against mu = 0. *P* values between groups were determined by two-sample KS tests. *P* < 0.05 is shown in bold. (**B**) Schematic of *TENT2* genomic region targeted by CRISPR-Cas9 guide (g)RNAs. *TENT2* gene alleles in KO clones A and B determined by PCR amplification of the surrounding region followed by Sanger and nanopore sequencing are illustrated; numbers above alleles refer to the position of the genomic deletion relative to the exon 2-intron2 5’ splice site. S.S. denotes splice site, bp denotes base pair, a.a. denotes amino acid. (**C**) Western blot for TENT2 in TENT2 KO clones A and B and a control clone that saw no TENT2 depletion. (**D**) Percent A-tailing of 7SL RNAs for two TENT2 KO clones. Data is represented as mean +/− SEM and significance was determined by two-sample two-tailed *t* test with *P* < 0.05 in bold (*n* = 3 biological replicates for each condition). Clone A was used in all subsequent analyses. (**E**) Western blot of TENT2 protein following siRNA knockdown in 293T-REx cells expressing exogenous 3XFLAG- or 3XFLAG-TENT2 in biological triplicate. 3XFLAG-TENT2 WT or catalytic mutant (DADA) were induced with tetracycline. β-tubulin is shown as a loading control. (**F**) Percent A-tailing of newly transcribed 7SL and U2 RNAs from control or TENT2 KO cells metabolically labeled with s4U. Reads containing 2 or more U > C conversions after IAA-treatment were considered newly transcribed. Data is represented as mean +/− SEM and significance was determined by two-sample two-tailed *t* test with *P* < 0.05 in bold (*n* = 3 biological replicates for each condition). (**G**) The percent of reads with 0 to 8 U > C conversions for 7SL1 and U1 RNAs after DMSO or IAA-treatment (*n* = 1 biological replicate for each condition). No bar represents none detected. (**H**) Percent A-tailing of select Pol-III/snRNAs during control or TENT4A/4B knock-down (KD) conditions (*n* = 2 biological replicates for each condition, data from Lim et al, 2018). Data is represented as mean +/− SEM. Source data are available online for this figure

To better understand how mono(A)-tailing may impact sncRNA processing or function, we sought to identify the enzymatic activity responsible for this modification. Terminal Nucleotidyl Transferases TENT4A and TENT4B have been previously observed to adenylate a large number of sncRNAs (Tseng et al, 2015; Shukla and Parker, 2017; Wlotzka et al, 2011; Son et al, 2018; Berndt et al, 2012). To assess whether TENT4A/4B are responsible for the observed A-tailing of sncRNAs, we analyzed a published TENT4A/4B knockdown 3’-end RNA-seq dataset (Lim et al, 2018). Plotting the changes in sncRNA 3’-adenylation in control versus TENT4A/4B co-depletion conditions revealed that adenylation of snoRNAs was significantly decreased during TENT4A/4B knockdown (Figs. 4A and  EV3A). This is consistent with previous reports of snoRNA adenylation by TENT4B (Son et al, 2018; Berndt et al, 2012). By contrast, snRNA and Pol-III RNA A-tailing were not significantly impacted. Taken together, these observations implicate TENT4A/4B in the transient A-tailing of snoRNAs, while the short A-tails observed on Pol-III RNAs and a subset of snRNAs appear to be added by different polymerase(s).

**Figure EV4:**
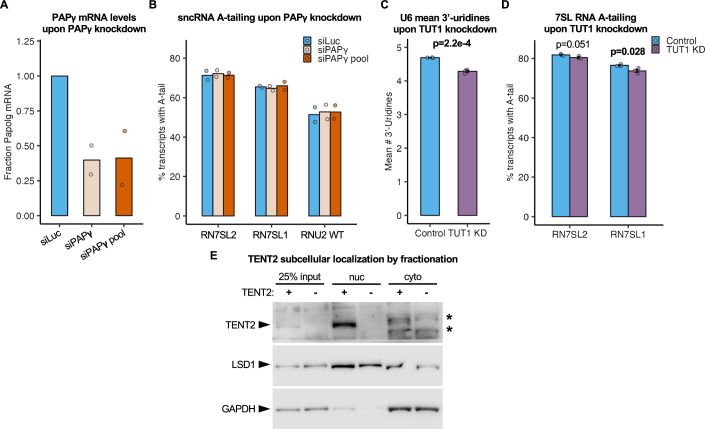
Contributions of polymerases to sncRNA A-/U-tailing. (**A**) Impact of PAP**γ** siRNA knockdown in HEK 293T-REx cells on Papolg mRNA levels monitored by RT-qPCR. A single siRNA against Papolg (siPAPg.1) was used in the siPAP**γ** condition, whereas two siRNAs against Papolg (siPAPg.1, siPAPg.2, sequences from Bresson 2013 (Bresson and Conrad, 2013), Table EV1) were pooled in the siPAPγ pool condition. Levels were normalized against U1 snRNA. Data is represented as mean (*n* = 2 biological replicates for each condition). (**B**) Percent A-tailing of select Pol-III/snRNAs during control or PAP**γ** knockdown conditions. Data is represented as mean (*n* = 2 biological replicates for each condition). (**C**) Impact of TUT1 siRNA knockdown in HEK 293T-REx cells on the mean number of 3’-uridines for the known target U6 snRNA as a functional validation of TUT1 knockdown. Data is represented as mean +/− SEM and significance between groups was determined by two-sample two-tailed *t* test with *P* < 0.05 in bold (*n* = 3 biological replicates for each condition). (**D**) Percent A-tailing of 7SL RNAs during control or TUT1 knockdown (KD) conditions. Data is represented as mean +/− SEM and significance between groups was determined by two-sample two-tailed *t* test with *P* < 0.05 in bold (*n* = 3 biological replicates for each condition). (**E**) Fractionation of Control (+) and TENT2 KO (−) cells into nuclear (nuc) and cytoplasmic (cyto) fractions. Input represents 25% of the fractionated material. LSD1 was used as a nucleoplasmic marker, while GAPDH was used as a cytoplasmic marker. Asterisks indicate non-specific bands produced by the TENT2 antibody. Source data are available online for this figure

Previous studies identified the mouse protein Germ Line Development 2 (mGLD2) as an enzyme that participates in monoadenylation of 7SL RNA (Katoh et al, 2009). To test whether the human homolog of mGLD2, TENT2, promotes sncRNA mono(A)-tailing, we knocked out the *TENT2* gene in HEK 293T-REx cells. Following confirmation of *TENT2* knockout (Fig. EV3B,C), we performed gene-specific 3’-end sequencing of the subset of Pol-III RNAs and snRNAs that presented with at least 20% of the population with post-transcriptional adenylation at steady state. This revealed a significant reduction in 3’-adenylation for all Pol-III RNAs and snRNAs tested (Figs. 4B and  EV3D). Adenylation was almost completely abolished for 7SK, Y1, Y3, and U11 RNAs, whereas for 7SL1, 7SL2, and U2 RNAs approximately half of the original 3’-adenylated population remained after TENT2 knockout. The impact of TENT2 on Pol-III RNA and snRNA adenylation was confirmed by siRNA-mediated depletion of TENT2 and, additionally, by add-back of exogenous wild-type TENT2 versus catalytically inactive TENT2 (Figs. 4C and  EV3E). 3’-end sequencing of newly transcribed 7SL and U2 RNAs isolated from TENT2 knockout and control cells revealed a similar impact on 3’-adenylation as observed at steady state, showing that the adenylated population resistant to TENT2 KO does not reflect a highly stable population generated prior to TENT2 KO (Fig. EV3F,G). Thus, snRNAs and Pol-III RNAs are mono-adenylated by TENT2, with a subset of the 7SL and U2 RNA population adenylated by an additional unknown enzyme, which is not TENT4A/4B (Fig. EV3H). Poly(A)-polymerase γ (PAPγ) has been previously implicated in 7SL RNA adenylation (Perumal et al, 2001), but we observed no impact on 7SL adenylation upon PAPγ depletion (Fig. EV4A,B). Similarly, TUT1 (also known as STAR-PAP) has previously been implicated in RNA adenylation (Mellman et al, 2008; Li et al, 2013). Performing knockdown of TUT1 successfully reduced the mean number of 3’-uridines for the known target U6 snRNA (Yamashita and Tomita, 2023; Trippe et al, 2006), but adenylation was only very modestly reduced for 7SL1 and 7SL2 RNAs (Fig. EV4C,D). Thus, the additional polymerase(s) adenylating 7SL and U2 RNAs remain to be identified.

### Monoadenylation by TENT2 inhibits trimming and extension of Pol-III RNA 3’-uridine tails

**Figure 5:**
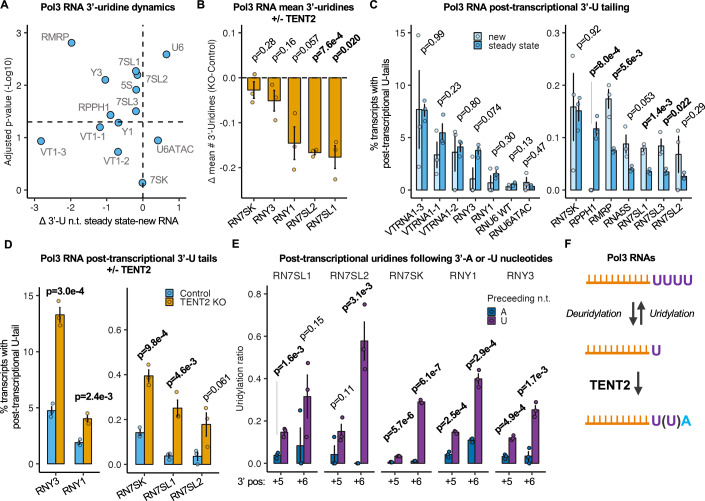
Mono(A) tails inhibit trimming and tailing of Pol3 RNA oligo(U)-termini. (**A**) Differences in lengths of 3’-uridine termini of steady state versus newly transcribed Pol-III RNAs plotted against the −Log10 *P* value for three independent experiments. *P* values were determined by two-sample two-tailed *t* tests. The horizontal line represents *P* = 0.05 (*n* = 3 biological replicates for each transcript). “n.t.” denotes nucleotides. (**B**) Difference in mean number of 3’-uridines of Pol-III RNAs between TENT2 KO and control cells. Data are represented as mean +/− SEM and *P* values were determined by one-sample two-tailed *t* tests against mu = 0 with *P* < 0.05 shown in bold (*n* = 3 biological replicates for each transcript). (**C**) Percentage of Pol-III transcripts with post-transcriptional 3’-U tails in newly transcribed or steady state conditions. Only uridine(s) following the final uridine of their termination sequence are plotted. A termination sequence length of 4 was used for 7SL1-3, 7SK, Y1, Y3, VTRNA1-1, VTRNA1-2, and VTRNA1-3. A termination length of 5 was used for U6, 5S, and RPPH1. A termination length of 6 was used for MRP. A termination length of 13 was used for U6ATAC. Data are represented as mean +/− SEM, and *P* values were determined by a two-sample two-tailed *t* test, with *P* < 0.05 shown in bold (*n* = 3 biological replicates for each transcript). (**D**) Percentage of Pol-III transcripts with post-transcriptional 3’-U tails in control or TENT2 KO conditions. Data are represented as mean +/− SEM, and *P* values were determined by a two-sample two-tailed *t* test, with *P* < 0.05 shown in bold (*n* = 3 biological replicates for each transcript). (**E**) Ratio of post-transcriptional U nucleotides preceded by either A or U nucleotides plotted for 3’-end positions +5 and +6 relative to the first nucleotide of the termination sequence (defined as +1) so as to ensure a post-transcriptional origin of the uridine. Data are represented as mean +/− SEM, and *P* values were determined by two-sample two-tailed *t* tests, with *P* < 0.05 shown in bold (*n* = 3 biological replicates for each transcript). “n.t.” denotes nucleotides, “3’ pos” denotes 3’ position. (**F**) Schematic representing uridine extension and trimming of newly transcribed Pol-III RNAs, which is terminated by monoadenylation by TENT2. Depiction of RNA was created in BioRender (MO27QXAFOF, https://biorender.com/j15w879).

Adenylation has previously been observed to inhibit uridylation of tested Pol-III RNAs in human cell extracts and when injected into nuclei of *Xenopus* oocytes (Chen et al, 2000). We therefore examined the impact of TENT2 on cellular Pol-III RNA uridylation and deuridylation dynamics. While U6 snRNA is known to undergo uridylation during biogenesis, many other Pol-III RNAs have instead been observed to undergo various levels of trimming of their genome-encoded uridine tails (Simons et al, 1996; Ciganda and Williams, 2011; Mroczek and Dziembowski, 2013; Yamashita and Tomita, 2023; Chen et al, 1998). Indeed, comparing Pol-III RNA 3’-end uridine termini in our 3’-end sequencing data for newly transcribed and steady state populations revealed that while U6 snRNA undergoes 3’-uridine extension during biogenesis, a majority of Pol-III RNAs experience significant trimming of 3’-uridines (Fig. 5A; Table EV3). To test whether mono(A)-tailing by TENT2 impacts Pol-III RNA 3’-end trimming, we used gene-specific sequencing to monitor the effect of TENT2 knockout on the mean number of 3’-uridines of select Pol-III RNAs. This revealed a general reduction in the number of 3’-end uridines of the tested Pol-III RNAs upon TENT2 KO, with the most highly adenylated Pol-III RNAs, 7SL1 and 7SL2 RNAs, showing significant shortening (Fig. 5B). Thus, monoadenylation by TENT2 stalls 3’-uridine trimming of 7SL RNAs and possibly other Pol-III RNAs.

Further analyses of our genome-wide sequencing data also revealed that all Pol-III RNA species see a fraction of their populations with post-transcriptional uridylation beyond their genome-encoded U-tails (Fig. 5C). For a subset of Pol-III RNAs, including 7SL1 and 7SL2 RNAs, this post-transcriptional uridylation was significantly more prevalent in the newly transcribed than the steady state population, suggesting that it is associated with biogenesis. The post-transcriptionally uridylated populations increased upon TENT2 knockout for each of the Pol-III RNA species monitored by gene-specific sequencing (Fig. 5D), suggesting that mono(A)-tailing inhibits post-transcriptional uridylation. Consistent with this idea, post-transcriptional uridines were preceded by uridines significantly more often than by adenosines for each of these RNAs (Fig. 5E). Taken together, these observations show that mono(A)-tailing by TENT2 inhibits both trimming and extension of Pol-III RNA 3’-uridine termini (Fig. 5F).

### TENT2 inhibits 7SL RNA association with La protein

**Figure EV5:**
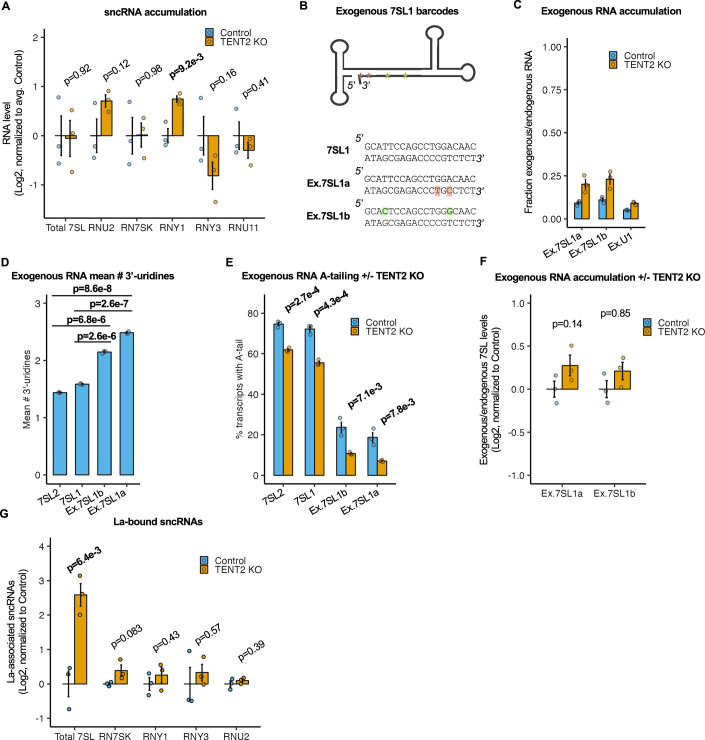
Exogenous 7SL1 RNAs accumulate with more 3’-uridines than endogenous 7SL RNAs. (**A**) Impact of TENT2 KO on sncRNA levels monitored by RT-qPCR. Levels were normalized against U1 snRNA. Data is represented as mean +/− SEM and significance between groups was determined by two-sample two-tailed *t* test with *P* < 0.05 in bold (*n* = 3 biological replicates for each condition). (**B**) A schematic representing the location of two point-mutations in exogenous 7SL1 RNAs *a* and *b* generated in order to distinguish exogenous from endogenous 7SL1 RNAs by sequencing. The 38 3’-terminal nucleotides of the endogenous and exogenous 7SL1 RNAs are shown below the schematic. (**C**) The abundance of exogenous 7SL1 and U1 RNAs relative to the endogenous 7SL1/2 and U1 RNAs, respectively, in control and TENT2 KO conditions monitored by gene-specific sequencing. Data is represented as mean +/- SEM (*n* = 3 biological replicates for each condition). (**D**) Mean number of 3’-uridines of endogenous and exogenous 7SL RNAs. Data is represented as mean +/− SEM and significance between groups was determined by two-sample two-tailed *t* test with *P* < 0.05 in bold (*n* = 3 biological replicates for each condition). (**E**) Percent A-tailing of endogenous and exogenous 7SL RNAs in control or TENT2 KO cells. Data is represented as mean +/− SEM and significance between groups was determined by two-sample two-tailed *t* test with *P* < 0.05 in bold (*n* = 3 biological replicates for each condition). (**F**) Levels of exogenous 7SL1 RNAs relative to endogenous 7SL RNAs in control or TENT2 KO cells, normalized to the relative levels of exogenous U1 versus endogenous U1 RNAs. Data is represented as mean +/− SEM and significance between groups was determined by two-sample two-tailed *t* test with *P* < 0.05 in bold (*n* = 3 biological replicates for each condition). (**G**) Levels of sncRNAs associated with La in control versus TENT2 KO conditions monitored by IP followed by RT-qPCR of sncRNAs relative to U1 snRNA and normalized against the IgG IP controls. Data is represented as mean +/− SEM and *P* value was determined by a two-sample two-tailed *t* test, with *P* < 0.05 indicated in bold (*n *= 3 biological replicates for each condition).

Given that 7SL1 and 7SL2 RNAs are the most highly adenylated sncRNAs with over 70% of the population accumulating with mono-A-tails, we next asked whether mono(A)-tailing by TENT2 impacts 7SL RNA biogenesis. We first asked if mono(A)-tailing by TENT2 affects 7SL RNA accumulation. Examining 7SL RNA levels in the presence or absence of TENT2 showed no effect of TENT2 depletion on 7SL RNA steady state accumulation (Fig. EV5A). Given that 7SL RNAs are highly stable molecules, we also monitored the impact of TENT2 on the accumulation of transiently expressed exogenous 7SL1 RNAs. The exogenous 7SL1 RNAs, for reasons that are unclear, are less adenylated and more uridylated at the 3’-end than endogenous 7SL RNAs and more dependent on TENT2 for their 3’-adenylation (Fig. EV5B–E). We therefore wondered whether the exogenous 7SL1 RNAs would be more dependent on TENT2 for their accumulation. However, TENT2 depletion did not impact the accumulation of exogenous 7SL1 RNAs as monitored by RNA sequencing (Fig. EV5F). These observations suggest that TENT2 does not significantly impact 7SL RNA stability.

**Figure 6:**
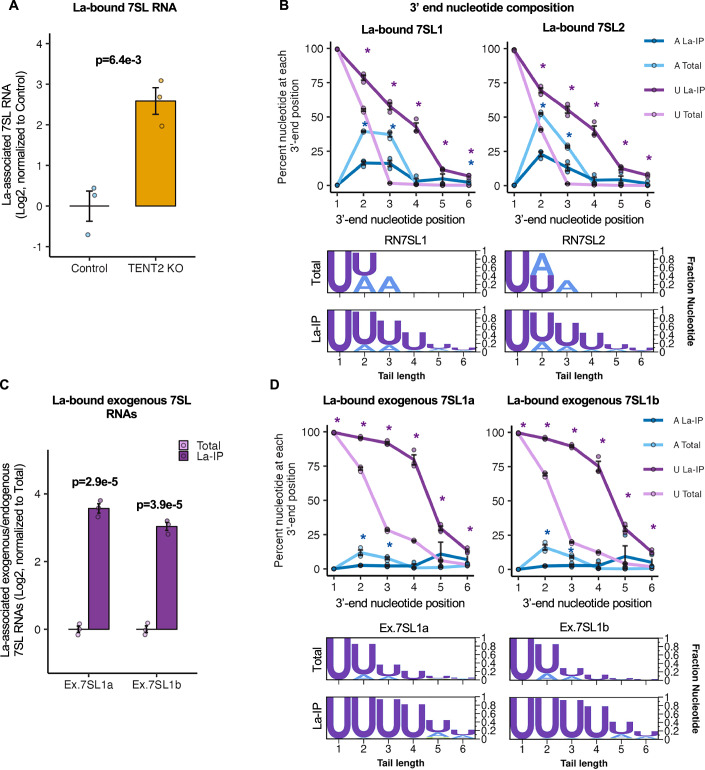
7SL RNA association with La protein is inhibited by TENT2. (**A**) Levels of 7SL RNAs associated with La in control versus TENT2 KO conditions monitored by IP followed by RT-qPCR for 7SL RNAs relative to U1 snRNAs and normalized against the IgG IP controls. Data are represented as mean +/− SEM, and *P* value was determined by a two-sample two-tailed *t* test, with *P* < 0.05 indicated in bold (*n* = 3 biological replicates for each condition). (**B**) 3’-end nucleotide compositions of steady state versus La-associated endogenous 7SL RNAs shown as line plots (top) and logo plots (bottom). Nucleotide positions are shown relative to the first nucleotide of the 7SL termination sequence, defined as position +1. Data are represented as mean +/− SEM, and *P* values between IP and input groups were determined by two-sample two-tailed *t* tests, with *P* < 0.05 indicated by asterisks (*n* = 3 biological replicates for each condition). Purple lines and asterisks represent U-tails, and blue lines/asterisks represent A-tails. (**C**) Levels of exogenous 7SL1a and 7SL1b RNAs associated with La relative to endogenous 7SL RNAs monitored by IP followed by sequencing. The relative abundance of exogenous 7SL1 RNAs bound to La was compared to the mean of endogenous 7SL1 and 7SL2 RNAs, then normalized to the relative abundance of exogenous U1 to endogenous U1. Data are represented as mean +/− SEM, and *P* values were determined by one-sample two-tailed *t* tests against mu = 0 with *P* < 0.05 indicated in bold (*n* = 3 biological replicates for each condition). (**D**) Same analysis as (**B**), but monitoring exogenous 7SL1 RNAs. “Ex.” denotes exogenous. Data are represented as mean +/− SEM, and *P* values between IP and input groups were determined by two-sample two-tailed *t* tests, with *P* < 0.05 indicated by asterisks (*n* = 3 biological replicates for each condition). Purple lines and asterisks represent U-tails, and blue lines/asterisks represent A-tails. Source data are available online for this figure.

Another possible impact of 7SL RNA monoadenylation could be on its association with RNA-binding proteins. The abundant nuclear RNA-binding protein La is known to have high affinity for RNAs with 3’-oligouridines (Stefano, 1984; Teplova et al, 2006; Rinke and Steitz, 1982) and has been previously observed to associate with 7SL RNA (Leung et al, 2014; Chambers et al, 1983). Performing immunoprecipitation against La and assessing associated 7SL RNA levels in the presence or absence of TENT2 revealed significantly increased 7SL RNA association with La in the absence of TENT2 (Fig. 6A). Consistent with the idea that adenylation by TENT2 inhibits La association, sequencing the 3’-ends of the La-associated 7SL RNAs revealed a significant enrichment in 3’ uridylated and de-enrichment in adenylated species as compared with the overall 7SL RNA population (Fig. 6B). Moreover, exogenous 7SL1 RNAs were enriched in association with La over the endogenous 7SL RNAs (Fig. 6C), consistent with their lower levels of adenylation and higher levels of uridylation (Figs. 6D and  EV5E). Other tested sncRNAs were not observed to be significantly enriched with La upon TENT2 depletion (Fig. EV5G), perhaps reflecting the smaller fractions of these RNA populations that receive mono(A)-tails by TENT2. These observations taken together demonstrate that mono(A)-tailing by TENT2 terminates 3’-uridine trimming at 7SL RNA 3’-ends and inhibits the association of 7SL RNAs with La protein, consistent with the known preference of La for RNAs with oligouridine 3’-termini. Moreover, given the nuclear localization of La, these observations suggest that mono(A)-tailing of 7SL RNA by TENT2 occurs in the nucleus. Consistent with this idea, TENT2 has been observed in the nuclei of vertebrate cells (Nakanishi et al, 2006; Rouhana et al, 2005) and is concentrated in the nuclear fraction of HEK293 cells (Fig. EV4E).

### Excessively uridylated 7SL RNAs are impaired in SRP54 assembly

**Figure 7:**
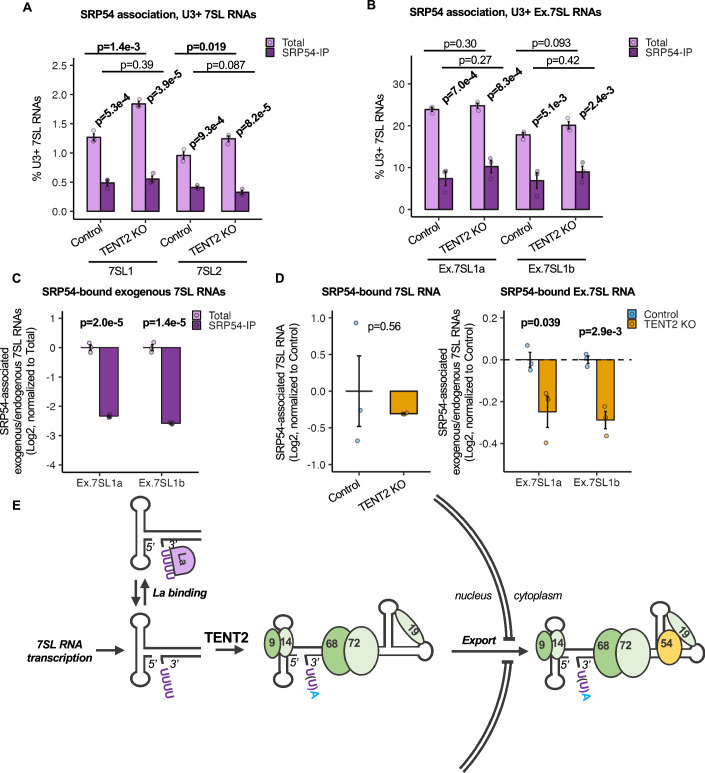
Excessively uridylated 7SL RNAs are impaired in the final step of SRP assembly. (**A**) Percentage of endogenous 7SL RNAs with 3 or more 3’-uridines in input versus SRP54-IP conditions and control versus TENT2 KO conditions. Data are represented as mean +/− SEM, and *P* values were determined by two-sample two-tailed *t* tests, with *P* < 0.05 indicated in bold (*n* = 3 biological replicates for each condition). (**B**) Same as (**A**) but monitoring exogenous 7SL RNAs. “Ex.” denotes exogenous. (**C**) Log2 ratio of SRP54-associated exogenous 7SL1 RNAs over endogenous 7SL RNAs, monitored by sequencing. Data are represented as mean +/− SEM, and *P* values were determined by one-sample two-tailed *t* tests against mu = 0 with *P* < 0.05 indicated in bold (*n* = 3 biological replicates for each condition). “Ex.” denotes exogenous. (**D**) Impact of TENT2 KO on 7SL RNA association with SRP54. Left: levels of SRP54-associated 7SL RNAs monitored by IP followed by RT-qPCR for 7SL RNAs relative to U1 snRNAs and normalized against the average level of association in the absence of TENT2 KO (control). Data are represented as the log2 mean +/− SEM, and *P* value was determined by a two-sample two-tailed *t* test, with *P* < 0.05 indicated in bold (*n* = 3 biological replicates for each condition). Right: log2 ratio of exogenous 7SL1 RNAs over endogenous 7SL RNAs in SRP54 IP samples in control and TENT2 KO conditions monitored by sequencing and normalized against the average association ratio in the absence of TENT2 KO (control). Data are represented as the log2 mean +/− SEM, and *P* values were determined by two-sample two-tailed *t* tests, with *P* < 0.05 indicated in bold (*n* = 3 biological replicates for each condition). “Ex.” denotes exogenous. (**E**) Schematic representing stages of 7SL biogenesis. 7SL RNAs with 3 or more uridines may be bound by La. TENT2 adenylates 7SL 3’-ends, which promotes release from La, allowing nuclear export and assembly of SRP in the cytoplasm with SRP54. Source data are available online for this figure.

La-associated RNAs have been reported to be retained in the nucleus (Simons et al, 1996; Stefano, 1984; Jacks et al, 2003; Boelens et al, 1995; Grimm et al, 1997). Given that the last step of SRP assembly, the association of 7SL RNA with SRP54, occurs in the cytoplasm, we considered the possibility that the association of 7SL RNA with La negatively impacts its subsequent nuclear export and assembly with SRP54 (Jacobson and Pederson, 1998; Ciufo and Brown, 2000; Grosshans et al, 2001). Given the preference of La for RNAs with three or more 3’-terminal uridines (Stefano, 1984; Teplova et al, 2006; Rinke and Steitz, 1982), we tested the prediction that 7SL RNAs with three or more 3’-end uridines should be de-enriched in their association with SRP54. Indeed, immunoprecipitation against SRP54 followed by 7SL RNA 3’ end sequencing revealed a significant de-enrichment of this population of molecules in association with SRP54 for both 7SL1 and 7SL2 RNAs (Fig. 7A). This could be observed both in the presence and absence of TENT2 KO. A similar de-enrichment in association with SRP54 was observed for exogenous 7SL1 RNAs with three or more 3’-uridines (Fig. 7B), and, consistent with their significantly higher levels of uridylation, exogenous 7SL1 RNAs showed significantly lower levels of association with SRP54 than endogenous 7SL RNAs (Fig. 7C). The overall level of endogenous 7SL RNA associated with SRP54 was not significantly impacted upon TENT2 KO (Fig. 7D) as may be expected given the low fraction (<2%) of endogenous 7SL RNAs that contain three or more 3’-uridines at steady state (Fig. 7A; Appendix Fig. S2A). However, the more extensively uridylated exogenous 7SL1 RNAs were de-enriched in association with SRP54 upon TENT2 KO (Fig. 7D; Appendix Fig. S2B). Taken together, these observations suggest that monoadenylation by TENT2 promotes 7SL RNA biogenesis by preventing the association of 7SL RNAs with La, which in turn allows 7SL RNAs to assemble with SRP54 in the cytoplasm to complete SRP biogenesis (Fig. 7E).

## Discussion

In this study, we globally characterized the dynamics of human sncRNA 3’-end processing and the relation between post-transcriptional tailing and sncRNA maturation and degradation. We observed post-transcriptional A-tailing of newly transcribed sncRNAs to be widespread and consist of two major types. One is characterized by transient oligo(A)-tailing, which was observed predominantly on newly transcribed snoRNAs and scaRNAs that were not fully processed at their 3’-ends (Figs. 1 and  EV2D). This oligo(A)-tailing is carried out by TENT4A/4B and is associated with instability rather than maturation (Fig. 2), consistent with the known link between TENT4A/4B and the nuclear exosome (Tseng et al, 2015; Shukla and Parker, 2017; Rammelt et al, 2011; Carneiro et al, 2007; Wlotzka et al, 2011). The second type of sncRNA A-tailing is characterized by mono(A)-tailing, which occurs on a majority of Pol-III RNAs and a smaller subset of snRNAs, and, in contrast to oligo(A)-tailing, stably accumulates to the steady state (Fig. 3). Mono(A)-tailing is broadly carried out by TENT2, although other polymerases appear to contribute as well (Fig. 4), and it inhibits Pol-III RNA 3’-uridine trimming and extension (Fig. 5). Mono(A)-tailing inhibits the interaction of 7SL RNAs with La protein (Fig. 6) and extensively uridylated 7SL RNAs, which are high-affinity La targets, are de-enriched from cytoplasmic SRP54 complexes, suggesting that mono(A)-tailing of 7SL RNAs promotes proper SRP biogenesis (Fig. 7).

Our observation of transient oligo(A)-tailing of snoRNAs that are not fully 3’-end processed is consistent with previous evidence for snoRNA adenylation by TENT4B occurring in competition with deadenylation by the deadenylase PARN during snoRNA maturation (2, 3, 27). We observed that transient oligo(A)-tailing of snoRNAs correlates with instability rather than maturation (Fig. 2E), which, together with the observation that adenylation occurs primarily on snoRNAs that are not fully 3’-end processed (Fig. 2F), suggests that adenylation by TENT4A/4B promotes degradation of snoRNAs whose maturation has stalled during 3’-end processing. We observed transient A-tailing and associated instability for H/ACA and C/D box snoRNAs and scaRNAs alike, suggesting that this mechanism of regulation is broadly applicable to these RNAs (Table EV2). These observations suggest that the competition between adenylation by TENT4A/4B and deadenylation by PARN (Son et al, 2018; Berndt et al, 2012) at snoRNA 3’-ends dictates whether these RNAs are processed to maturation or subjected to degradation. A similar mechanism has been observed to regulate telomerase RNA levels, whereby deadenylation by PARN protects TERT from degradation initiated by TENT4B-mediated oligoadenylation (Tseng et al, 2015). Similar oligoadenylation/deadenylation dynamics have additionally been observed for miRNAs (Jeong et al, 2023; Shukla et al, 2019) and snRNAs (Lardelli et al, 2017; Lardelli and Lykke-Andersen, 2020; Ma et al, 2024), suggesting that this is a widespread mechanism to control sncRNA expression.

The second type of post-transcriptional A-tailing that we observed consists of mono(A)-tailing, which we find is widespread among Pol-III RNAs and accumulates into the steady state. We identify TENT2 as broadly responsible for this monoadenylation event. Pol-III RNAs naturally terminate with 3’-uridines, and we observed that TENT2 inhibits the trimming of these U-tails that can be observed for a majority of Pol-III RNAs. Thus, mono(A)-tailing by TENT2 may serve to terminate 3’-end processing of Pol-III RNAs, reminiscent of how a 2’,3’ cyclic phosphate modification terminates processing of U6 and U6atac RNAs (Didychuk et al, 2017; Lund and Dahlberg, 1992).

Another consequence that we observed of mono(A)-tailing of Pol-III RNAs is inhibition of post-transcriptional uridylation. This is consistent with previous observations of adenylation of Pol-III RNAs inhibiting uridylation in human cell extracts and upon injection into *Xenopus* oocyte nuclei (Chen et al, 2000). The source of this uridylation is an important question for future study and may involve TUT1 in the nucleus and/or TUT4/7 in the cytoplasm. TENT2 has previously been observed to stabilize a variety of miRNAs in mice (D’Ambrogio et al, 2012; Katoh et al, 2009). Thus, it is possible that mono(A)-tailing likewise stabilizes Pol-III RNAs via inhibition of uridylation, a known trigger of RNA degradation in the cytoplasm (Belair et al, 2018; Łabno et al, 2016a). We did not observe evidence for decreased levels of Pol-III RNAs in the absence of TENT2. However, for the majority of Pol-III RNAs, mono(A)-tailing accumulates on less than half of the population, and it is therefore possible that the mono(A)-tailed population is insufficiently large to observe stabilization of the overall RNA species.

We observed 7SL RNAs as the most highly mono(A)-tailed RNAs, with over 70% of the population accumulating with mono(A)-tails at steady state. TENT2 is partly responsible for this adenylation, but our findings suggest that additional polymerase(s) are involved as well, the identity of which remains unclear; we observed no evidence for adenylation of 7SL RNAs by TENT4A/4B, TUT1, or PAPγ (Figs. EV3H and EV4A,B). Our observations that TENT2 depletion leads to significantly increased association of 7SL RNAs with La and that extensively uridylated 7SL RNAs are de-enriched from SRP54 complexes suggest that mono(A)-tailing by TENT2 promotes 7SL RNA biogenesis. Given evidence from others that La protein association results in nuclear retention (Simons et al, 1996; Stefano, 1984; Jacks et al, 2003; Boelens et al, 1995; Grimm et al, 1997), the simplest interpretation of our observations is that newly transcribed 7SL RNAs with unprocessed U-tails are retained in the nucleus by La, which prevents them from assembling into mature SRP particles in the cytoplasm. In this scenario, mono(A)-tailing by TENT2 would promote SRP assembly by inhibiting La association and allowing for nuclear export (Fig. 7E). Alternatively, extensively uridylated 7SL RNAs may undergo trimming or degradation prior to their association with SRP54 in the cytoplasm. An important question for future studies is what drives these 3’-uridine trimming and monoadenylation decisions, and whether these dynamics control the decision of whether Pol-III transcripts are destined for maturation or degradation.

## Methods

**Table:** Reagents and tools table

Reagent/resource	Reference or source	Identifier or catalog number
**Experimental models**		
HEK 293T-REx cells (*H. sapiens*)	Invitrogen	R71007
**Recombinant DNA**		
pcDNA6/TR	Invitrogen	V102520
pcDNA5/TO	Invitrogen	V652020
pUC19	Addgene	#50005
PX458	Addgene (Ran et al, 2013)	#48138
**Antibodies**		
rabbit polyclonal anti-TENT2	Thermo Fisher	PA5-65876
rabbit polyclonal anti-β-tubulin	Cell Signaling Technologies	2146
mouse monoclonal anti-GAPDH	Novus	NB300-221
rabbit monoclonal anti-LSD1	abcam	ab129195
donkey anti-rabbit HRP	Thermo Fisher	A16035
goat anti-mouse IRDye 800CW	LI-COR	LI-COR
mouse monoclonal anti-La	Santa Cruz Biotechnology	sc-80656
rabbit monoclonal anti-SRP54	Invitrogen	MA5-34835
Normal rabbit IgG	Cell Signaling Technology	2729
mouse IgG1	Cell Signaling Technology	5415
**Oligonucleotides and other sequence-based reagents**		
Primers, gRNAs, siRNAs	This study	Table EV1
**Chemicals, enzymes, and other reagents**		
Dulbecco’s Modified Eagle Medium	Gibco	11965118
Fetal Bovine Serum	Gibco	10437-028
penicillin/streptomycin	Gibco	15140122
TRIzol	Thermo Fisher	15596018
Sybr Gold stain	Thermo Fisher	S11494
Turbo DNA-free kit	Thermo Fisher	AM1907
FastAP	Thermo Fisher	EF0654
RNaseOUT	Thermo Fisher	10777019
Click-it nascent RNA capture kit	Thermo Fisher	C10365
ExoSAP-IT	Thermo Fisher	78200.200.UL
Superscript III	Thermo Fisher	18080085
SuperSignal West Femto substrate	Thermo Fisher	34096
Lipofectamine 2000	Thermo Fisher	11668019
Fast SYBR Green master mix	Thermo Fisher	4385612
Dynabeads Protein G	Thermo Fisher	10004D
BCA assay kit	Thermo Fisher	23225
Digitonin (5%)	Thermo Fisher	BN2006
RNA clean & concentrator	Zymo Research	R1014
RiboCOP rRNA depletion kit	Lexogen	144
PNK	New England Biolabs	M0201L
Q5 DNA polymerase	New England Biolabs	M0492L
T4 RNA ligase	New England Biolabs	B0216
Gibson assembly master mix	New England Biolabs	E2611S
DMSO	Sigma-Aldrich	D2650-5X5ML
Iodoacetamide	Sigma-Aldrich	I1149-5G
4-thiouridine	Sigma-Aldrich	T4509-100MG
AffinityScript Reverse Transcriptase	Agilent	600559
Tapestation D1000 Screentape	Agilent	5067-5582
D1000 Sample Buffer	Agilent	5067-5602
AMPure XP beads	Beckman Coulter	A63880
QuickExtract	Biosearch Technologies	QE09050
siLentFect reagent	Bio-Rad	703362
cOmplete protease inhibitor tablet	Roche	4693132001
**Software**		
Rstudio	Posit Software	http://www.posit.co/.
QuantStudio	Thermo Fisher	https://www.thermofisher.com/us/en/home/global/forms/life-science/quantstudio-6-7-flex-software.html
Tailer		https://github.com/TimNicholsonShaw/tailer
Tailer analysis		https://timnicholsonshaw.shinyapps.io/tailer-analysis/
**Other**		
Miseq	Illumina	
Novaseq 6000	Illumina	

### Mammalian cell culture

All cells were maintained in Dulbecco’s Modified Eagle Medium (DMEM, Gibco) supplemented with 10% Fetal Bovine Serum (FBS, Gibco) and 1% penicillin/streptomycin (Gibco) at 37 °C, 5% CO_2_. Mycoplasma testing was routinely performed.

### Global 3’-end sequencing of newly transcribed small RNAs

HEK293 T-REx cells were incubated with either 0.5 mM 5-ethynyluridine (EU; Thermo Fisher) or an equivalent volume of DMSO (*n* = 3 biological replicates per condition) for 2 h in order to accumulate sufficient molecules for detection by global sequencing and harvested in TRIzol reagent (Thermo Fisher). Total RNA was isolated according to the manufacturer’s recommendation. Small RNA from 180 μg of total RNA was isolated by separation in a 9% polyacrylamide/6 M urea denaturing gel. After Sybr Gold staining (Thermo Fisher), small RNAs ~90–500 nucleotides in length were excised and eluted with gel elution buffer (0.3 M sodium acetate pH 5.3, 1 mM EDTA, 0.1% SDS) by end-over-end rotation overnight at 4 °C. This size range was selected in order to avoid inclusion of mRNAs as well as tRNAs, the latter of which, due to their abundance and tendency to halt reverse transcription due to base modification, may negatively impact the depth of sequencing reactions. Eluted small RNAs were purified by RNA clean & concentrator columns (Zymo Research). Genomic DNA was removed using the Turbo DNA-free kit (Thermo Fisher) and ribosomal (r)RNAs were depleted using the RiboCOP rRNA depletion kit (Lexogen) per the manufacturer’s recommendations. RNA samples were treated with FastAP (Thermo Fisher) in 25 μl total volume and subsequently PNK (NEB) in 100 μl total volume in order to remove RNA 5’- and 3’-phosphates. RNA samples were again purified using RNA Clean & Concentrator columns (Zymo Research). AG15N or AG16N RNA adapters (1 μM, Table EV1) were ligated to the RNA 3’-ends by incubation at 25 °C for 90 min in a 40 µl reaction containing 9% DMSO (Sigma), ligase buffer (50 mM Tris-HCl pH 7.5, 10 mM MgCl_2_, 1 mM DTT), 1 mM ATP (Thermo Fisher), 16 units RNaseOUT (Thermo Fisher), 20% PEG 8000 (NEB), and 80 units T4 RNA ligase (NEB). RNA from ligation reactions was purified using RNA Clean & Concentrator columns (Zymo Research). In order to isolate newly transcribed RNAs labeled with EU, a portion of each sample was processed with Click-it nascent RNA capture kit (Thermo Fisher) as previously described (Lardelli and Lykke-Andersen, 2020), while the remaining portion did not undergo nascent RNA capture in order to represent the steady state population. To determine the effectiveness of the nascent enrichment step, two truncated exogenous β-globin RNA probes approximately 250 nucleotides in length were spiked in to samples prior to nascent extraction – one probe was in vitro transcribed with a 1:20 ratio of 5-ethynyl-UTP:UTP in order to mimic the estimated nucleotide ratio in cell culture, while a second probe was in vitro transcribed in the absence of 5-ethynyl-UTP (Table EV1). Reverse transcription of newly transcribed RNAs was performed on-bead in a total of 20 µl with 0.5 μM AR17 primer (Table EV1), 12.5 nM spike-in probe primer (Table EV1), AffinityScript buffer (1X, Agilent), 10 mM DTT, 4 mM dNTPs, 12 units RNaseOUT (Thermo Fisher), and AffinityScript Reverse Transcriptase (1X, Agilent) at 55 °C for 45 min, followed by 15 min incubation at 70 °C and 5 min of incubation at 85 °C to release cDNA. Reverse transcription of RNA representing steady-state accumulation was performed in tandem. Excess primer and RNA were removed from cDNA samples by incubating with 3.5 μl ExoSAP-IT (Thermo Fisher) at 37 °C for 15 min, then treated with 3 μl of 1 M NaOH at 70 °C for 12 min and subsequently neutralized with 3 μl of 1 M HCl. cDNA was extracted with phenol:chloroform:isoamyl alcohol and precipitated with 0.1 volume of 3 M sodium acetate pH 5.3, and 2.5 volumes of ethanol. A 3Tr3 adapter (Table EV1) was ligated to cDNA 3’-ends at a final concentration of 3.2 µM in a 20 μl reaction with 5% DMSO (Sigma), in ligase buffer (50 mM Tris-HCl pH 7.5, 10 mM MgCl_2_, 1 mM DTT), 1 mM ATP (Thermo Fisher), and 45 units T4 RNA ligase (NEB) at 25 °C for 16 h.

cDNA to be sequenced was amplified in two stages of Polymerase Chain Reaction (PCR) using Q5 DNA polymerase (NEB). For the first PCR reaction, the cDNA library was amplified using 3’-adapter primer (AR17) and a primer complementary to the 5’-adapter (RC_3Tr3) (Table EV1) for six cycles with an annealing temperature of 65 °C. The PCR product was purified by AMPure XP beads (Beckman Coulter) per the manufacturer’s recommendation. The second PCR reaction was performed using Illumina Truseq D50X and D70X primers (Table EV1) for six cycles of amplification with an annealing temperature of 68 °C, followed by two or nine additional cycles of amplification with an annealing temperature of 72 °C for steady state or newly transcribed RNA, respectively. The library quality was monitored by qPCR for select genes and Tapestation (Agilent) analyses. The relative ratios of EU-labeled to unlabeled spike-in probe cDNA were compared via qPCR in both newly transcribed samples and samples representing the steady state (Table EV1). In all, 100 bp paired-end sequencing was performed on an Illumina Novaseq S4.

### Sequencing data analyses

Fastq files were first subjected to 3’-adapter and PCR duplicate removal based on adapter randomers using custom Python scripts (https://pypi.org/project/jla-demultiplexer/). The residual Illumina adapter sequence was removed with Cutadapt (Martin, 2011). Reads were mapped to the human genome (version hg38) using STAR 2.7.11b (Dobin et al, 2013). A three-pass alignment strategy against a small RNA genome was used as previously described (Ma et al, 2024), but with a modification in order to improve the local alignment step, which provides post-transcriptional nucleotide modification information. After performing a three-pass end-to-end alignment, which includes a 5’-hard clip of 10 nucleotides to facilitate alignment of reads with post-transcriptional nucleotide modifications, the aligned reads were grouped by gene and re-converted into individual fastq files for each gene detected using bedtools (Quinlan and Hall, 2010) and samtools (Li et al, 2009). Individual-gene fastq files were then re-aligned with a three-pass local alignment to single-gene genomic sequences corresponding to the gene each file was previously aligned to in the end-to-end alignment. Gene-specific 3’-end information and graphs were subsequently generated using Tailer (Nicholson-Shaw and Lykke-Andersen, 2022) (https://github.com/TimNicholsonShaw/tailer) using the global alignment mode. RNAs that were full-length or extended up to 50 nucleotides were evaluated for 3’-end processing and modification, while RNAs truncated >10 nucleotides from the annotated 3’-end were removed from analyses. For post-transcriptional nucleotide tailing analyses, untemplated nucleotides were defined as only those nucleotides that do not match the reference genomic sequence. Any nucleotides which matched the reference genomic sequence were considered to be templated. Small noncoding RNA species with 1 or more reads in each of three biological replicates in the newly transcribed condition were evaluated in subsequent analyses. Analyses included all sncRNAs annotated in the GENCODE V33 annotation as gene_type snRNA, misc_RNA, rRNA, rRNA_pseudogene, snoRNA, scaRNA, ribozyme, and TERC RNA (Dataset EV1). tRNAs were not included in the analyses, and rRNAs were not further analyzed beyond initial mapping, given the selection against these molecules from the sequenced RNA samples.

### Gene-specific RNA 3’-end sequencing

RNA (*n* = 3 biological replicates per condition) was isolated using TRIzol (Thermo Fisher) per manufacturer’s recommendation. RNA was subsequently treated with DNase I (Zymo Research), purified with RNA Clean & Concentrator columns (Zymo Research), and AG15N or AG16N RNA adapters were ligated to RNA 3’-ends as described above. Following RNA purification with RNA Clean & Concentrator columns (Zymo Research), cDNA was synthesized with Superscript III (Thermo Fisher) using the AR17 primer (Table EV1). Gene-specific RNA 3’-end sequencing libraries were generated using gene-specific forward primers containing a unique barcode sequence for each sample (Table EV1) and the AR17 reverse primer (Table EV1) with Q5 polymerase (NEB) following six cycles of amplification at 50 °C annealing temperature, then 55 °C annealing temperature for 10–24 additional cycles depending on the target and abundance of material. Barcoded samples were pooled per gene target, and the PCR product was purified by AMPure XP beads (Beckman Coulter) per the manufacturer’s recommendation. A second PCR reaction was performed using Illumina Truseq D50X and D70X primers (Table EV1) for six cycles of amplification at 68 °C annealing temperature, then 72 °C annealing temperature for two additional cycles. Libraries were sequenced on an Illumina MiSeq platform and analyzed as previously described (Lardelli and Lykke-Andersen, 2020).

### CRISPR/Cas9 knockout cell lines

The human *Terminal Nucleotidyltransferase 2* (*TENT2)* gene was targeted with guide RNAs against PAM sites located at the 3’-end of exon 2 and 5’-end of intron 2 (Fig. EV3B; Dataset EV2). The guide RNA-containing constructs and Cas9 vector were prepared and transfected as described previously (Ran et al, 2013) into HEK293 T-REx cells. Cells were selected for transfection construct expression by fluorescence-activated cell sorting for GFP-positive fluorescence. GFP-positive cells were plated as single-cell colonies and evaluated for genomic deletion of *TENT2* sequence using primers flanking exon 2 and intron 2 (Table EV1). PCR products were cloned into the PX459 plasmid (Ran et al, 2013), transformed into DH5a *E. coli*, and prepared for Sanger and nanopore sequencing following plasmid purification (QIAprep, Qiagen). Six Sanger sequencing reactions and four nanopore sequencing reactions were performed for two individual knockout clones derived from isolated cell colonies. Three unique alleles were detected from clone A and two from clone B from ten and six successful sequencing reactions, respectively (Fig. EV3B; Dataset EV2). The loss of the exon 2 splicing junction is predicted to result in a premature stop codon. A loss of TENT2 protein expression for TENT2 KO clones but not the Control clone was validated by western blotting (Fig. EV3C). A control clone was derived from cells which were GFP-positive, but did not present evidence for TENT2 genomic deletion in alleles from four nanopore sequencing reactions (Dataset EV2) or the absence of TENT2 protein.

### Western blotting

Western blots were performed by separating proteins in SDS-polyacrylamide gels, followed by transfer to nitrocellulose membranes using standard procedures. Membranes were incubated overnight at 4 °C with primary antibody diluted 1:1000 (with the exception of anti-TENT2 antibody, which was used at 1:50–1:100) in TBS with 0.1% Tween 20 (TBST) and 5% milk powder. Secondary donkey anti-rabbit antibodies conjugated to HRP (Thermo Fisher) and secondary goat anti-mouse IRDye 800CW (LI-COR) were diluted 1:10,000 in TBST with 5% milk and incubated for two hours at room temperature. Protein was visualized with SuperSignal West Femto substrate (Thermo Fisher) using an Odyssey Fc imaging system (LI-COR).

### Generation of plasmid constructs and stable cell lines

Gibson assembly (New England Biolabs) was used to insert a cDNA copy of the human *TENT2* gene with a N-terminal-3XFLAG-tag sequence into the pcDNA5/FRT/TO (Thermo Fisher) plasmid, creating pcDNA5-3XFLAG-TENT2WT. The 3XFLAG-tag sequence alone was additionally inserted into pcDNA5/FRT/TO (Dataset EV2). Site-directed mutagenesis (New England Biolabs) was used to create a silent mutation in the coding sequence of *TENT2*, conferring siRNA resistance and creating pcDNA5-3XFLAG-TENT2WT-R (Dataset EV2). Site-directed mutagenesis was again used to mutate two codons (ATGGTGA > CTGGTGC), resulting in Asp to Ala mutations at residues 213 and 215 critical for nucleotidyltransferase catalytic activity of TENT proteins (Rissland et al, 2007), creating pcDNA5-3XFLAG-TENT2DADA-R (Dataset EV2). pcDNA5-3XFLAG, pcDNA5-3XFLAG-TENT2WT-R, and pcDNA5-3XFLAG-TENT2DADA-R constructs were subsequently integrated into the insertion site of Flp-In 293T-REx cells (Thermo Fisher) per the manufacturer’s recommendations in order to generate stable cell lines expressing 3XFLAG, 3XFLAG-TENT2WT-R, and 3XFLAG-TENT2DADA-R under control of a tetracycline-inducible promoter. Stable cell lines were treated with 50 ng/ml tetracycline 24 h prior to harvest in order to induce expression of 3XFLAG, 3XFLAG-TENT2WT-R, and 3XFLAG-TENT2DADA-R proteins.

To generate vectors for expression of exogenous 7SL1 RNA, genomic DNA from HeLa cells was isolated with QuickExtract (Biosearch Technologies Inc.) and the 7SL1 gene was amplified by PCR with Q5 polymerase (NEB), 3% DMSO, and primers positioned 154 base pairs upstream of the first transcribed nucleotide and 39 base pairs downstream of the annotated 3’-end (Table EV1). The amplicon was cloned into the pUC19 plasmid using standard molecular cloning techniques. The 7SL1 sequence was subsequently altered by Q5 (NEB) PCR amplification using primers (Table EV1) to achieve site-directed mutagenesis in order to create a distinguishable nucleotide barcode from endogenous 7SL1. Two barcoded exogenous 7SL1 sequences were synthesized (Table EV1).

### RNA interference and plasmid transfection

Two sequential knockdowns were performed using 40 nM of small interfering RNAs (siRNAs) custom ordered from Horizon Discovery (Table EV1), 72 h and 24 h prior to harvest. Knockdowns were performed with siLentFect reagent (Bio-Rad) according to the manufacturer’s specifications. The control siRNA targeted luciferase mRNA.

Transient transfection of a plasmid containing exogenous 7SL and U1 sequences was performed 18 h prior to harvest with Lipofectamine 2000 (Thermo Fisher) according to the manufacturer’s recommendations.

### qPCR assays

AR17 (Table EV1)-primed cDNA was amplified using Fast SYBR Green master mix (Thermo Fisher) containing ROX with 1 μM primers targeting sequences of the RNA of interest (Table EV1) in a 10 μL reaction. Technical duplicates were performed for each sample. Reactions underwent 40 cycles of 95 °C denaturation and 60 °C annealing with the QuantStudio Real-Time PCR system (Thermo Fisher) using the Fast protocol. Primer products were validated by quantification of primer efficiency, melt curve, and visualization by agarose gel electrophoresis. C_t_ values were determined with QuantStudio analysis software using default detection. Relative levels were quantified using the ∆∆C_t_ method (Livak and Schmittgen, 2001).

### Subcellular fractionation

Control or TENT2 KO 293T-REx cells were washed twice with ice-cold PBS on-plate, then scraped and transferred to microcentrifuge tubes in 1 mL PBS. Two million cells for each fractionation were subjected to centrifugation at 2000 rpm for 5 min at 4 °C, while 0.5 million cells were pelleted and reserved to represent protein levels for 25% of the input. Cells to be fractionated were resuspended in cytoplasmic lysis buffer (0.03% digitonin, 100 mM KCl, 25 mM HEPES-KOH pH 7.2, 15 mM Mg(OAc)_2_, 4 mM CaCl_2_, 500 U/mL RNaseOut, protease inhibitor tablet/10 mL (Roche)) and incubated for 1 min at 4 °C. The lysate was centrifuged at 6000 rpm for 5 min at 4 °C, and the supernatant was collected and saved as cytoplasmic material. The remaining pellet was washed with digitonin wash buffer (0.0015% digitonin, 100 mM KCl, 25 mM HEPES-KOH pH 7.2, 15 mM Mg(OAc)_2_, 4 mM CaCl_2_, 500 U/mL RNaseOut, protease inhibitor tablet/10 mL (Roche)) and centrifuged again at 6000 rpm for 5 min at 4 °C. The resulting pellet was saved as nuclear material.

### RNA immunoprecipitation

Control or TENT2 KO 293T-REx cells were transfected with plasmids expressing exogenous 7SL and U1 RNAs 18 h prior to harvest (*n* = 3 biological replicates per cell line). Cells were harvested by scraping into ice-cold PBS and flash frozen in liquid nitrogen. Cells were resuspended in ice-cold isotonic lysis buffer (50 mM Tris-HCl pH 7.5, 150 mM NaCl, 5 mM EDTA, 0.5% Triton X-100, 80 U/mL RNaseOut (Thermo Fisher), protease inhibitor tablet/10 mL (Roche)) and incubated with rotation for 20 min at 4 °C. Cellular debris was pelleted at 13,000× *g* for 15 min at 4 °C. The supernatant was briefly pre-cleared using Protein G beads (Thermo Fisher, 10004D) for 10 min at 4 °C with rotation. Beads for immunoprecipitation were washed with bead wash buffer (PBS pH 7.4, 0.1% Tween 20) and subsequently incubated with anti-La antibody (Santa Cruz Biotechnology), anti-SRP54 antibody (Invitrogen), rabbit IgG (Cell Signaling Technology), or mouse IgG1 (Cell Signaling Technology) antibody for 1 h at 4 °C with rotation, then washed with bead wash buffer. The sample protein was quantified with a BCA assay (Thermo Fisher). 1 mg of protein per sample was diluted fivefold with ice-cold detergent dilution buffer (isotonic lysis buffer without Triton X-100) and incubated with antibody-bead complexes for 1 h at 4 °C with rotation. Samples were washed five times with ice-cold immunoprecipitation wash buffer (PBS pH 7.4, 0.1% Tween 20, 150 mM NaCl) at 4 °C. RNA from input and immunoprecipitation material was extracted with TRIzol (Thermo Fisher) according to the manufacturer’s recommendations. Gene-specific 3’-end sequencing libraries were prepared as described above. Libraries were sequenced on an Illumina MiSeq platform.

### SLAM-seq

Control or TENT2 KO (*n* = 3 biological replicates per cell line) 293T-REx cell media was treated with 4-thiouridine (s4U) at a final concentration of 100 µM as previously described and incubated for 3 h, then refreshed with 100 µM s4U-containing media and incubated for an additional 3 h (Herzog et al, 2017). Cells were harvested by scraping into ice-cold PBS and flash frozen in liquid nitrogen. RNA was extracted with TRIzol (Thermo Fisher) according to the manufacturer’s recommendation. Following RNA extraction, samples were treated with either iodoacetamide (IAA) or dimethylsulfoxide (DMSO) for 15 min at 50 °C as previously described (Herzog et al, 2017). Gene-specific 3’-end sequencing libraries were prepared as described above. Libraries were sequenced on an Illumina MiSeq platform. Gene-specific alignments were performed using in-house scripts, which permitted T > C conversions. The number of conversions for each read was quantifie,d and reads with two or more conversions were subsequently analyzed as newly transcribed reads (Fig. EV3G).

### Study design

No blinding was performed in this study.

## Supplementary information

Appendix

Table EV1

Table EV2

Table EV3

Peer Review File

Dataset EV1

Dataset EV2

Dataset EV3

Dataset EV4

Source data Fig. 6

Source data Fig. 7

EV Figure Source Data

Expanded View Figures

## Data Availability

The datasets produced in this study are available in the following databases: Global 3’ end newly transcribed and steady state RNA sequencing data: Gene Expression Omnibus (GEO) (Barrett et al, 2013) accession GSE287260. Gene-specific 3’ end RNA sequencing data: GEO accession GSE287259. The source data of this paper are collected in the following database record: biostudies:S-SCDT-10_1038-S44318-025-00655-2.
