# Supplementary material for: Widespread mono- and oligoadenylation direct small noncoding RNA maturation versus degradation fates
Source: EMBO J. 2025 Dec 5;45(2):537–63. doi: 10.1038/s44318-025-00655-2 (PMC12811392; doi:10.1038/s44318-025-00655-2)
Supplement: Supplementary file 1 — Appendix [file 44318_2025_655_MOESM1_ESM.pdf]

Appendix

Widespread mono- and oligo-adenylation direct maturation versus degradation decisions during human small non-coding RNA biogenesis

Cody Ocheltree, Blake Skrable, Anastasia Pimentel, Timothy Nicholson-Shaw, Suzanne R. Lee and Jens Lykke-Andersen*

Correspondence authors: Jens Lykke-Andersen

Email: jlykkeandersen@ucsd.edu

Table of Contents:

**Title**	**Page**
Appendix Figure S1	2
Appendix Figure S2	3

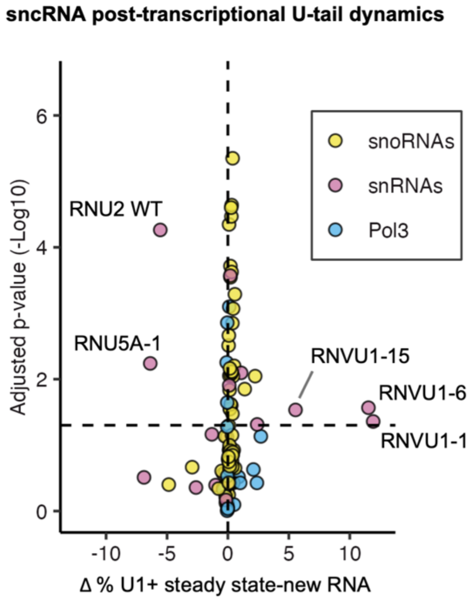

Appendix Figure S1: Change in newly transcribed and steady state sncRNA U tails. The difference in U-tails from steady state to newly transcribed conditions plotted against –Log10 p-values (n=3 biological replicates for each condition). The horizontal dashed line represents p=0.05.

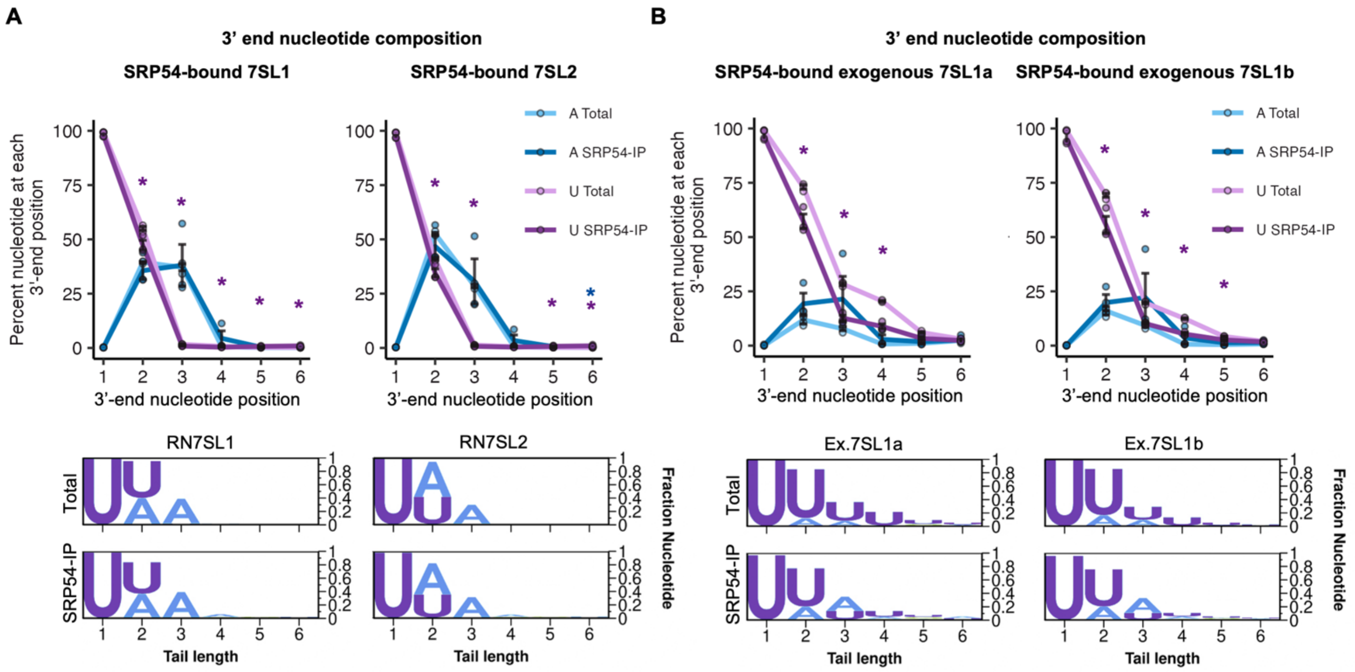

Appendix Figure S2: SRP54-associated 7SL RNAs are de-enriched for 3′-uridines. (A) 3' end nucleotide compositions of steady state versus SRP54-associated endogenous 7SL RNAs shown as line plots (top) and logo plots (bottom). Nucleotide positions are shown relative to the first nucleotide of the 7SL termination sequence, defined as position +1. Data is represented as mean +/- SEM and p-values between IP and input groups were determined by two-sample two-tailed t-tests, with $p<0.05$ indicated by asterisks (n=3 biological replicates for each condition). Purple lines and asterisks represent U-tails, and blue lines/asterisks represent A-tails. **(B)** Same analysis as panel *A*, but monitoring exogenous 7SL1 RNAs.
