# Supplementary figures and images for: Widespread mono- and oligoadenylation direct small noncoding RNA maturation versus degradation fates
Source: EMBO J. 2025 Dec 5;45(2):537–63. doi: 10.1038/s44318-025-00655-2 (PMC12811392; doi:10.1038/s44318-025-00655-2)

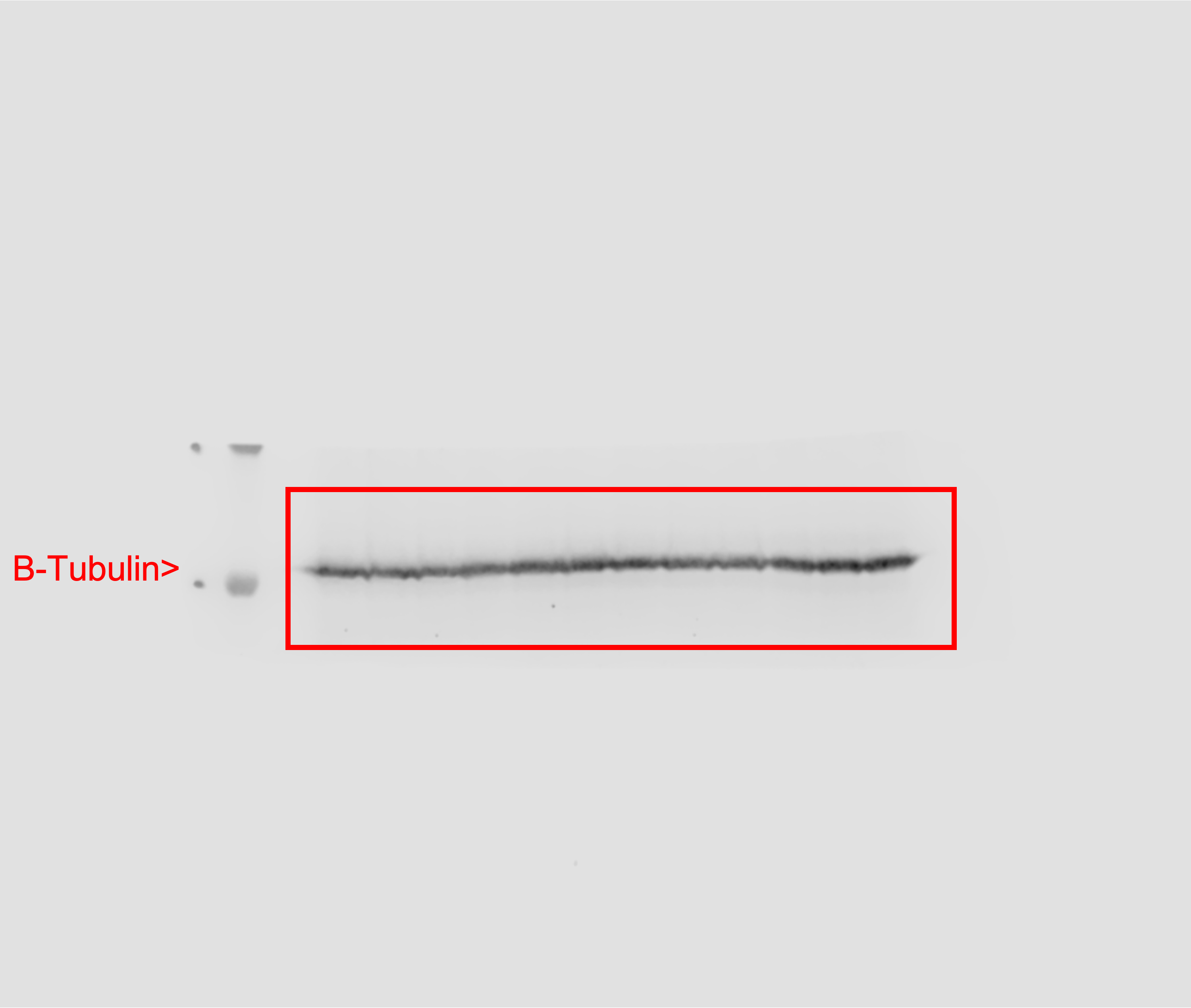

Supplement: Supplementary file 12 — EV Figure Source Data [file 44318_2025_655_MOESM12_ESM.zip › FigureEV3/FigureEV3E_western_btubulin.tiff]

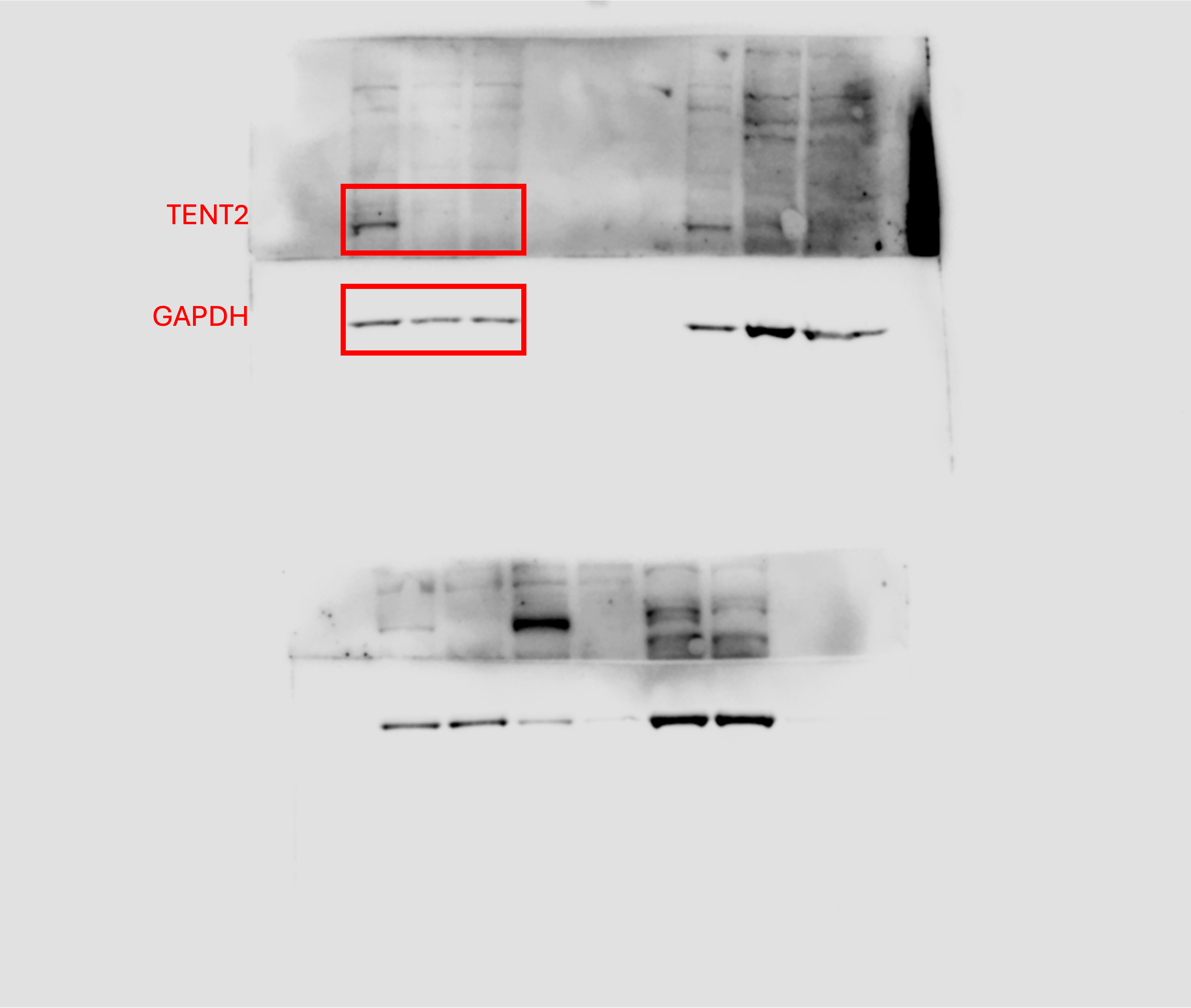

Supplement: Supplementary file 12 — EV Figure Source Data [file 44318_2025_655_MOESM12_ESM.zip › FigureEV3/FigureEV3C_western_tent2ko_gapdh.tiff]

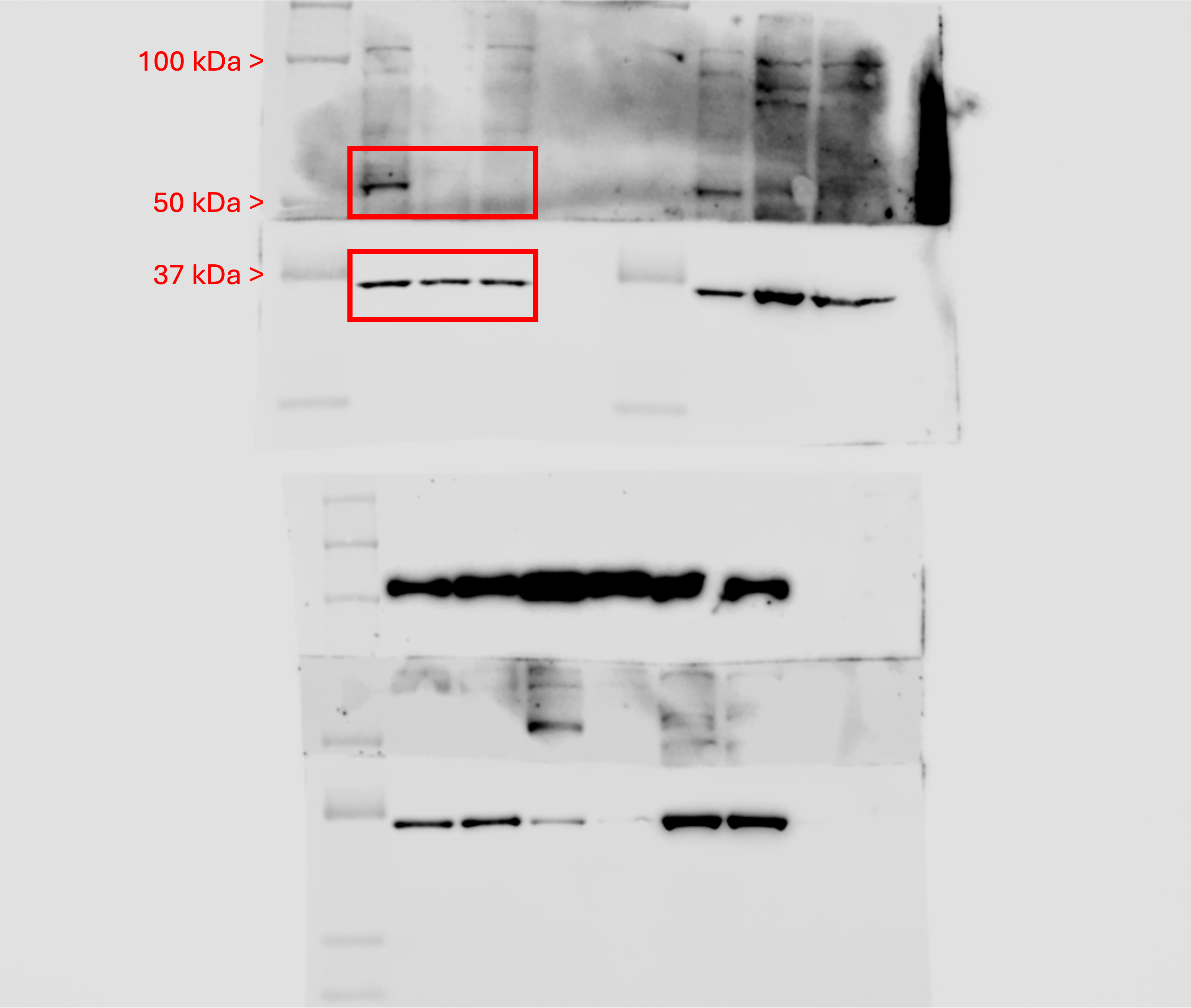

Supplement: Supplementary file 12 — EV Figure Source Data [file 44318_2025_655_MOESM12_ESM.zip › FigureEV3/FigureEV3C_western_tent2ko_gapdh_ladder.tiff]

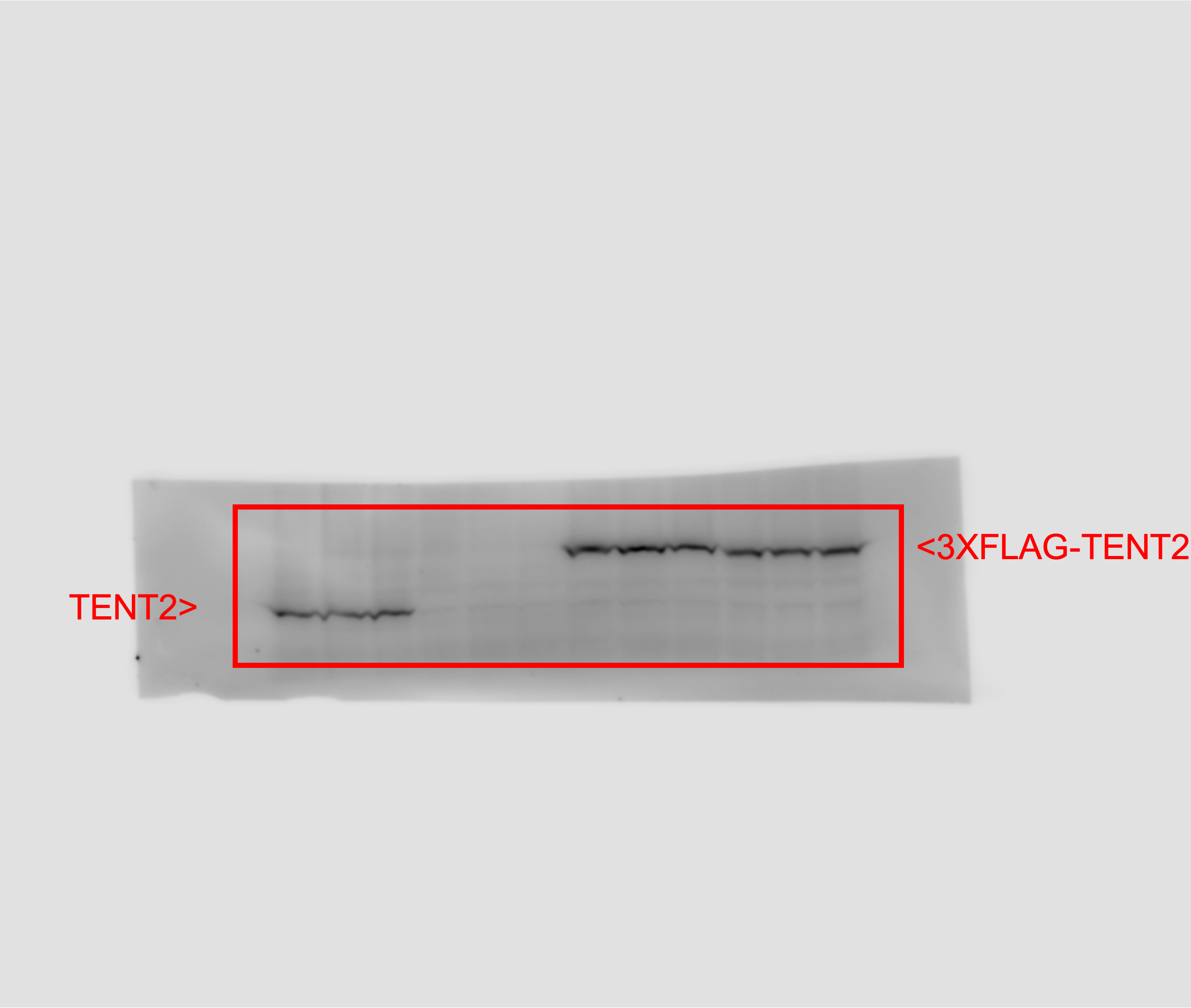

Supplement: Supplementary file 12 — EV Figure Source Data [file 44318_2025_655_MOESM12_ESM.zip › FigureEV3/FigureEV3E_western_tent2kd.tiff]

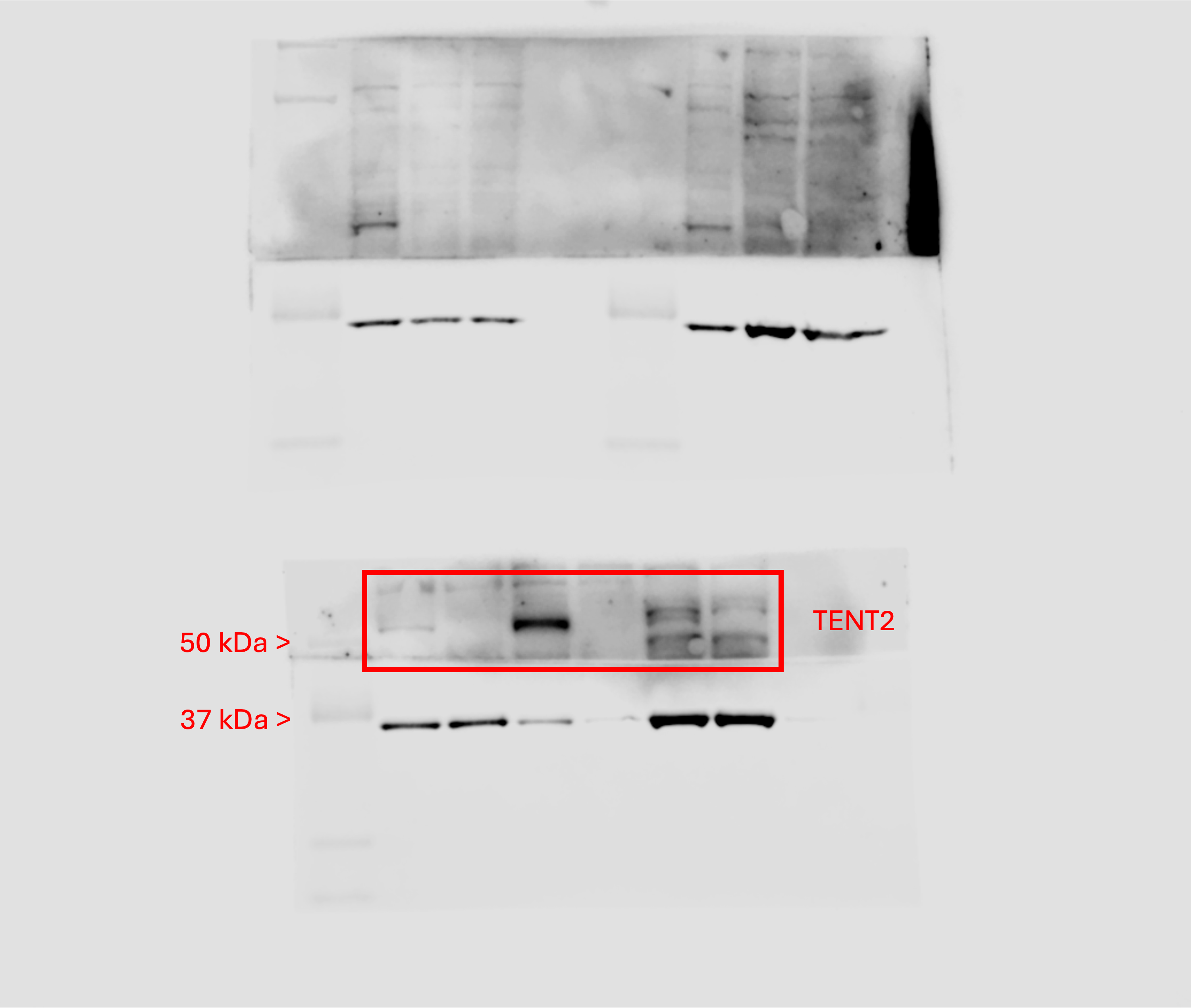

Supplement: Supplementary file 12 — EV Figure Source Data [file 44318_2025_655_MOESM12_ESM.zip › FigureEV4/FigureEV4E_western_tent2.tiff]

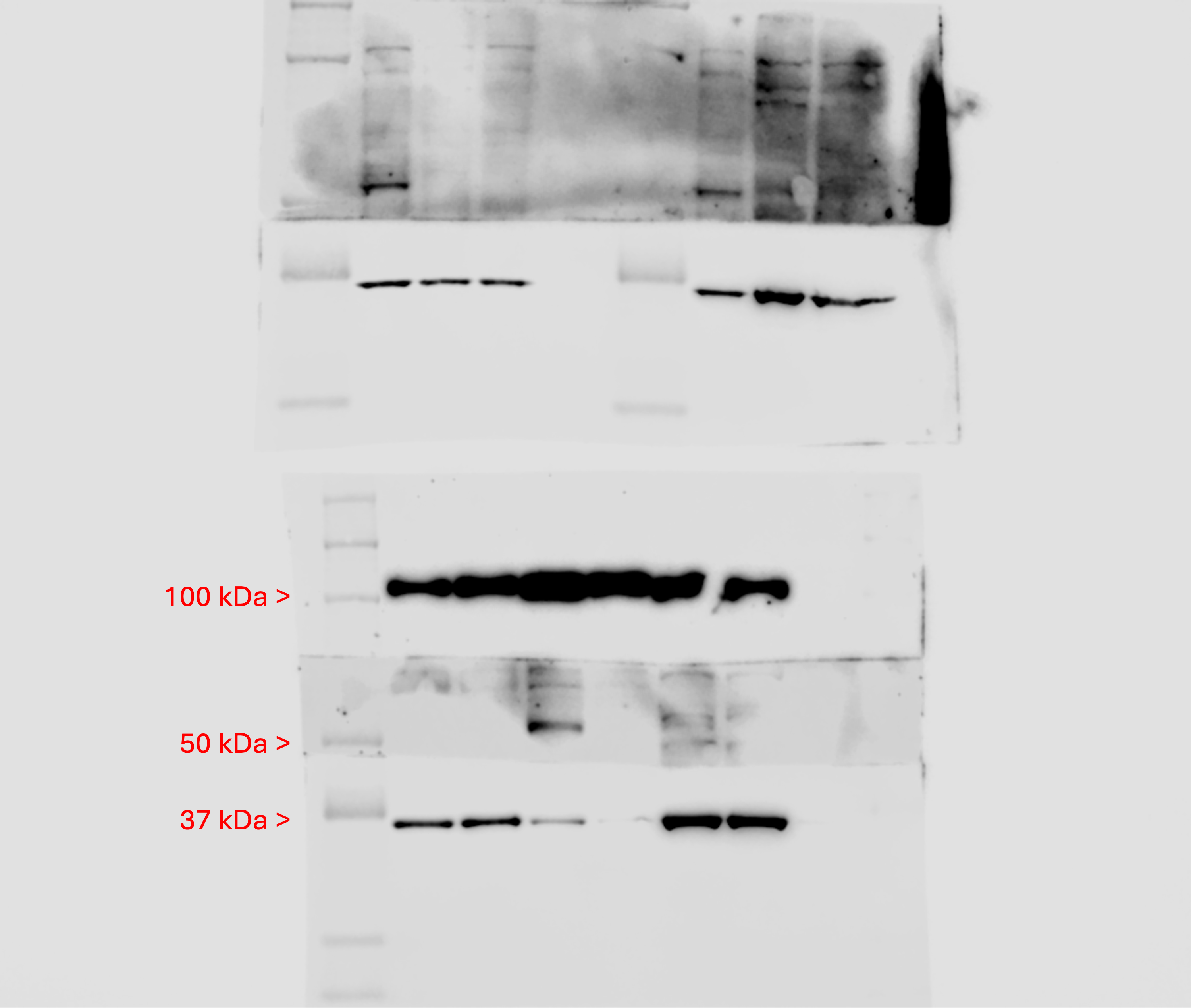

Supplement: Supplementary file 12 — EV Figure Source Data [file 44318_2025_655_MOESM12_ESM.zip › FigureEV4/FigureEV4E_western_ladder.tiff]

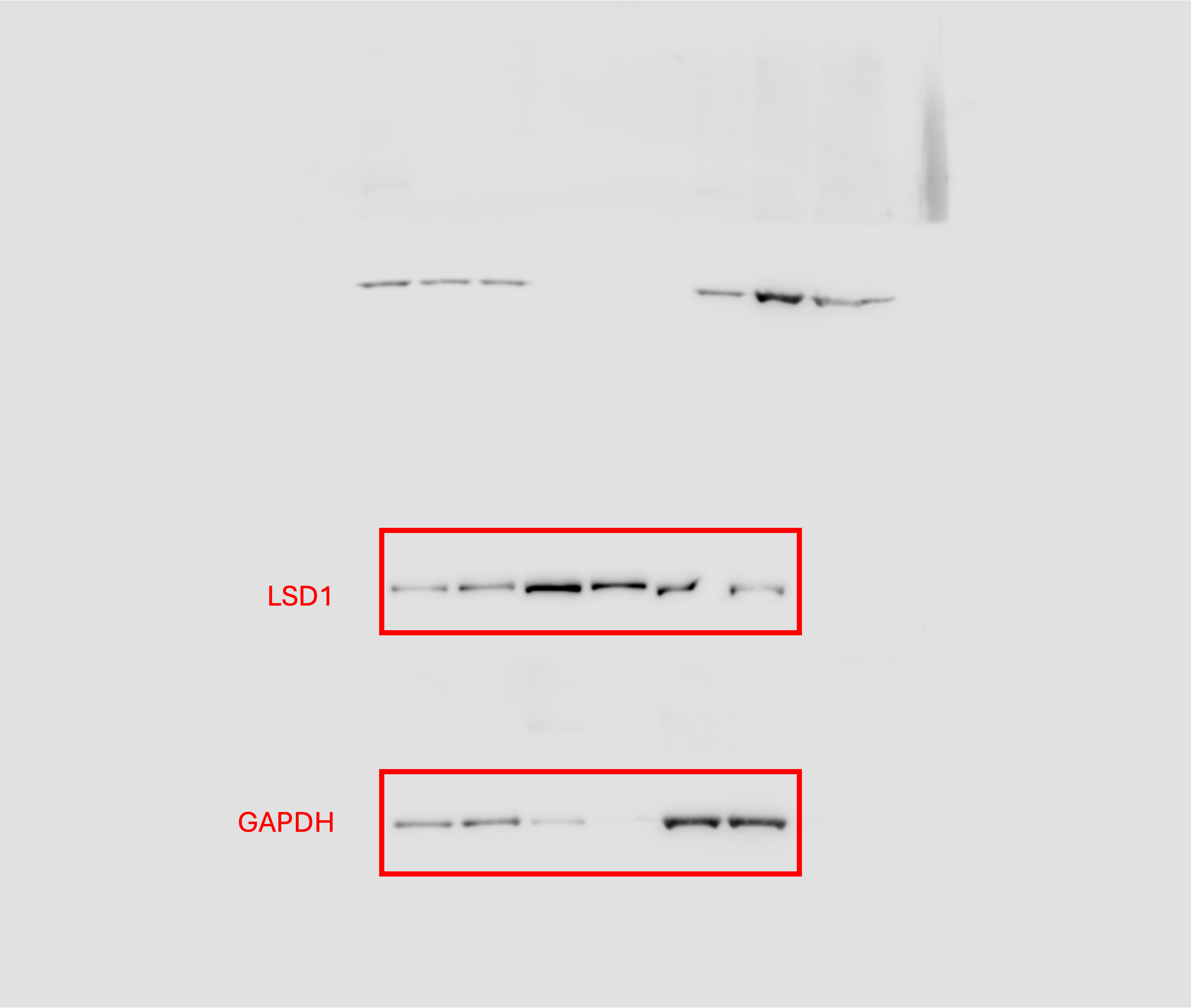

Supplement: Supplementary file 12 — EV Figure Source Data [file 44318_2025_655_MOESM12_ESM.zip › FigureEV4/FigureEV4E_western_lsd1_gapdh.tiff]
