# Supplementary material for: Widespread mono- and oligoadenylation direct small noncoding RNA maturation versus degradation fates
Source: EMBO J. 2025 Dec 5;45(2):537–63. doi: 10.1038/s44318-025-00655-2 (PMC12811392; doi:10.1038/s44318-025-00655-2)
Supplement: Supplementary file 13 — Expanded View Figures [file 44318_2025_655_MOESM13_ESM.pdf]

Expanded View Figures

Figure EV1. Global enrichment of newly transcribed sncRNAs.

(**A**) Efficiency of nascent sncRNA capture and size selection monitored by RT-qPCR for indicated RNAs from 2 h EU- relative to DMSO (0 h) treated samples. The library sample represents the total library monitored by RT-qPCR using primers against ligation linkers. Data is represented as mean +/− SEM and significance was determined by two-sample two-tailed *t* test with $P < 0.05$ shown in bold ($n = 3$ biological replicates per condition). (**B**) Efficiency of nascent RNA capture monitored by RT-qPCR for EU-labeled versus unlabeled spike-in β-globin probes. Data is represented as mean +/− SEM with and significance was determined by two-sample two-tailed *t* test with $P < 0.05$ shown in bold ($n = 3$ biological replicates per condition). (**C**) The mean proportion of reads aligned to each representative sncRNA class in newly transcribed and steady state libraries ($n = 3$ biological replicates for each condition). SNORA and SNORD: box H/ACA and box C/D snoRNAs, respectively. (**D**) The distribution of the 3′ end position for newly transcribed reads aligning to the U1 snRNA, 7SL1, and SNORA7A from an individual biological replicate. The terminal nucleotide is plotted by color as a post-transcriptionally-added A, U, C, or G nucleotide, or plotted in gray if the terminal nucleotide is encoded, as a fraction of total reads for each gene. (**E**) The percent of transcripts with homogenous post-transcriptional A-, U-, G- or C-tails in newly transcribed and steady state conditions ($n = 142$ transcripts per condition, $n = 3$ biological replicates for each condition). Box plots show the median (center), the interquartile range as bounds (25th to 75th percentile), and whiskers extending to the most extreme data point within 1.5× the interquartile range from the box, with outliers not shown. n.t. denotes nucleotide. (**F**) Data from panel *D* plotted as ranges of percentages of transcripts with homogenous post-transcriptional U-, G- and C-tails in newly transcribed and steady state conditions. Box plots show the median (center), the interquartile range as bounds (25th to 75th percentile), and whiskers extending to the most extreme data point within 1.5× the interquartile range from the box, with outliers not shown. n.t. denotes nucleotide. (**G**) Number of unique transcripts with 1% or greater homogenous post-transcriptional nucleotide tails in newly transcribed and steady state conditions. Data is represented as mean +/− SEM ($n = 3$ biological replicates for each condition). n.t. denotes nucleotide.

© The Author(s)

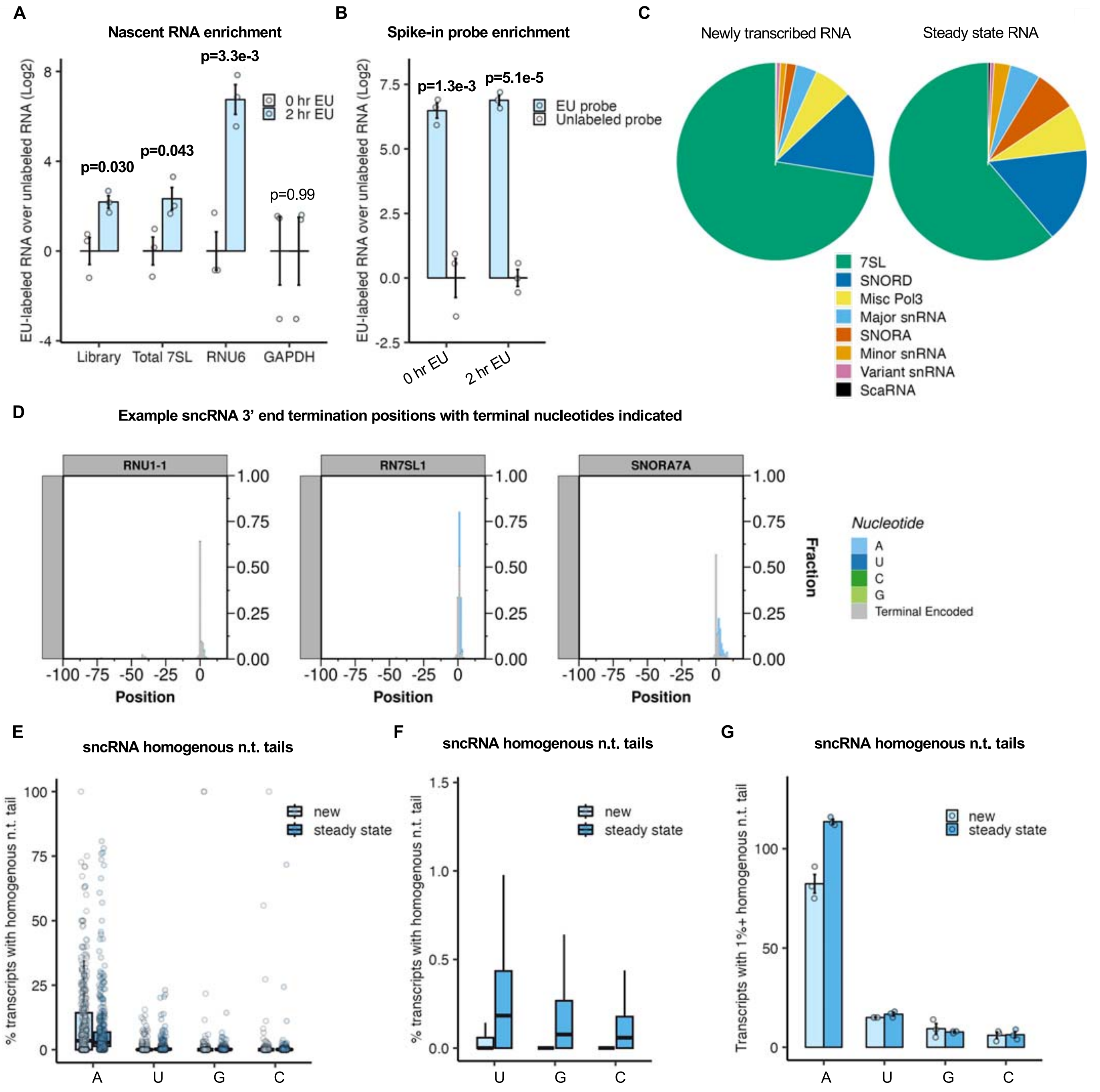
A
Nascent RNA enrichment
EU-labeled RNA over unlabeled RNA (Log2)
p=0.030
p=0.043
p=3.3e-3
p=0.99
0 hr EU
2 hr EU
Library
Total 7SL
RNU6
GAPDH
B
Spike-in probe enrichment
p=1.3e-3
p=5.1e-5
EU probe
Unlabeled probe
0 hr EU
2 hr EU
C
Newly transcribed RNA
Steady state RNA
7SL
SNORD
Misc Pol3
Major snRNA
SNORA
Minor snRNA
Variant snRNA
ScaRNA
D
Example sncRNA 3' end termination positions with terminal nucleotides indicated
RNU1-1
RN7SL1
SNORA7A
Position
Fraction
Nucleotide
A
U
C
G
Terminal Encoded
E
sncRNA homogenous n.t. tails
% transcripts with homogenous n.t. tail
new
steady state
F
sncRNA homogenous n.t. tails
G
sncRNA homogenous n.t. tails
Transcripts with 1%+ homogenous n.t. tail

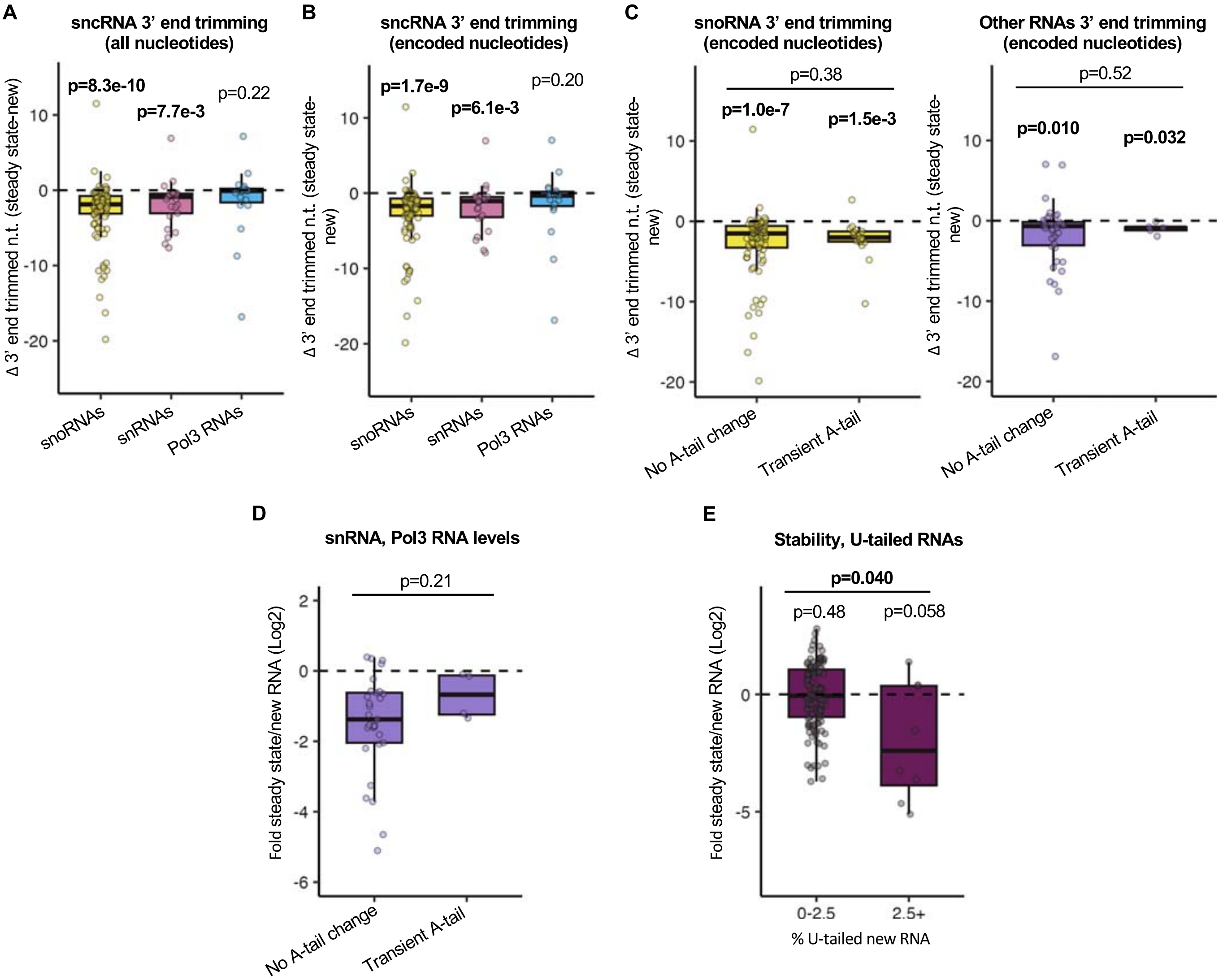

Figure EV2. 3′ end trimming and stability of tailed sncRNAs.

(**A**) Plot from Fig. 2A with individual transcripts visualized (snoRNAs $n = 98$, snRNAs $n = 25$, Pol3 RNAs $n = 19$). Box plots show the median (center), the interquartile range as bounds (25th to 75th percentile), and whiskers extending to the most extreme data point within 1.5× the interquartile range from the box, with outliers not shown. *P* values were determined by a one-sample two-tailed *t* test against mu = 0, with $P < 0.05$ shown in bold. "n.t." denotes nucleotides. (**B**) Plot from Fig. 2B with individual transcripts visualized (snoRNAs $n = 98$, snRNAs $n = 25$, Pol3 RNAs $n = 19$). Box plots show the median (center), the interquartile range as bounds (25th to 75th percentile), and whiskers extending to the most extreme data point within 1.5× the interquartile range from the box, with outliers not shown. *P* values were determined by a one-sample two-tailed *t* test against mu = 0, with $P < 0.05$ shown in bold. "n.t." denotes nucleotides. (**C**) Box plots showing 3′ end trimming of snoRNAs (left: no A-tail change $n = 80$ RNA species, transient A-tail $n = 18$ RNA species) and snRNAs/Pol-III RNAs (right: no A-tail change $n = 39$ RNA species, transient A-tail $n = 5$ RNA species) as measured by the difference in the mean 3′-end positions of transcripts in steady state versus newly transcribed RNA populations. RNAs that saw transient 3′ A-tailing, as measured by a significantly ($P < 0.05$; two-sample two-tailed *t* test) higher fraction of A-tailed molecules in newly transcribed over steady state populations, are compared to other RNAs. Box plots show the median (center), the interquartile range as bounds (25th to 75th percentile), and whiskers extending to the most extreme data point within 1.5× the interquartile range from the box, with outliers not shown. Significance for each group were determined by a one-sample two-tailed *t* test against mu = 0 with $P < 0.05$ in bold. Significance between groups was determined by a two-sample KS test with $P < 0.05$ in bold. "n.t." denotes nucleotides. (**D**) Box plots showing relative stabilities of snRNA/Pol-III RNAs as measured by log2 ratios of levels in steady state over newly transcribed conditions quantified using DESeq2. RNAs that saw transient 3′ A-tailing, as measured by a significantly ($P < 0.05$; two-sample two-tailed *t* test) higher fraction of A-tailed molecules in newly transcribed over steady state populations, are compared to other RNAs. Box plots show the median (center), the interquartile range as bounds (25th to 75th percentile), and whiskers extending to the most extreme data point within 1.5× the interquartile range from the box, with outliers not shown. Significance between groups was determined by a two-sample KS test with $P < 0.05$ in bold (no A-tail change $n = 29$ RNA species, transient A-tail $n = 4$ RNA species). (**E**) Same as panel *D* but binning sncRNAs by % U-tailing. Box plots show the median (center), the interquartile range as bounds (25th to 75th percentile), and whiskers extending to the most extreme data point within 1.5× the interquartile range from the box, with outliers not shown. Significance for each group were determined by a one-sample two-tailed *t* test against mu = 0 with $P < 0.05$ in bold. Significance between groups was determined by a two-sample KS test with $P < 0.05$ in bold (0-2.5% $n = 121$ RNA species, 2.5%+ $n = 8$ RNA species).

 © The Author(s)

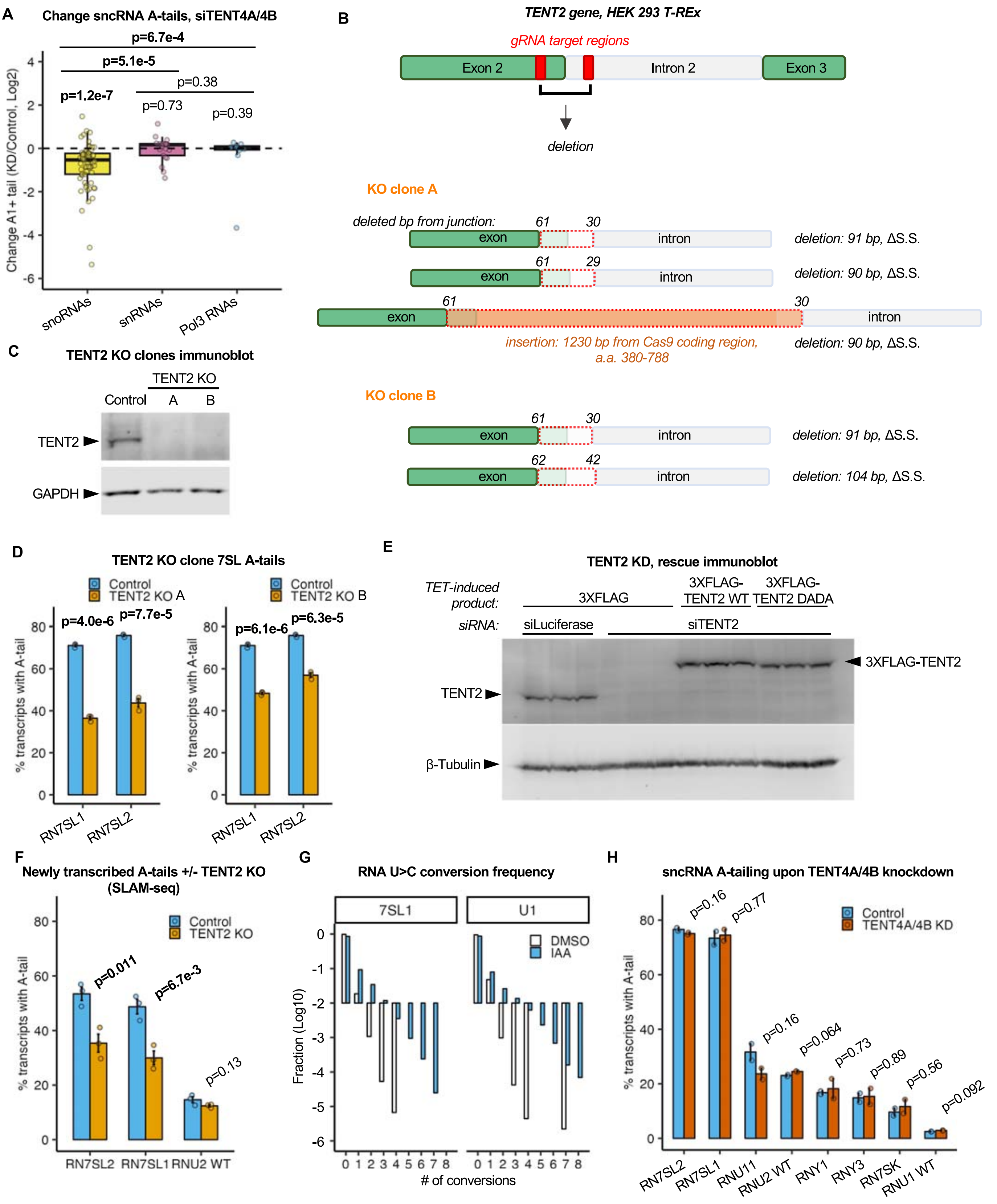
A
Change sncRNA A-tails, siTENT4A/4B
p=6.7e-4
p=5.1e-5
p=0.38
p=1.2e-7
p=0.73
p=0.39
Change A1+ tail (KD/Control, Log2)
snoRNAs
snRNAs
Pol3 RNAs
B
TENT2 gene, HEK 293 T-REx
gRNA target regions
Exon 2
Intron 2
Exon 3
deletion
KO clone A
deleted bp from junction:
exon
intron
deletion: 91 bp, ΔS.S.
deletion: 90 bp, ΔS.S.
insertion: 1230 bp from Cas9 coding region, a.a. 380-788
deletion: 90 bp, ΔS.S.
KO clone B
deletion: 91 bp, ΔS.S.
deletion: 104 bp, ΔS.S.
C
TENT2 KO clones immunoblot
TENT2 KO
Control A B
TENT2
GAPDH
D
TENT2 KO clone 7SL A-tails
Control
TENT2 KO A
TENT2 KO B
p=4.0e-6 p=7.7e-5
p=6.1e-6 p=6.3e-5
% transcripts with A-tail
RN7SL1
RN7SL2
E
TENT2 KD, rescue immunoblot
TET-induced product:
3XFLAG
3XFLAG-TENT2 WT
3XFLAG-TENT2 DADA
siRNA:
siLuciferase
siTENT2
3XFLAG-TENT2
TENT2
β-Tubulin
F
Newly transcribed A-tails +/- TENT2 KO (SLAM-seq)
Control
TENT2 KO
p=0.011
p=6.7e-3
p=0.13
RN7SL2 RN7SL1 RNU2 WT
G
RNA U>C conversion frequency
7SL1
U1
DMSO
IAA
Fraction (Log10)
# of conversions
H
sncRNA A-tailing upon TENT4A/4B knockdown
Control
TENT4A/4B KD
p=0.16
p=0.77
p=0.16
p=0.064
p=0.73
p=0.89
p=0.56
p=0.092
RN7SL2
RN7SL1
RNU11
RNU2 WT
RNY1
RNY3
RN7SK
RNU1 WT

Figure EV3. The impact of TENT2 and TENT4A/4B depletion on sncRNA A-tailing.

(**A**) Plot from Fig. 4A with individual transcripts visualized (snoRNAs $n = 67$, snRNAs $n = 21$, Pol3 RNAs $n = 10$). Box plots show the median (center), the interquartile range as bounds (25th to 75th percentile), and whiskers extending to the most extreme data point within 1.5× the interquartile range from the box, with outliers not shown. *P* values for individual groups were determined by one-sample two-tailed *t* tests against mu = 0. *P* values between groups were determined by two-sample KS tests. $P < 0.05$ is shown in bold. (**B**) Schematic of *TENT2* genomic region targeted by CRISPR-Cas9 guide (g)RNAs. *TENT2* gene alleles in KO clones A and B determined by PCR amplification of the surrounding region followed by Sanger and nanopore sequencing are illustrated; numbers above alleles refer to the position of the genomic deletion relative to the exon 2-intron2 5′ splice site. S.S. denotes splice site, bp denotes base pair, a.a. denotes amino acid. (**C**) Western blot for TENT2 in TENT2 KO clones A and B and a control clone that saw no TENT2 depletion. (**D**) Percent A-tailing of 7SL RNAs for two TENT2 KO clones. Data is represented as mean +/− SEM and significance was determined by two-sample two-tailed *t* test with $P < 0.05$ in bold ($n = 3$ biological replicates for each condition). Clone A was used in all subsequent analyses. (**E**) Western blot of TENT2 protein following siRNA knockdown in 293T-REx cells expressing exogenous 3XFLAG- or 3XFLAG-TENT2 in biological triplicate. 3XFLAG-TENT2 WT or catalytic mutant (DADA) were induced with tetracycline. β-tubulin is shown as a loading control. (**F**) Percent A-tailing of newly transcribed 7SL and U2 RNAs from control or TENT2 KO cells metabolically labeled with s4U. Reads containing 2 or more U > C conversions after IAA-treatment were considered newly transcribed. Data is represented as mean +/− SEM and significance was determined by two-sample two-tailed *t* test with $P < 0.05$ in bold ($n = 3$ biological replicates for each condition). (**G**) The percent of reads with 0 to 8 U > C conversions for 7SL1 and U1 RNAs after DMSO or IAA-treatment ($n = 1$ biological replicate for each condition). No bar represents none detected. (**H**) Percent A-tailing of select Pol-III/snRNAs during control or TENT4A/4B knock-down (KD) conditions ($n = 2$ biological replicates for each condition, data from Lim et al, 2018). Data is represented as mean +/− SEM. Source data are available online for this figure

© The Author(s)

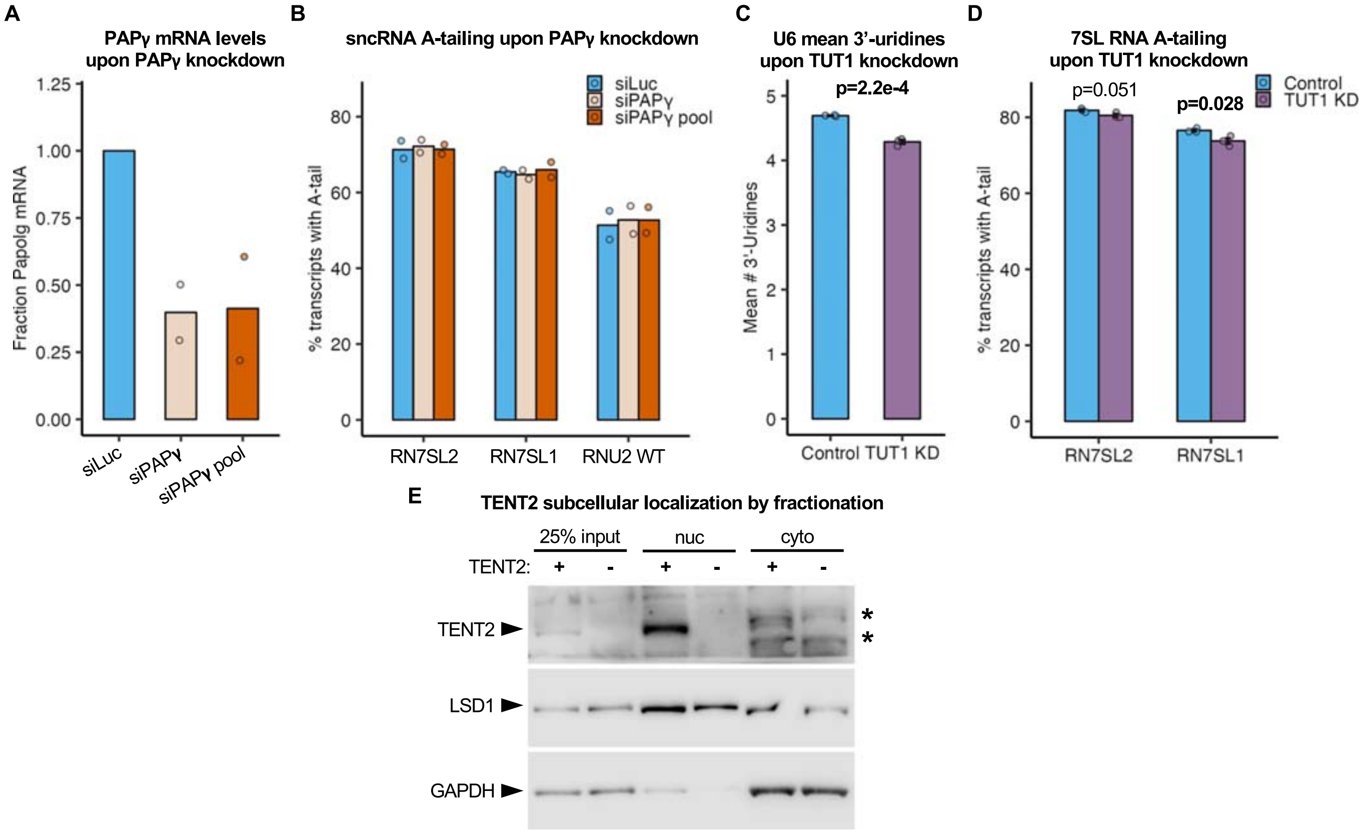

Figure EV4. Contributions of polymerases to sncRNA A-/U-tailing.

(**A**) Impact of PAPγ siRNA knockdown in HEK 293T-REx cells on Papolg mRNA levels monitored by RT-qPCR. A single siRNA against Papolg (siPAPg.1) was used in the siPAPγ condition, whereas two siRNAs against Papolg (siPAPg.1, siPAPg.2, sequences from Bresson 2013 (Bresson and Conrad, 2013), Table EV1) were pooled in the siPAPγ pool condition. Levels were normalized against U1 snRNA. Data is represented as mean ($n = 2$ biological replicates for each condition). (**B**) Percent A-tailing of select Pol-III/snRNAs during control or PAPγ knockdown conditions. Data is represented as mean ($n = 2$ biological replicates for each condition). (**C**) Impact of TUT1 siRNA knockdown in HEK 293T-REx cells on the mean number of 3′-uridines for the known target U6 snRNA as a functional validation of TUT1 knockdown. Data is represented as mean +/− SEM and significance between groups was determined by two-sample two-tailed *t* test with $P < 0.05$ in bold ($n = 3$ biological replicates for each condition). (**D**) Percent A-tailing of 7SL RNAs during control or TUT1 knockdown (KD) conditions. Data is represented as mean +/− SEM and significance between groups was determined by two-sample two-tailed *t* test with $P < 0.05$ in bold ($n = 3$ biological replicates for each condition). (**E**) Fractionation of Control (+) and TENT2 KO (−) cells into nuclear (nuc) and cytoplasmic (cyto) fractions. Input represents 25% of the fractionated material. LSD1 was used as a nucleoplasmic marker, while GAPDH was used as a cytoplasmic marker. Asterisks indicate non-specific bands produced by the TENT2 antibody. Source data are available online for this figure

© The Author(s)

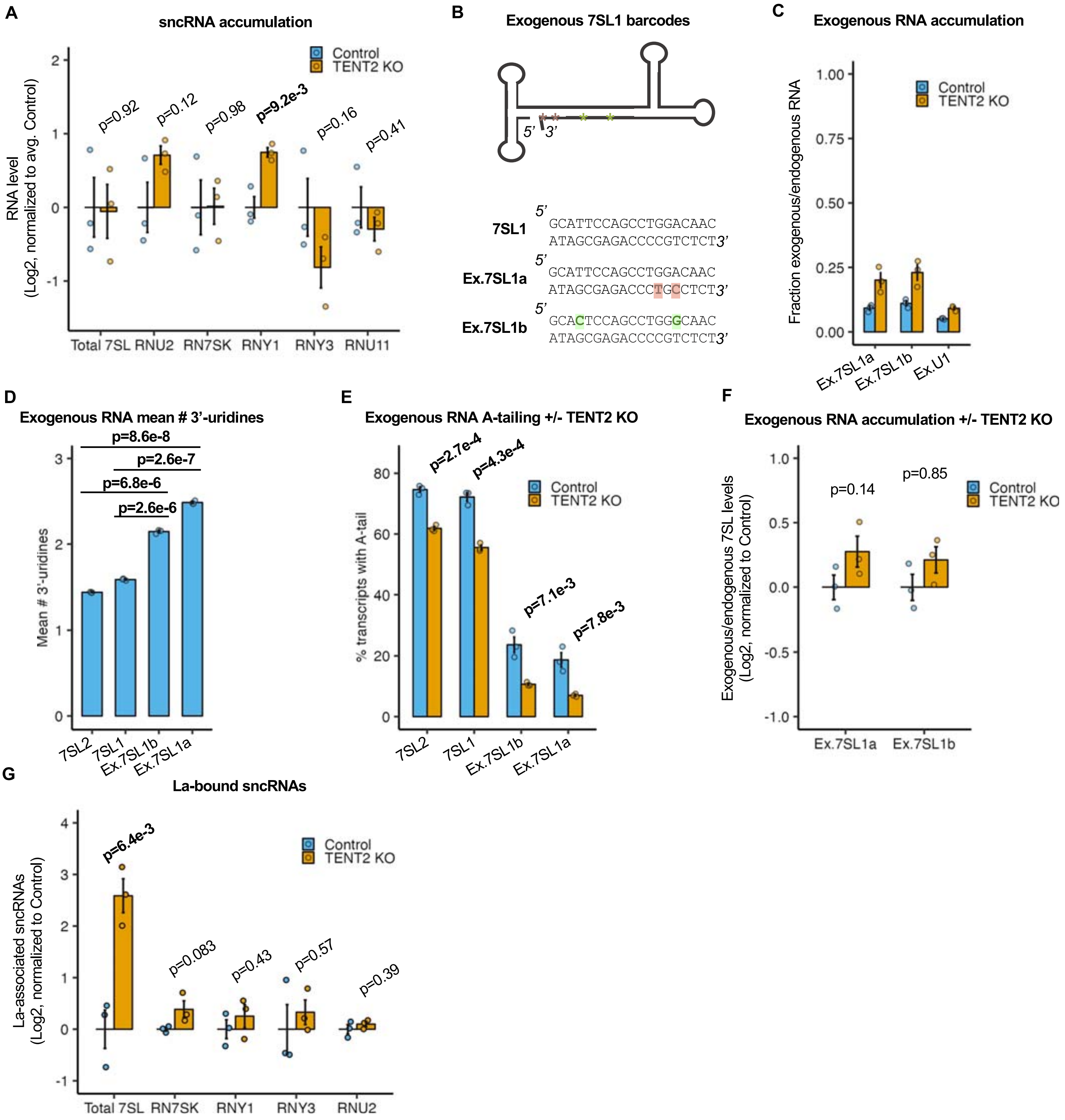
A
sncRNA accumulation
RNA level (Log2, normalized to avg. Control)
Control
TENT2 KO
p=0.92
p=0.12
p=0.98
p=9.2e-3
p=0.16
p=0.41
Total 7SL RNU2 RN7SK RNY1 RNY3 RNU11
B
Exogenous 7SL1 barcodes
7SL1
GCATTCCAGCCTGGACAAC
ATAGCGAGACCCCGTCTCT
Ex.7SL1a
GCATTCCAGCCTGGACAAC
ATAGCGAGACCCTGCCTCT
Ex.7SL1b
GCACTCCAGCCTGGGCAAC
ATAGCGAGACCCCGTCTCT
C
Exogenous RNA accumulation
Fraction exogenous/endogenous RNA
Ex.7SL1a Ex.7SL1b Ex.U1
D
Exogenous RNA mean # 3'-uridines
Mean # 3'-uridines
p=8.6e-8
p=2.6e-7
p=6.8e-6
p=2.6e-6
7SL2 7SL1 Ex.7SL1b Ex.7SL1a
E
Exogenous RNA A-tailing +/- TENT2 KO
% transcripts with A-tail
p=2.7e-4
p=4.3e-4
p=7.1e-3
p=7.8e-3
7SL2 7SL1 Ex.7SL1b Ex.7SL1a
F
Exogenous RNA accumulation +/- TENT2 KO
Exogenous/endogenous 7SL levels (Log2, normalized to Control)
p=0.14
p=0.85
Ex.7SL1a Ex.7SL1b
G
La-bound sncRNAs
La-associated sncRNAs (Log2, normalized to Control)
p=6.4e-3
p=0.083
p=0.43
p=0.57
p=0.39
Total 7SL RN7SK RNY1 RNY3 RNU2

 © The Author(s)

Figure EV5. Exogenous 7SL1 RNAs accumulate with more 3′-uridines than endogenous 7SL RNAs.

(**A**) Impact of TENT2 KO on sncRNA levels monitored by RT-qPCR. Levels were normalized against U1 snRNA. Data is represented as mean +/− SEM and significance between groups was determined by two-sample two-tailed *t* test with $P < 0.05$ in bold ($n = 3$ biological replicates for each condition). (**B**) A schematic representing the location of two point-mutations in exogenous 7SL1 RNAs *a* and *b* generated in order to distinguish exogenous from endogenous 7SL1 RNAs by sequencing. The 38 3′-terminal nucleotides of the endogenous and exogenous 7SL1 RNAs are shown below the schematic. (**C**) The abundance of exogenous 7SL1 and U1 RNAs relative to the endogenous 7SL1/2 and U1 RNAs, respectively, in control and TENT2 KO conditions monitored by gene-specific sequencing. Data is represented as mean +/- SEM ($n = 3$ biological replicates for each condition). (**D**) Mean number of 3′-uridines of endogenous and exogenous 7SL RNAs. Data is represented as mean +/− SEM and significance between groups was determined by two-sample two-tailed *t* test with $P < 0.05$ in bold ($n = 3$ biological replicates for each condition). (**E**) Percent A-tailing of endogenous and exogenous 7SL RNAs in control or TENT2 KO cells. Data is represented as mean +/− SEM and significance between groups was determined by two-sample two-tailed *t* test with $P < 0.05$ in bold ($n = 3$ biological replicates for each condition). (**F**) Levels of exogenous 7SL1 RNAs relative to endogenous 7SL RNAs in control or TENT2 KO cells, normalized to the relative levels of exogenous U1 versus endogenous U1 RNAs. Data is represented as mean +/− SEM and significance between groups was determined by two-sample two-tailed *t* test with $P < 0.05$ in bold ($n = 3$ biological replicates for each condition). (**G**) Levels of sncRNAs associated with La in control versus TENT2 KO conditions monitored by IP followed by RT-qPCR of sncRNAs relative to U1 snRNA and normalized against the IgG IP controls. Data is represented as mean +/− SEM and *P* value was determined by a two-sample two-tailed *t* test, with $P < 0.05$ indicated in bold ($n = 3$ biological replicates for each condition).

© The Author(s)
