# Supplementary material for: Widespread mono- and oligoadenylation direct small noncoding RNA maturation versus degradation fates
Source: EMBO J. 2025 Dec 5;45(2):537–63. doi: 10.1038/s44318-025-00655-2 (PMC12811392; doi:10.1038/s44318-025-00655-2)
Supplement: Supplementary file 5 — Peer Review File [file 44318_2025_655_MOESM5_ESM.pdf]

THE EMBO JOURNAL

Widespread mono- and oligoadenylation direct small noncoding RNA maturation versus degradation fates

Cody Ocheltree, Blake Skrable, Anastasia Pimentel, Timothy Nicholson-Shaw, Suzanne Lee, and Jens Lykke-Andersen

Corresponding author(s): Jens Lykke-Andersen (jlykkeandersen@ucsd.edu)

Review Timeline:

Submission Date:	10th Mar 25
Editorial Decision:	14th Jul 25
Revision Received:	4th Aug 25
Editorial Decision:	29th Sep 25
Revision Received:	6th Oct 25
Accepted:	5th Nov 25

Editor: Cornelius Schneider

Transaction Report:

(Note: With the exception of the correction of typographical or spelling errors that could be a source of ambiguity, letters and reports are not edited. Depending on transfer agreements, referee reports obtained elsewhere may or may not be included in this compilation. Referee reports are anonymous unless the Referee chooses to sign their reports.)

Dear Dr. Lykke-Andersen,

Thank you for submitting your manuscript for consideration by the EMBO Journal. It has now been seen by two referees whose comments are shown below. Thank you also for the productive consultation.
We think that the 3 proposed revision experiments are suitable to address the concerns raised by both referees. We recognize that the experiment required by ref #1 is technically challenging. We still think that it would be a valuable addition to the manuscript but it will not be a requirement for publication.

In summary I would like to invite you to submit a revised version of the manuscript, addressing the comments of both reviewers as described above. I should add that it is EMBO Journal policy to allow only a single round of revision, and acceptance of your manuscript will therefore depend on the completeness of your responses in this revised version.

When preparing your letter of response to the referees' comments, please bear in mind that this will form part of the Review Process File, and will therefore be available online to the community. For more details on our Transparent Editorial Process, please visit our website: https://www.embopress.org/page/journal/14602075/authorguide#transparentprocess

We generally allow three months as standard revision time. As a matter of policy, competing manuscripts published during this period will not negatively impact on our assessment of the conceptual advance presented by your study. However, we request that you contact the editor as soon as possible upon publication of any related work, to discuss how to proceed. Should you foresee a problem in meeting this three-month deadline, please let us know in advance and we may be able to grant an extension.

Thank you for the opportunity to consider your work for publication. I look forward to your revision.

Yours sincerely,

Cornelius Schneider, PhD
Editor
The EMBO Journal
c.schneider@embojournal.org

Instructions for preparing your revised manuscript:

Please make sure you upload a letter of response to the referees' comments together with the revised manuscript.

Please also check that the title and abstract of the manuscript are brief, yet explicit, even to non-specialists.

When assembling figures, please refer to our figure preparation guideline in order to ensure proper formatting and readability in print as well as on screen:
https://bit.ly/EMBOPressFigurePreparationGuideline
See also guidelines for figure legends: https://www.embopress.org/page/journal/14602075/authorguide#figureformat

At EMBO Press we ask authors to provide source data for the main manuscript figures. You will receive a separate email with instructions for providing source data with your revised manuscript, including how to upload and organize the files.

IMPORTANT: When you send the revision we will require
- a point-by-point response to the referees' comments, with a detailed description of the changes made (as a word file).
- a word file of the manuscript text.
- individual production quality figure files (one file per figure)
- a complete author checklist, which you can download from our author guidelines (https://www.embopress.org/page/journal/14602075/authorguide).
- Expanded View files (replacing Supplementary Information)
Please see out instructions to authors
https://www.embopress.org/page/journal/14602075/authorguide#expandedview
- a Reagents and Tools Table as part of the Methods section, which can be downloaded from our author guidelines (https://www.embopress.org/page/journal/14602075/authorguide#structuredmethods)

Please remember: Digital image enhancement is acceptable practice, as long as it accurately represents the original data and

conforms to community standards. If a figure has been subjected to significant electronic manipulation, this must be noted in the figure legend or in the 'Materials and Methods' section. The editors reserve the right to request original versions of figures and the original images that were used to assemble the figure.

Further information is available in our Guide For Authors: https://www.embopress.org/page/journal/14602075/authorguide

We realize that it is difficult to revise to a specific deadline. In the interest of protecting the conceptual advance provided by the work, we recommend a revision within 3 months (12th Oct 2025). Please discuss the revision progress ahead of this time with the editor if you require more time to complete the revisions.

Referee #1:

In this study, Ocheltree et al. set out to investigate the dynamics of 3' end processing of small ncRNAs with a focus on how different tails influence RNA maturation and/or stability. Using 3' end sequencing of steady state and recently transcribed RNAs, the authors uncover two distinct mechanisms that influence the fate of small ncRNAs: i) "transient" oligoadenylation, mediated by terminal nucleotidyltransferases TENT4A/B, which is interpreted as a destabilising element, and ii) stable monoadenylation tailing of some RNAPIII/II RNAs which can be, but is not exclusively, catalysed by TENT2 and acts as a stabilising element.

The manuscript is generally well written, and the data appear of good quality. Some of the interpretations and conclusions are overstated and broadly generalised even with clear caveats presented. Below are outlined major and minor points to be addressed before publication.

Major points;

- Two hours of EU incorporation does not only capture 'nascent' RNA, especially with the focus on short ncRNAs, that are typically highly transcribed (snRNAs, snoRNAs etc..). While the authors claim that this is to achieve sufficient enrichment over steady state ncRNAs which, for the RNAs investigated, are generally very stable; they should be clearer in the text about the caveats of this approach. 2h labelled RNAs will indeed be enriched for nascent RNAs, but the majority likely still represents fully-processed sncRNAs.
- The choice of which sncRNAs are included in the different analysis is not always clear. Some figures include numbers (e.g. n=74) but not all. Why do the numbers change between panels e.g. Fig 1D vs. 2A? Shouldn't the same RNAs be included in these analyses, and why are some excluded? Why are only sncRNAs with ≧5% of the population with A tails plotted in Fig 3A? The selection criteria should be rationalized. Besides, I am also missing the motivation or explanation as to which RNAs are included i.e. are all annotated snoRNAs/snRNAs included? Are tRNAs included in the RNAPIII group or is it just Y RNAs, 7SL, 7SK etc..? Are repetitive RNAs (e.g. SINEs) excluded? Why? This needs to be better described both in the results and the materials sections.
- The Fig. 3 results section is headlined 'A majority of Pol-III RNA species see subpopulations with stable mono-(A)-tailing' whereas the figure legend is headlined 'a subset of Pol-III RNAs and snRNAs stably accumulate with mono-A tails'. This is a bit confusing. Maybe it's not clear to me, but in Fig. 3A there are 2 populations of RNAPIII RNAs in the volcano plot and only 4 are past the significant threshold. The authors should be careful in being clear in their interpretation and description of these observations as the effects are sold as being general, but it's not clear how extensive this is.
- The data in Fig. 6 utilises an exogenously expressed 7SL1 RNA, which appears to behave differently from the endogenous 7SL RNAs. It is therefore tricky to make any conclusions using this data without first unravelling why the exogenous RNA behaves differently. Again, the authors should be clear and precise with their conclusions, claiming that 7SL RNA uridylation impairs SRP assembly. This is only observed for exogenous 7SL RNA (Fig. 7D right panel), but not endogenous 7SL RNA (Fig. 7D left panel). The model should clarify this. Could the difference result from the abundance of 7SL at steady state? If so, measuring nascent and endogenous 7SL binding of SRP54 might yield a similar result to exogenous 7SL.

Minor points;
- Some acryonyms are not definited in the introduction e.g. snoRNA, snRNA, scaRNA. It can be easy for non-experts to get lost in the nomenclature. Also - RNAPIII is defined "RNA Polymerase-III (Pol-III or Pol3)" but RNAPII does not get the same treatment earlier in the paragraph. Besides, what the 'n.t.' is short for on figure 1C should be define in the figure legend.
- It would be informative to have IGV screenshot examples of the sncRNAs with A-tails and U-tails in Fig. 1 or Fig. S1.
- The model in Fig 1E is not clear. Is the right handside 'steady state'? Why does the monoadenylation get larger from left to right? Does the upper panel indicate oligoadenylated RNA getting trimmed, or does the A tail disappear at steady-state because of degradation? These are two different scenarios, which should be discussed. Fig. 1E should be made more clear to convey the information.
- Some results sections would benefit from having a conclusion to wrap up the sections, e.g. for Figs 1+2.
- Does the TENT4A/B 3'-end RNA-seq dataset (ref 56) include small RNAs in the libraries? If not, it should be clearly stated if

the libraries exclude e.g RNAs < 200nt.
- Fig. 4C and 4D can be combined to better compare the rescue phenotype of the WT and the mutant.
- In Fig. 5, direct sequencing is used. Which sequencing technique is used here?
- Fig. 6 includes IPs of the La protein and isolation/sequencing of the associated RNA. It is shown that La-associated 7SL RNAs are more uridylated. As La is a polyU binding protein (U>3), this is not so surprising. I'm therefore not sure what this experiment adds.

Referee #2:

Regards EMBOJ-2025-120731
In the manuscript entitled "Widespread mono- and oligoadenylation direct small noncoding RNA maturation versus degradation fates" Cody Ocheltree and colleagues from the Jens Lykke-Andersen laboratory investigate non-templated 3' end tailing of small non-coding RNAs. They primarily isolate the RNAs following size-selection by denaturing RNA PAGE and excision of RNA species between 100 - 500 nucleotides in length. In comparison to microRNA and protein-coding mRNAs, RNAs of these sizes including snoRNA, snRNA, and RNA polymerase III transcripts have been less frequently analysed in high-throughput and in regards to their non-templated 3' end qualities. The authors differentiate between the nascent and steady-state RNA pools to tell about their 3' ends dynamics and fates imposed by them on the RNA. Furthermore, they identify known terminal nucleotidyl transferases responsible for the tailing. In a general view, the submitted manuscript represents a well performed study using state-of-the-art methodology. It is logically structured, concisely written. The figures are legible, properly referenced in the text, and captions adequate. The major question is in how far the findings presented in the paper meet the EMBO Journal expectations in terms of novelty, scope and outreach. In my opinion the findings will be relevant to researchers interested in RNA decay/ surveillance, and processing. However, the experimentation limits to a single model cell line. The manuscript benefits from the novel finding of the relevance of TENT2/GLD2 tailing to the RN7SL biogenesis. In this regard I find it critical that the authors present experimental confirmation of the nuclear localisation of TENT2 in the wild-type and rescued cells. Also experimental identification/ validation (PAP-γ) of the enzyme responsible for adenylation of RN7SL in TENT2 KO cells and assessing its involvement in the wild-type situation seems very important. Finally, data deposition (along with number of biological/ technical replicates) cannot be validated as no reviewer token has been provided in the data availability section.

Other minor points of concern (in order of appearance):
Page 4. Labno et al. 2016 PMID: 27431325 showed non-templeted 3' uridylation of sncRNA including vault and Y RNAs and its role in their decay by DIS3L2. The paper should be cited along Ustianenko et al., 2016 and Pirouz et al., 2016

Page 9. Methods. Please provide the number of independent biological/ technical replicates of the performed analyses. By principle more than 1 should have been performed for the knockout and recue studies. In "Gene-specific RNA 3' end sequencing" please be more specific about PCR conditions e.g. number of cycles. If more replicates were processed simultaneously were they pooled or treated separately?

Page 9. The authors are acknowledged for their thorough validation of the generated TENT2 knockout cells. The western blot provided has a weak band in the wild-type control cells and the blot is of unequal intensity (fading in the knockout lanes). I would advise to provide a blot with a strong signal in the wild-type, so that there are no concerns about the knockouts. Also a blot probed with αTENT2 antibodies across the entire size-separation would be great in the supplement.

Methods. Please be more detailed on how the differentiation between non-templated and template A/ U were made on the bioinformatics level. E.g. were pol III transcripts' terminator sequences always considered templated, and only extensions beyond the genome-encoded Ts (Us) considered non-templated?

Page 10. "Plasmid sequences are available upon request." Please include the sequences in the supplement.

Page 12. "containing ROX with 1 μM primers targeting RNAs of interest" rather "targeting sequences of the RNA of interest" as there is cDNA and not RNA in the qPCR reactions.

Page 16. "snoRNAs experienced significantly longer oligo(A) tailing (Figure 1C)." The authors should be clear in the text that the A-tailing they observe for snoRNAs is mostly 1-2 As. The significant difference in Figure 1C is an average of ~0,4A/transcript. Hence, I would generally resign from using "oligo(A)-tailing" in favour of "A-tailing".

Page 19. Maybe delete 'The non-canonical polymerase" in the paragraph heading and throughout?

Page 21. "we used direct sequencing to monitor the effect of TENT2 knockout" Maybe write "RNA species-specific". "Direct" could suggest direct RNA sequencing approach using Nanopore platform which is not the case.

Discussion. The authors concentrate on A-tailing (by investigating its consequences for RN7SL) but U-tailing is important in their study. I suggest they briefly discuss where in the cell does the uridylation they observe occur? In the nucleus or cytoplasm, what enzymes catalyse the uridylation, as the only known nuclear uridyltransferase is TENT1/ U6 TUTase? Given, the claims of U6

TUTase as adenyltransferase (star-PAP) is it possible it monoadenylates RNAs in the absence of TENT2?

The authors are requested to discuss possible ways of degradation of tailed sncRNAs. Specifically, if transient tailing is not significantly affecting 3' trimming of sncRNA then how are these unstable RNA removed? From the 5' end by XRN1/2, or does it occur instantly and cannot be seen in the data?

Response to reviewer comments (responses indicated in bullet points)

Referee #1

In this study, Ocheltree et al. set out to investigate the dynamics of 3' end processing of small ncRNAs with a focus on how different tails influence RNA maturation and/or stability. Using 3' end sequencing of steady state and recently transcribed RNAs, the authors uncover two distinct mechanisms that influence the fate of small ncRNAs: i) "transient" oligoadenylation, mediated by terminal nucleotidyltransferases TENT4A/B, which is interpreted as a destabilising element, and ii) stable monoadenylation tailing of some RNAPIII/II RNAs which can be, but is not exclusively, catalysed by TENT2 and acts as a stabilising element.

The manuscript is generally well written, and the data appear of good quality. Some of the interpretations and conclusions are overstated and broadly generalised even with clear caveats presented. Below are outlined major and minor points to be addressed before publication.

Major points;

- Two hours of EU incorporation does not only capture 'nascent' RNA, especially with the focus on short ncRNAs, that are typically highly transcribed (snRNAs, snoRNAs etc..). While the authors claim that this is to achieve sufficient enrichment over steady state ncRNAs which, for the RNAs investigated, are generally very stable; they should be clearer in the text about the caveats of this approach. 2h labelled RNAs will indeed be enriched for nascent RNAs, but the majority likely still represents fully-processed sncRNAs.

- *Two hours of EU incorporation was necessary for producing sufficient yield for sequencing and sufficient enrichment of newly transcribed RNAs over background (Supplementary Fig. S1 and unpublished titration assays). As the referee correctly points out this will be a population enriched for nascent RNAs as opposed to a clean nascent RNA population. For clarity, we now refer to this population as "newly transcribed" rather than "nascent" throughout the manuscript.*

- The choice of which sncRNAs are included in the different analysis is not always clear. Some figures include numbers (e.g. n=74) but not all. Why do the numbers change between panels e.g. Fig 1D vs. 2A? Shouldn't the same RNAs be included in these analyses, and why are some excluded? Why are only sncRNAs with ≧5% of the population with A tails plotted in Fig 3A? The selection criteria should be rationalized. Besides, I am also missing the motivation or explanation as to which RNAs are included i.e. are all annotated snoRNAs/snRNAs included? Are tRNAs included in the RNAPIII group or is it just Y RNAs, 7SL, 7SK etc..? Are repetitive RNAs (e.g. SINEs) excluded? Why? This needs to be better described both in the results and the materials sections.

- *The choice of which sncRNAs to include in different analyses were based on different thresholds needed for each analysis, which is now better clarified in each figure panel. For Fig 1D vs 2A, in Fig 1D only RNA species with 0.1% or greater tailing in both newly transcribed and steady state conditions were plotted to avoid division by 0, whereas in Fig 2A all RNAs (meeting read thresholds) were included since that analysis does not require division. For Fig. 3A, as adenylation was prevalent at sncRNA 3'-ends, RNA species with 5% or greater A-tailing in the steady state were included in order to evaluate RNAs with appreciable levels of accumulating A-tailing.*

- *With regards to which RNAs are included in the analyses this is now explicitly spelled out in the 'Sequencing data analyses' section in Materials & Methods and in the Figure 1 legend. Analyses included all sncRNAs annotated in the GENCODE V33 annotation as gene_type snRNA, misc_RNA, rRNA, rRNA_pseudogene, snoRNA, scaRNA, ribozyme, and TERC RNA. This includes all annotated canonical and variant snRNAs and sno/scaRNAs. For Pol-III RNAs, tRNAs were not included in the analyses and rRNAs were not further analyzed beyond initial mapping given the selection against these molecules from the sequenced RNA samples over concern of their impact on sequencing depth. SINE elements were not included in the analysis. All included genes are now listed in new Supplementary Table S2.*

- The Fig. 3 results section is headlined 'A majority of Pol-III RNA species see subpopulations with stable mono-(A)-tailing' whereas the figure legend is headlined 'a subset of Pol-III RNAs and snRNAs stably accumulate with mono-A tails'. This is a bit confusing. Maybe it's not clear to me, but in Fig. 3A there are 2 populations of RNAPIII RNAs in the volcano plot and only 4 are past the significant threshold. The authors should be careful in being clear in their interpretation and description of these observations as the effects are sold as being general, but it's not clear how extensive this is.

- *We have now modified the text for increased clarity. We changed the title of the Fig. 3 results section to say 'A majority of Pol-III RNA species see subpopulations with mono(A)-tails that accumulate to the steady state' and modified the second sentence of that section to state that Pol-III RNAs 'accumulate post-transcriptional A-tails at equal or higher levels at steady state than in the newly transcribed population' to better highlight the point that these A-tails accumulate in the steady state and are not removed (or degraded) during biogenesis. This population includes the four Pol3 RNAs referred to by the referee but also other Pol3 RNAs that do not see a reduction in A-tailing at steady state relative to the newly transcribed population.*

- The data in Fig. 6 utilises an exogenously expressed 7SL1 RNA, which appears to behave differently from the endogenous 7SL RNAs. It is therefore tricky to make any conclusions using this data without first unravelling why the exogenous RNA behaves differently. Again, the authors should be clear and precise with their conclusions, claiming that 7SL RNA uridylation impairs SRP assembly. This is only observed for exogenous 7SL RNA (Fig. 7D right panel), but not endogenous 7SL RNA (Fig. 7D left panel). The model should clarify this. Could the difference

result from the abundance of 7SL at steady state? If so, measuring nascent and endogenous 7SL binding of SRP54 might yield a similar result to exogenous 7SL.

- *The referee is correct that, for reasons we do not currently understand, exogenous 7SL1 RNAs see different relative levels of A- and U-tailing than endogenous 7SL RNAs, and therefore may not serve as perfect proxies for endogenous 7SL RNA. However, the referee is incorrect that 7SL RNA uridylation impairs SRP assembly only for exogenous 7SL RNA. In fact, all endogenous 7SL1, 7SL2, and exogenous 7SL1a and 7SL1b RNAs with U3+-tails are significantly ($p \leq 5.1e-3$) de-enriched in SRP54 complexes as compared to their overall levels as observed in Fig 7A for endogenous 7SL RNAs and in Fig 7B for the exogenous RNAs. The experiment proposed by the referee is an excellent suggestion for how one may test whether TENT2 depletion impairs assembly of nascent endogenous 7SL RNAs with SRP54, but this experiment is technically quite challenging and would require extensive optimization of nascent RNA labeling, detection, and IP conditions.*

Minor points;
- Some acryonyms are not definited in the introduction e.g. snoRNA, snRNA, scaRNA. It can be easy for non-experts to get lost in the nomenclature. Also - RNAPIII is defined "RNA Polymerase-III (Pol-III or Pol3)" but RNAPII does not get the same treatment earlier in the paragraph. Besides, what the 'n.t.' is short for on figure 1C should be define in the figure legend.

- *We have now defined these acronyms in the second paragraph of the Introduction section and the legend to Figure 1C.*

- It would be informative to have IGV screenshot examples of the sncRNAs with A-tails and U-tails in Fig. 1 or Fig. S1.

- *We now show a few examples in Supplementary Figure S1. Since the method used is 3' end sequencing the examples are shown as mapped 3' end positions.*

- The model in Fig 1E is not clear. Is the right handside 'steady state'? Why does the monoadenylation get larger from left to right? Does the upper panel indicate oligoadenylated RNA getting trimmed, or does the A tail disappear at steady-state because of degradation? These are two different scenarios, which should be discussed. Fig. 1E should be made more clear to convey the information.

- *We have now added additional labels to Figure 1E to clarify the schematic. The explanation for transient A-tailing of snoRNAs is explored in Figure 2.*

- Some results sections would benefit from having a conclusion to wrap up the sections, e.g. for Figs 1+2.

- *We have now added concluding sentences to the Results sections for Figures 1 and 2.*

- Does the TENT4A/B 3'-end RNA-seq dataset (ref 56) include small RNAs in the libraries? If not, it should be clearly stated if the libraries exclude e.g RNAs < 200nt.

- *These libraries underwent enrichment for RNAs >200 nts. This is now specified in the legend to Figure 4A.*

- Fig. 4C and 4D can be combined to better compare the rescue phenotype of the WT and the mutant.

- *Good suggestion. These graphs are now combined into a single Figure 4C, which better visualizes the impact of TENT2 complementation.*

- In Fig. 5, direct sequencing is used. Which sequencing technique is used here?

- *We used the term 'direct' incorrectly in the text; these are gene-specific 3' end sequencing-by-synthesis reactions, which has now been clarified.*

- Fig. 6 includes IPs of the La protein and isolation/sequencing of the associated RNA. It is shown that La-associated 7SL RNAs are more uridylated. As La is a polyU binding protein (U>3), this is not so surprising. I'm therefore not sure what this experiment adds.

- *Since TENT2 has been observed in the cytoplasm (see also response to referee 2 comments on TENT2 localization), this is an important experiment because TENT2 could have acted on 7SL RNAs in the cytoplasm rather than the nucleus, in which case TENT2 would be predicted to not impact 7SL RNA association with La protein. However, we observe a significant and dramatic increase in 7SL RNA association with La upon TENT2 KO. We added the following sentence at the end of the Figure 6 section of the Results to clarify the importance of this experiment: 'Moreover, given the nuclear localization of La, these observations suggest that mono(A)-tailing of 7SL RNA by TENT2 occurs in the nucleus.'*

Referee #2 (Report for Author)

Regards EMBOJ-2025-120731
In the manuscript entitled "Widespread mono- and oligoadenylation direct small noncoding RNA maturation versus degradation fates" Cody Ocheltree and colleagues from the Jens Lykke-Andersen laboratory investigate non-templated 3' end tailing of small non-coding RNAs. They primarily isolate the RNAs following size-selection by denaturing RNA PAGE and excision of RNA species between 100 - 500 nucleotides in length. In comparison to microRNA and protein-coding mRNAs, RNAs of these sizes including snoRNA, snRNA, and RNA polymerase III transcripts have been less frequently analysed in high-throughput and in regards to their non-

templated 3' end qualities. The authors differentiate between the nascent and steady-state RNA pools to tell about their 3' ends dynamics and fates imposed by them on the RNA. Furthermore, they identify known terminal nucleotidyl transferases responsible for the tailing. In a general view, the submitted manuscript represents a well performed study using state-of-the-art methodology. It is logically structured, concisely written. The figures are legible, properly referenced in the text, and captions adequate. The major question is in how far the findings presented in the paper meet the EMBO Journal expectations in terms of novelty, scope and outreach. In my opinion the findings will be relevant to researchers interested in RNA decay/ surveillance, and processing. However, the experimentation limits to a single model cell line. The manuscript benefits from the novel finding of the relevance of TENT2/GLD2 tailing to the RN7SL biogenesis. In this regard I find it critical that the authors present experimental confirmation of the nuclear localisation of TENT2 in the wild-type and rescued cells. Also experimental identification/ validation (PAP-γ) of the enzyme responsible for adenylation of RN7SL in TENT2 KO cells and assessing its involvement in the wild-type situation seems very important. Finally, data deposition (along with number of biological/ technical replicates) cannot be validated as no reviewer token has been provided in the data availability section.

- *Regarding TENT2 localization, we now provide a cell fractionation assay showing evidence for TENT2 enrichment in the nucleus (Supplementary Figure S5E), which is consistent with observations by others in vertebrate cells. We also tested three different TENT2 antibodies (Abcam ab103884, Invitrogen PA5-25015, Invitrogen PA5-65876) in immunofluorescence assays, but they all showed immunofluorescence signal that was indistinguishable between our TENT2 KO and control HEK293 cells.*

- *With regards to identification of enzyme(s) in addition to TENT2 responsible for adenylation of 7SL RNAs, we depleted candidate PAPγ and TUT1/STAR-PAP polymerases but saw little impact (<5%) on 7SL RNA adenylation in either case. These observations are now shown in Supplementary Figure S5A-D. Thus, the identification of additional enzyme(s) that contribute to 7SL RNA adenylation requires significant effort involving an in-depth screen, which we feel is beyond the scope of this manuscript.*

- *With regards to data deposition, all data had been deposited in GEO prior to initial submission and was fully available in connection with our deposition of the manuscript on BioRxiv (GEO accession: GSE287259 and GSE287260; i.e. no reviewer token was needed).*

Other minor points of concern (in order of appearance):
Page 4. Labno et al. 2016 PMID: 27431325 showed non-templeted 3' uridylation of sncRNA including vault and Y RNAs and its role in their decay by DIS3L2. The paper should be cited along Ustianenko et al., 2016 and Pirouz et al., 2016

- *We have now included this reference*

Page 9. Methods. Please provide the number of independent biological/ technical replicates of the performed analyses. By principle more than 1 should have been performed for the knockout and recue studies. In "Gene-specific RNA 3' end sequencing" please be more specific about PCR conditions e.g. number of cycles. If more replicates were processed simultaneously were they pooled or treated separately?

- *We have clarified the number of repeats for each experiment; the genome-wide newly transcribed and steady state sequencing assays were each performed in triplicate as were most other experiments. We have clarified PCR conditions in the Materials & Methods section.*

Page 9. The authors are acknowledged for their thorough validation of the generated TENT2 knockout cells. The western blot provided has a weak band in the wild-type control cells and the blot is of unequal intensity (fading in the knockout lanes). I would advise to provide a blot with a strong signal in the wild-type, so that there are no concerns about the knockouts. Also a blot probed with αTENT2 antibodies across the entire size-separation would be great in the supplement.

- *We have now provided an updated Western blot with stronger signal (Supplementary Figure S4C). Blotting for TENT2 with commercially available antibodies is difficult; as a full tube of antibody was needed to ensure sufficient signal for TENT2, the size-range below ≈45 kDa was separately probed for the internal GAPDH control, but we will upload the full TENT2 blot for the size range >45 kDa with the manuscript.*

Methods. Please be more detailed on how the differentiation between non-templated and template A/ U were made on the bioinformatics level. E.g. were pol III transcripts' terminator sequences always considered templated, and only extensions beyond the genome-encoded Ts (Us) considered non-templated?

- *We have now clarified in the Materials & Methods 'Sequencing data analyses' section that 'untemplated nucleotides were defined as only those nucleotides that do not match the reference genomic sequence'.*

Page 10. "Plasmid sequences are available upon request." Please include the sequences in the supplement.

- *We have now updated Supplementary Table S3 to include the sequences of tetracycline-inducible 3XFLAG and 3XFLAG-TENT2 coding regions, in addition to the previously-available 7SL1a and 7SL1b expression sequences.*

Page 12. "containing ROX with 1 µM primers targeting RNAs of interest" rather "targeting sequences of the RNA of interest" as there is cDNA and not RNA in the qPCR reactions.

- *We have made this correction*

Page 16. "snoRNAs experienced significantly longer oligo(A) tailing (Figure 1C)." The authors should be clear in the text that the A-tailing they observe for snoRNAs is mostly 1-2 As. The significant difference in Figure 1C is an average of ~0,4A/transcript. Hence, I would generally resign from using "oligo(A)-tailing" in favour of "A-tailing".

- *We believe the term oligo(A)-tailing helps readability of the manuscript as a better contrast to mono(A)-tailing than A-tailing would be, and we believe the term is justified since we show that these tails are appended by TENT4A/B, known to produce oligo(A)-tails. The mean length of these tails in cells is impacted not only by the polymerase(s) but also by competing deadenylases such as PARN and TOE1.*

Page 19. Maybe delete 'The non-canonical polymerase" in the paragraph heading and throughout?

- *We have deleted this term*

Page 21. "we used direct sequencing to monitor the effect of TENT2 knockout" Maybe write "RNA species-specific". "Direct" could suggest direct RNA sequencing approach using Nanopore platform which is not the case.

- *The referee is correct (see also comment by referee 1). We replaced our confusing use of 'direct' with 'gene-specific'*

Discussion. The authors concentrate on A-tailing (by investigating its consequences for RN7SL) but U-tailing is important in their study. I suggest they briefly discuss where in the cell does the uridylation they observe occur? In the nucleus or cytoplasm, what enzymes catalyse the uridylation, as the only known nuclear uridyltransferase is TENT1/ U6 TUTase? Given, the claims of U6 TUTase as adenyltransferase (star-PAP) is it possible it monoadenylates RNAs in the absence of TENT2?

- *We agree that the source of the observed U-tailing is an important and interesting question. We analyzed published TUT4/7 (Lim et al, Cell, 2014) and TUT1 (Haas et al, Nucl Acids Res, 2016) depletion RNA 3' end sequencing datasets, but while the TUT4/7 depletion dataset shows evidence that vault RNAs are targets of TUT4/7 it is impossible to make general conclusions because most RNAs are present in only one of the two provided sequencing repeats in the TUT4/7 depletion dataset and the TUT1 depletion dataset is selected for miRNA-sized RNAs. Given the major effort required to reproduce these experiments with an RNA size-selection suitable for this study, we feel that addressing this question is beyond the scope of the current manuscript. We have added the following sentence to the Discussion: 'The source of this uridylation is an important question for future study and may involve TUT1 in the nucleus and/or TUT4/7 in the*

cytoplasm.' With regards to the possibility that STAR-PAP (TUT1) adenylates 7SL RNAs, see our response to the first comment by reviewer 2 above.

The authors are requested to discuss possible ways of degradation of tailed sncRNAs. Specifically, if transient tailing is not significantly affecting 3' trimming of sncRNA then how are these unstable RNA removed? From the 5' end by XRN1/2, or does it occur instantly and cannot be seen in the data?

- *The referee raises an important question on the mechanism of degradation of A-tailed sno/scaRNAs. However, our genome-wide sequencing experiments have insufficient depth to confidently identify degradation intermediates and provide clarity on this issue. While we do observe 5' and 3'-end truncated RNAs in these assays, additional experiments, which would require manipulation of degradation factors, would be needed to determine whether such truncated RNAs are due to cellular degradation or post-lysis hydrolysis of RNAs. We feel this is beyond the scope of the current manuscript.*

1st Revision - Editorial Decision 29th Sep 2025

Dear Dr. Lykke-Andersen,

Thank you for submitting a revised version of your manuscript. Your study has now been seen by all original referees, who find that their previous concerns have been addressed and now recommend publication of the manuscript. There remain only a few mainly editorial points that have to be addressed before I can extend formal acceptance of the manuscript:

- Please adjust the format of the reference list and of the in-text citations according to EMBO Journal format (alphabetical order, author name et al + year.../up to 10 author names in the reference list before et al / please refer to our Guide to Authors for additional information on EMBO J reference format).

- Please rename the Conflict of Interest section into "Disclosure and Competing Interests Statement", in accordance with our updated Guide to Authors (https://www.embopress.org/competing-interests)

- As we are switching from a free-text author contribution statement towards a more formal statement based on Contributor Role Taxonomy (CRediT) terms, please remove the present Author Contribution section and instead specify each author's contribution(s) directly in the Author Information page of our submission system during upload of the final manuscript. See https://casrai.org/credit/ for more information.

- DATASET EV LEGENDS: source file names, titles, legends and manuscript callouts all need to be updated to Dataset EV1-EV5 instead of Tables EV1-EV4 and EV6, legends should be uploaded as a separate tab/sheet in each Excel file; Tables EV5 and EV7 should be renamed to Table EV1-EV2 with the legends included above each table in the Excel file and appropriate callouts

- APPENDIX 1 FILE WITH ToC: missing ToC with the page numbers for the listed items

- R&T TABLE: in ms file; please upldd as a separate file using the template from our GTA

- Please provide suggestions for a short 'blurb' text prefacing and summing up the conceptual aspect of the study in two sentences (max. 250 characters), followed by 3-5 one-sentence 'bullet points' with brief factual statements of key results of the paper; they will form the basis of an editor-written 'Synopsis' accompanying the online version of the article. Please also provide an altered synopsis image, making sure that the aspect ratio conforms to our website's format - it should be exactly 550 pixels wide and between 300-600 pixels high.

- Please note that n=2 in figures EV4 B. Please remove the statistical analysis and only display data points.

- Please indicate the statistical test used for data analysis in the legends of figures 2D, EV2 A, B; EV3 A

- "This PDF file includes:" should be removed from the title page

- "MATERIAL AND METHODS" should be renamed to "Methods"

Please let me know if you have any questions regarding any of these points. You can use the link below to upload the revised files.
Thank you again for giving us the chance to consider your manuscript for The EMBO Journal. I look forward to receiving the final version.
With best regards,
Cornelius Schneider

Cornelius Schneider, PhD
Editor | The EMBO Journal
c.schneider@embojournal.org

Please refer to our figure preparation guideline in order to ensure proper formatting and readability in print as well as on screen:

https://bit.ly/EMBOPressFigurePreparationGuideline

See also figure legend guidelines:

https://www.embopress.org/page/journal/14602075/authorguide#figureformat

----

Referee #1:

The revision from Ocheltree et al. addresses all points raised in the review. I would like to congratulate the authors on their careful and thoughtful revisions, which have considerably strengthened the manuscript. I have only one minor point that could be clarified to aid the reader in following their model.

In relation to Fig1E: This is slightly clearer for the snoRNAs. However, for RNAPIII RNAs - the legend says that they retain their monoadenylated 3' ends. But the figure says 'A-tailing accumulates'. This could either be interpreted as the tail getting longer or that mono-A tailing is more widespread I assume the intention is that the increase in 'A' size reflects greater prevalence in newly transcribed versus steady-state RNAs, but as drawn it is not a clear schematic. I suggest refining the figure to make any distinction more apparent to the reader.

The authors have addressed all minor editorial requests.

Dear Dr. Lykke-Andersen,

I am pleased to inform you that your manuscript has been accepted for publication in the EMBO Journal.

Your manuscript will be processed for publication by EMBO Press. It will be copy edited and you will receive page proofs prior to publication. Please note that you will be contacted by Springer Nature Author Services to complete licensing and payment information.

You may qualify for financial assistance for your publication charges - either via a Springer Nature fully open access agreement or an EMBO initiative. Check your eligibility: https://www.embopress.org/page/journal/14602075/authorguide#chargesguide

Should you be planning a Press Release on your article, please get in contact with embo_production@springernature.com as early as possible in order to coordinate publication and release dates.

If you have any questions, please do not hesitate to contact the Editorial Office. Thank you for your contribution to The EMBO Journal.

Yours sincerely,

Cornelius Schneider, PhD
Editor
The EMBO Journal
c.schneider@embojournal.org

*****
Please note that it is The EMBO Journal policy for the transcript of the editorial process (containing referee reports and your response letters) to be published as an online supplement to each paper. If you should prefer removal of any referee-only figures included in the point-by-point response(s), e.g. because they may still be used for future publication or because they have been reproduced from published work by others, please do let us know immediately via response email.
More information is available here: https://www.embopress.org/transparent-process#Review_Process
*****

EMBO Press Author Checklist

Corresponding Author Name: Jens Lykke-Andersen	
Journal Submitted to: The EMBO Journal	
Manuscript Number: EMBOJ-2025-120731	

USEFUL LINKS FOR COMPLETING THIS FORM
The EMBO Journal - Author Guidelines
EMBO Reports - Author Guidelines
Molecular Systems Biology - Author Guidelines
EMBO Molecular Medicine - Author Guidelines

Reporting Checklist for Life Science Articles (updated January
This checklist is adapted from Materials Design Analysis Reporting (MDAR) Checklist for Authors. MDAR establishes a minimum set of requirements in transparent reporting in the life sciences (see Statement of Task: 10.31222/osf.io/9sm4x). Please follow the journal's guidelines in preparing your
Please note that a copy of this checklist will be published alongside your article.

Abridged guidelines for figures
1. Data
The data shown in figures should satisfy the following conditions:
- ➞ the data were obtained and processed according to the field's best practice and are presented to reflect the results of the experiments in an accurate and unbiased manner.
- ➞ ideally, figure panels should include only measurements that are directly comparable to each other and obtained with the same assay.
- ➞ plots include clearly labeled error bars for independent experiments and sample sizes. Unless justified, error bars should not be shown for technical
- ➞ if n<5, the individual data points from each experiment should be plotted. Any statistical test employed should be justified.
- ➞ Source Data should be included to report the data underlying figures according to the guidelines set out in the authorship guidelines on Data

2. Captions
Each figure caption should contain the following information, for each panel where they are relevant:
- ➞ a specification of the experimental system investigated (eg cell line, species name).
- ➞ the assay(s) and method(s) used to carry out the reported observations and measurements.
- ➞ an explicit mention of the biological and chemical entity(ies) that are being measured.
- ➞ an explicit mention of the biological and chemical entity(ies) that are altered/varied/perturbed in a controlled manner.
- ➞ the exact sample size (n) for each experimental group/condition, given as a number, not a range;
- ➞ a description of the sample collection allowing the reader to understand whether the samples represent technical or biological replicates (including how many animals, litters, cultures, etc.).
- ➞ a statement of how many times the experiment shown was independently replicated in the laboratory.
- ➞ definitions of statistical methods and measures:
  - common tests, such as t-test (please specify whether paired vs. unpaired), simple χ2 tests, Wilcoxon and Mann-Whitney tests, can be unambiguously identified by name only, but more complex techniques should be described in the methods section;
  - are tests one-sided or two-sided?
  - are there adjustments for multiple comparisons?
  - exact statistical test results, e.g., P values = x but not P values < x;
  - definition of 'center values' as median or average;
  - definition of error bars as s.d. or s.e.m.

Please complete ALL of the questions below.
Select "Not Applicable" only when the requested information is not relevant for your study.

Materials

Newly Created Materials	Information included in the manuscript?	In which section is the information available? (Reagents and Tools Table, Materials and Methods, Figures, Data Availability Section)
New materials and reagents need to be available; do any restrictions apply?	Yes	Reagents and Tools Table, Expanded View Tables EV1, EV3

Antibodies	Information included in the manuscript?	In which section is the information available? (Reagents and Tools Table, Materials and Methods, Figures, Data Availability Section)
For **antibodies** provide the following information:<br>- Commercial antibodies: RRID (if possible) or supplier name, catalogue number and or/clone number<br>- Non-commercial: RRID or citation	Yes	Reagents and Tools Table

DNA and RNA sequences	Information included in the manuscript?	In which section is the information available? (Reagents and Tools Table, Materials and Methods, Figures, Data Availability Section)
**Short novel DNA or RNA including primers, probes**: provide the sequences.	Yes	Reagents and Tools Table, Expanded View Table EV1

Cell materials	Information included in the manuscript?	In which section is the information available? (Reagents and Tools Table, Materials and Methods, Figures, Data Availability Section)
**Cell lines:** Provide species information, strain. Provide accession number in repository **OR** supplier name, catalog number, clone number, and/**OR** RRID.	Yes	Reagents and Tools Table
**Primary cultures:** Provide species, strain, sex of origin, genetic modification status.	Not Applicable	
Report if the cell lines were recently **authenticated** (e.g., by STR profiling) and tested for mycoplasma contamination.	Yes	Materials and Methods

Experimental animals	Information included in the manuscript?	In which section is the information available? (Reagents and Tools Table, Materials and Methods, Figures, Data Availability Section)
**Laboratory animals or Model organisms:** Provide species, strain, sex, age, genetic modification status. Provide accession number in repository **OR** supplier name, catalog number, clone number, **OR** RRID.	Not Applicable	
**Animal observed in or captured from the field:** Provide species, sex, and age where possible.	Not Applicable	
Please detail **housing and husbandry conditions**.	Not Applicable	

Plants and microbes	Information included in the manuscript?	In which section is the information available? (Reagents and Tools Table, Materials and Methods, Figures, Data Availability Section)
**Plants:** provide species and strain, ecotype and cultivar where relevant, unique accession number if available, and source (including location for collected wild specimens).	Not Applicable	
**Microbes:** provide species and strain, unique accession number if available, and source.	Not Applicable	

Human research participants	Information included in the manuscript?	In which section is the information available? (Reagents and Tools Table, Materials and Methods, Figures, Data Availability Section)
If collected and within the bounds of privacy constraints report on age, sex and gender or ethnicity for all study participants.	Not Applicable	

Core facilities	Information included in the manuscript?	In which section is the information available? (Reagents and Tools Table, Materials and Methods, Figures, Data Availability Section)
If your work benefited from core facilities, was their service mentioned in the acknowledgments section?	Yes	Acknowledgements

Design

Study protocol	Information included in the manuscript?	In which section is the information available? (Reagents and Tools Table, Materials and Methods, Figures, Data Availability Section)
If study protocol has been **pre-registered, provide DOI in the manuscript**. For clinical trials, provide the trial registration number **OR** cite DOI.	Not Applicable	
Report the **clinical trial registration number** (at ClinicalTrials.gov or equivalent), where applicable.	Not Applicable	

Laboratory protocol	Information included in the manuscript?	In which section is the information available? (Reagents and Tools Table, Materials and Methods, Figures, Data Availability Section)
Provide DOI OR other citation details if **external detailed step-by-step protocols** are available.	Not Applicable	

Experimental study design and statistics	Information included in the manuscript?	In which section is the information available? (Reagents and Tools Table, Materials and Methods, Figures, Data Availability Section)
Include a statement about **sample size** estimate even if no statistical methods were used.	Yes	Figure Legends
Were any steps taken to minimize the effects of subjective bias when allocating animals/samples to treatment (e.g. **randomization procedure**)? If yes, have they been described?	Not Applicable	
Include a statement about **blinding** even if no blinding was done.	Yes	Materials and Methods
Describe **inclusion/exclusion criteria** if samples or animals were excluded from the analysis. Were the criteria pre-established?<br><br>If sample or data points were omitted from analysis, report if this was due to attrition or intentional exclusion and provide justification.	Not Applicable	
For every figure, are **statistical tests** justified as appropriate? Do the data meet the assumptions of the tests (e.g., normal distribution)? Describe any methods used to assess it. Is there an estimate of variation within each group of data? Is the variance similar between the groups that are being statistically compared?	Yes	Figure Legends

Sample definition and in-laboratory replication	Information included in the manuscript?	In which section is the information available? (Reagents and Tools Table, Materials and Methods, Figures, Data Availability Section)
In the figure legends: state number of times the experiment was **replicated** in laboratory.	Yes	Figure Legends
In the figure legends: define whether data describe **technical or biological replicates**.	Yes	Figure Legends

Ethics

Ethics	Information included in the manuscript?	In which section is the information available? (Reagents and Tools Table, Materials and Methods, Figures, Data Availability Section)
Studies involving **human participants**: State details of **authority granting ethics approval** (IRB or equivalent committee(s), provide reference number for approval.	Not Applicable	
Studies involving **human participants**: Include a statement confirming that **informed consent** was obtained from all subjects and that the experiments conformed to the principles set out in the WMA Declaration of Helsinki and the Department of Health and Human Services Belmont Report.	Not Applicable	
Studies involving **human participants:** For publication of **patient photos**, include a statement confirming that consent to publish was obtained.	Not Applicable	
Studies involving experimental **animals**: State details of **authority granting ethics approval** (IRB or equivalent committee(s), provide reference number for approval. Include a statement of compliance with ethical regulations.	Not Applicable	
Studies involving **specimen and field samples:** State if relevant **permits** obtained, provide details of authority approving study; if none were required, explain why.	Not Applicable	

Dual Use Research of Concern (DURC)	Information included in the manuscript?	In which section is the information available? (Reagents and Tools Table, Materials and Methods, Figures, Data Availability Section)
Could your study fall under dual use research restrictions? Please check biosecurity documents and list of **select agents and toxins** (CDC): https://www.selectagents.gov/sat/list.htm	Not Applicable	
If you used a select agent, is the security level of the lab appropriate and reported in the manuscript?	Not Applicable	
If a study is subject to dual use research of concern regulations, is the name of the **authority granting approval and reference number** for the regulatory approval provided in the manuscript?	Not Applicable	

Reporting

The MDAR framework recommends adoption of discipline-specific guidelines, established and endorsed through community initiatives. Journals have their own policy about requiring specific guidelines and recommendations to complement MDAR.

Adherence to community standards	Information included in the manuscript?	In which section is the information available? (Reagents and Tools Table, Materials and Methods, Figures, Data Availability Section)
State if relevant guidelines or checklists (e.g., **ICMJE, MIBBI, ARRIVE, PRISMA**) have been followed or provided.	Not Applicable	
For **tumor marker prognostic studies,** we recommend that you follow the **REMARK** reporting guidelines (see link list at top right). See author guidelines, under 'Reporting Guidelines'. Please confirm you have followed these guidelines.	Not Applicable	
For **phase II and III randomized controlled trials**, please refer to the **CONSORT** flow diagram (see link list at top right) and submit the CONSORT checklist (see link list at top right) with your submission. See author guidelines, under 'Reporting Guidelines'. Please confirm you have submitted this list.	Not Applicable	

Data Availability

Data availability	Information included in the manuscript?	In which section is the information available? (Reagents and Tools Table, Materials and Methods, Figures, Data Availability Section)
Have **primary datasets** been deposited according to the journal's guidelines (see 'Data Deposition' section) and the respective accession numbers provided in the Data Availability Section?	Yes	Data Availability
Were **human clinical and genomic datasets** deposited in a public access-controlled repository in accordance to ethical obligations to the patients and to the applicable consent agreement?	Not Applicable	
Are **computational models** that are central and integral to a study available without restrictions in a machine-readable form? Were the relevant accession numbers or links provided?	Not Applicable	
If publicly available data were reused, provide the respective **data citations in the reference list**.	Yes	References
